# Genome-Wide Identification of *Jatropha curcas* Aquaporin Genes and the Comparative Analysis Provides Insights into the Gene Family Expansion and Evolution in *Hevea brasiliensis*

**DOI:** 10.3389/fpls.2016.00395

**Published:** 2016-03-31

**Authors:** Zhi Zou, Lifu Yang, Jun Gong, Yeyong Mo, Jikun Wang, Jianhua Cao, Feng An, Guishui Xie

**Affiliations:** Danzhou Investigation and Experiment Station of Tropical Crops, Ministry of Agriculture, Rubber Research Institute, Chinese Academy of Tropical Agricultural SciencesDanzhou, China

**Keywords:** physic nut (*Jatropha curcas* L.), rubber tree (*Hevea brasiliensis* Muell. Arg.), aquaporin, AQP gene family, expansion, evolution

## Abstract

Aquaporins (AQPs) are channel-forming integral membrane proteins that transport water and other small solutes across biological membranes. Despite the vital role of AQPs, to date, little is known in physic nut (*Jatropha curcas* L., Euphorbiaceae), an important non-edible oilseed crop with great potential for the production of biodiesel. In this study, 32 AQP genes were identified from the physic nut genome and the family number is relatively small in comparison to 51 in another Euphorbiaceae plant, rubber tree (*Hevea brasiliensis* Muell. Arg.). Based on the phylogenetic analysis, the JcAQPs were assigned to five subfamilies, i.e., nine plasma membrane intrinsic proteins (PIPs), nine tonoplast intrinsic proteins (TIPs), eight NOD26-like intrinsic proteins (NIPs), two X intrinsic proteins (XIPs), and four small basic intrinsic proteins (SIPs). Like rubber tree and other plant species, functional prediction based on the aromatic/arginine selectivity filter, Froger's positions, and specificity-determining positions showed a remarkable difference in substrate specificity among subfamilies of JcAQPs. Genome-wide comparative analysis revealed the specific expansion of PIP and TIP subfamilies in rubber tree and the specific gene loss of the XIP subfamily in physic nut. Furthermore, by analyzing deep transcriptome sequencing data, the expression evolution especially the expression divergence of duplicated HbAQP genes was also investigated and discussed. Results obtained from this study not only provide valuable information for future functional analysis and utilization of Jc/HbAQP genes, but also provide a useful reference to survey the gene family expansion and evolution in Euphorbiaceae plants and other plant species.

## Introduction

Aquaporins (AQPs) are channel-forming integral membrane proteins that transport water and other small solutes across biological membranes (Maurel et al., 2008; Gomes et al., 2009). Since their first identification and characterization in 1990s, AQPs have been found in all types of organisms, including microbes, animals, and plants (Gomes et al., 2009; Abascal et al., 2014). Although the overall sequence similarity can be low, AQPs are characterized by six transmembrane helices (TM1–TM6) connected by five loops (LA–LE), two half helices (HB and HE) formed by the opposite LB and LE dipping into the membrane, two NPA (Asn-Pro-Ala) motifs (located at the N-termini of HB and HE) and the aromatic/arginine (ar/R) selectivity filter (named H2, H5, LE1, and LE2) that determine the substrate specificity (Fu et al., 2000; Sui et al., 2001; Törnroth-Horsefield et al., 2006). Compared with microbes and animals, genome-wide surveys showed that AQPs are highly abundant and diverse in high plants (Table 1). According to the sequence similarity, plant AQPs can be divided into five main subfamilies, i.e., plasma membrane intrinsic proteins (PIPs), tonoplast intrinsic proteins (TIPs), NOD26-like intrinsic proteins (NIPs), small basic intrinsic proteins (SIPs), and uncategorized X intrinsic proteins (XIPs). Interestingly, the newly identified XIP subfamily has been found only in dicots beyond the Brassicaceae family (Johanson et al., 2001; Gupta and Sankararamakrishnan, 2009; Tao et al., 2014; Diehn et al., 2015). Corresponding to the high degree of compartmentalization of plant cells, plant AQPs are localized in the plasma membrane, tonoplasts/vacuoles, plastids, mitochondria, endoplasmic reticulum, Golgi apparatus, and in some species, in membrane compartments interacting with symbiotic organisms (Wudick et al., 2009; Udvardi and Poole, 2013). In addition to water, function studies showed that plant AQPs also transport glycerol, urea, ammonia (NH_3_), carbon dioxide (CO_2_), hydrogen peroxide (H_2_O_2_), and metalloids such as boron and silicon (Maurel et al., 2008; Gomes et al., 2009; Pommerrenig et al., 2015).

**Table 1:** **Diversity of AQP gene family in high plants**.

**Species**	**Common name**	**Family**	**Type of organism**	**PIPs**	**TIPs**	**NIPs**	**SIPs**	**XIPs**	**Total**	**References**
*Oryza sativa*	Rice	Poaceae	Monocot	11	10	10	2	0	33	Sakurai et al., 2005
*Zea mays*	Maize	Poaceae	Monocot	13	12	5	3	0	33	Chaumont et al., 2001
*Hordeum vulgare*	Barley	Poaceae	Monocot	20	11	8	1	0	40	Hove et al., 2015
*Musa acuminate*	Banana	Musaceae	Monocot	18	17	9	3	0	47	Hu W. et al., 2015
*Arabidopsis thaliana*	Arabidopsis	Brassicaceae	Dicot	13	10	9	3	0	35	Johanson et al., 2001; Quigley et al., 2002;
*Brassica rapa*	Chinese cabbage	Brassicaceae	Dicot	23	16	15	6	0	60	Tao et al., 2014; Diehn et al., 2015
*Brassica oleracea*	Cabbage	Brassicaceae	Dicot	25	19	17	6	0	67	Diehn et al., 2015
*Solanum tuberosum*	Pomato	Solanaceae	Dicot	15	11	10	3	8	47	Venkatesh et al., 2013
*Solanum lycopersicum*	Garden tomato	Solanaceae	Dicot	14	11	12	4	6	47	Reuscher et al., 2013
*Glycine max*	Soybean	Fabaceae	Dicot	22	23	17	8	2	72	Deshmukh et al., 2013; Zhang et al., 2013
*Gossypium hirsutum*	Upland cotton	Malvaceae	Dicot	28	23	12	7	1	71	Park et al., 2010
*Vitis vinifera*	Grapevine	Vitaceae	Dicot	8	11	9	2	2	32	Jaillon et al., 2007; Shelden et al., 2009
*Citrus sinensis*	Sweet orange	Rutaceae	Dicot	8	11	9	3	3	34	de Paula Santos Martins et al., 2015
*Phaseolus vulgaris*	Common bean	Fabaceae	Dicot	12	13	10	4	2	41	Ariani and Gepts, 2015
*Jatropha curcas*	Physic nut	Euphorbiaceae	Dicot	9	9	8	4	2	32	This study
*Ricinus communis*	Castor bean	Euphorbiaceae	Dicot	10	9	8	4	6	37	Zou et al., 2015b
*Hevea brasiliensis*	Rubber tree	Euphorbiaceae	Dicot	15	17	9	4	6	51	Zou et al., 2015a
*Populus trichocarpa*	Poplar	Salicaceae	Dicot	15	17	11	6	6	55	Gupta and Sankararamakrishnan, 2009

Euphorbiaceae is one of the largest plant family, which consists of more than 7000 species characterized with high photosynthesis and high biomass (Endress et al., 2013). There are many economically important species in Euphorbiaceae, such as rubber tree (*Hevea brasiliensis* Muell. Arg.), castor bean (*Ricinus communis* L.) and physic nut (*Jatropha curcas* L.). Rubber tree, also known as Para or Brazilian rubber tree, is a perennial big tree native to the Amazon basin. The natural rubber (*cis*-1,4-polyisoprene), produced by the rubber tree laticifer (a highly differentiated single-cell-type tissue located in the secondary phloem of the tree trunk), is an essential industrial raw materials for tires and other products (Zou et al., 2009; Prabhakaran Nair, 2010). Castor bean is a perennial shrub that originated from Africa. The oil extracted from castor seeds is mainly composed of the unusual hydroxylated fatty acid ricinoleic acid and thus widely used for industrial, medicinal, and cosmetic purposes (Ogunniyi, 2006). Physic nut is a perennial shrub or small tree native to central America and now is widely cultivated in many tropical and subtropical countries in Asia and Africa (Montes Osorio et al., 2014). As a non-food oilseed crop, physic nut has great potential for the production of biodiesel, which features high seed oil content (up to 50%), fossil fuel-like oil composition (unsaturated fatty acids >75%) and adaptation to semiarid and barren soil environments (Fairless, 2007; Montes Osorio et al., 2014). Thus far, the genome sequences of these three diploid plant species (Arumuganathan and Earle, 1991; Leitch et al., 1998; Carvalhoa et al., 2008) were all obtained through whole genome sequencing (Chan et al., 2010; Sato et al., 2011; Rahman et al., 2013; Wu et al., 2015). The genome size of castor bean is approximate 400 Mb, and the 4.6 × draft genome available consists of 25,878 scaffolds containing 31,221 putative protein-coding genes (Chan et al., 2010). The genome size of physic nut is about 350 Mb, and two assembled genomes of a line originating from the Palawan Island and an inbred cultivar GZQX0401 have been reported (Sato et al., 2011; Wu et al., 2015). The draft genome reported by Sato et al. (2011) is 285,858,490 bp consisting of 120,586 contigs and 29,831 singlets, and a number of 40,929 complete and partial structures of protein encoding genes have been deduced. Later, 537 million paired-end Illumina reads were integrated and the length of the upgraded genome sequences reached 297,661,187 bp consisting of 39,277 scaffolds (Hirakawa et al., 2012). The more complete genome assembly reported by Wu et al. (2015) is 320,546,307 bp consisting of 72,474 contigs longer than 100 bp, and the contigs were further assembled into 23,125 scaffolds with an N50 of 0.746 Mbp which is considerably longer than that of the previous reported one (0.016 Mbp). As a result, the number of putative protein-encoding genes was reduced from 30,203 (Hirakawa et al., 2012) to 27,172 (Wu et al., 2015) since more genes are complete. By comparison, the genome size of rubber tree is considerably larger and the reported assembly spans about 1.1 Gb of the estimated 2.15 Gb haploid genome (Bennett and Leitch, 1997; Rahman et al., 2013). In agreement with this, the number of predicted gene models in rubber tree is 68,955, which is more than two-folds of that in castor bean and physic nut (Chan et al., 2010; Rahman et al., 2013; Wu et al., 2015). Since both castor bean and physic nut underwent no recent whole-genome duplication (WGD) event (Chan et al., 2010; Wu et al., 2015), the duplicated rubber tree genes are more likely to be resulted from an unknown recent doubling event. Lately, two papers reported the identification of AQP genes encoded in the genomes of rubber tree and castor bean (Zou et al., 2015a,b). The family number of 51 in rubber tree (Zou et al., 2015a) is comparable to 55 in poplar (Gupta and Sankararamakrishnan, 2009), a Salicaceae tree species also belongs to Malpighiales which was shown to undergo a recent doubling event (Tuskan et al., 2006). In contrast, castor bean contains as few as 37 family members. Although the evolutionary relationship of AQPs between rubber tree and castor bean is not investigated yet, the classification of subfamily and even subfamily into subgroups was shown to be the same: the PIP subfamily contains two subgroups; the TIP subfamily contains five subgroups; the NIP subfamily contains seven subgroups; the SIP subfamily contains two subgroups; and the XIP subfamily contains three subgroups (Zou et al., 2015a,b). Compared with rubber tree and castor bean, the molecular characterization of physic nut AQPs (JcAQPs) is still in its infancy. As of Sep 2015, only two full-length AQP cDNAs have been reported (Zhang et al., 2007; Jang et al., 2013; Khan et al., 2015). The available genome and several tissue transcriptome datasets (King et al., 2011; Natarajan and Parani, 2011; Sato et al., 2011; Hirakawa et al., 2012; Jiang et al., 2012; Wang H. et al., 2013; Juntawong et al., 2014; Pan et al., 2014; Zhang et al., 2014, 2015; Wu et al., 2015) provide an opportunity to analyze the physic nut AQP gene family from a global view.

In this study, a genome-wide search was carried out to identify the physic nut AQP genes. Functional prediction was performed based on the ar/R filter (i.e., H2 in TM2, H5 in TM5, LE1 and LE2 in LE) (Törnroth-Horsefield et al., 2006), Froger's positions (five conserved amino acid residues named P1–5 for discriminating glycerol-transporting aquaglyceroporins from water-conducting AQPs) (Froger et al., 1998) and specificity-determining positions (SDP1–SDP9, important for determining the specificity of non-aqua substrates) (Hove and Bhave, 2011), and their expression profiles were examined using deep transcriptome sequencing data. Furthermore, their evolutionary relationships with HbAQPs and RcAQPs as well as the expression evolution of the duplicated HbAQP genes were also investigated.

## Materials and methods

### Identification of JcAQP genes

The AQPs in *Arabidopsis* (Johanson et al., 2001), poplar (Gupta and Sankararamakrishnan, 2009), rubber tree (Zou et al., 2015a), and castor bean (Zou et al., 2015b) described before were obtained according to the literatures (the accession number can be found in Supplementary Table S1). The genome sequences, nucleotides, Sanger ESTs (expressed sequence tags), and raw RNA sequencing reads were downloaded from NCBI GenBank or SRA (sequence read archive) databases, respectively (http://www.ncbi.nlm.nih.gov/). The deduced amino acid sequences of published JcAQP genes (Zhang et al., 2007; Jang et al., 2013) were used as queries to search the physic nut genome (Sato et al., 2011; Wu et al., 2015) for homologs. Sequences with an *E* < 1e^−5^ in the tBlastn search (Altschul et al., 1997) were selected for further analysis. The predicted gene models were checked with ESTs and RNA sequencing reads, and the gene structures were displayed using GSDS (Hu B. et al., 2015). Homology search was performed using Blastn (Altschul et al., 1997) and ESTs with the identity of more than 98% were taken into account. RNA sequencing data from callus, root, leaf, flower, inflorescence meristem, seed, and embryo described before (King et al., 2011; Natarajan and Parani, 2011; Sato et al., 2011; Hirakawa et al., 2012; Jiang et al., 2012; Wang H. et al., 2013; Juntawong et al., 2014; Pan et al., 2014; Zhang et al., 2014, 2015; Wu et al., 2015) were also adopted for the expression annotation to determine whether genes are expressed. The clean reads were obtained by removing adaptor sequences, adaptor-only reads, and low quality reads containing more than 50% bases with *Q* ≤ 5. Read mapping was performed using Bowtie 2 (Langmead and Salzberg, 2012) with default parameters, and mapped read number of more than one was counted as expressed. The alternative splicing isoforms were identified using Cufflinks (v2.2.1) (Trapnell et al., 2012). The ortholog of each JcAQP in *Arabidopsis*, poplar, rubber tree, and castor bean was identified using Blastp (Altschul et al., 1997) (*E*-value, 1e^−20^) against AtAQPs, PtAQPs, HbAQPs, and RcAQPs, and the best hit was collected.

### Sequence alignments, phylogenetic analysis, and classification

Multiple sequence alignments of deduced peptides were carried out using ClustalX (Thompson et al., 1994), and the unrooted phylogenetic tree was constructed using MEGA6 (Tamura et al., 2013) with the maximum likelihood method (bootstrap: 1000). Classification of AQPs into subfamilies and subgroups was done as described before (Zou et al., 2015a) and the systematic names were assigned based on their evolutionary relationships.

### Structural features of JcAQPs

Protein properties such as the molecular weight (MW) and isoelectric point (*pI*), were calculated using ProtParam (http://web.expasy.org/protparam/). The subcellular localization and transmembrane regions were predicted using WoLF PSORT (Horton et al., 2007) and TOPCONS (Bernsel et al., 2009), respectively. Functional prediction was performed based on dual NPA motifs, the ar/R filter, five Froger's positions and nine SDPs from alignments with the structure resolved *Spinacia oleracea* PIP2;1 and functionally characterized AQPs (Froger et al., 1998; Törnroth-Horsefield et al., 2006; Hove and Bhave, 2011).

### Gene expression analysis

To investigate the global gene expression profiles among different tissues, Illumina RNA sequencing data derived from physic nut root (NCBI SRA accession number SRX750579), leaf (SRX750580), and seed (SRX750581) (Wu et al., 2015) as well as rubber tree laticifer (SRX278514), bark (SRX278513), and leaf (SRX278515) (Chow et al., 2014) described before was examined. The obtained clean reads were mapped to the CDS of 32 *JcAQPs* and 51 *HbAQPs* as well as available transcripts using Bowtie 2 (Langmead and Salzberg, 2012), and the FPKM (fragments per kilobase of exon per million fragments mapped) method (Mortazavi et al., 2008) was used for the determination of transcript levels. Unless specific statements, the tools used in this study were performed with default parameters.

## Results

### Identification and classification of JcAQP genes

The homology search resulted in 32 loci putatively encoding AQP-like genes from both assembled physic nut genomes. Since all AQP-encoding loci identified in the Palawan genome were found in the genome of GZQX0401 but some genes from Palawan are incomplete, the AQP genes from GZQX0401 were selected for further analyses (Table 2). Among them, 31 loci were predicted by the automatic genome annotation (Wu et al., 2015), whereas one more locus putatively encodes a SIP subfamily member (i.e., *JcSIP1;1*) was identified from the scaffold2033 (GenBank accession number KK916495). Read alignments indicated that the transcriptional region of this gene is 5368 bp, including two introns (590 and 3167 bp, respectively), 329-bp 5′ UTR and 562-bp 3′ UTR (see Supplementary File S1). The gene structure is also supported by two ESTs (GenBank accession numbers, FM894285 and GW875379). Sequence alignments showed that most predicted gene models of JcAQPs were validated with ESTs and/or RNA sequencing reads (Table 2), however, three loci (i.e., JCGZ_02114, JCGZ_19604, and JCGZ_01828) seem not to be properly annotated. The locus JCGZ_02114 (*JcNIP7;1*) was predicted to harbor four introns encoding 618 residues (Wu et al., 2015) which is considerably longer than that of any other NIP subfamily members, however, sequence alignment showed that the N- and C-termini of the deduced protein are homologous with eukaryotic aspartyl protease family proteins and NOD26-like intrinsic proteins, i.e., AT1G03220 and AT3G06100 (AtNIP7;1) in *Arabidopsis*, respectively. Further read alignments supported the existence of two genes: the first one contains no intron and putatively encodes an aspartyl protease of 451 residues, and the second one harbors four introns encoding an NIP of 265 residues (Supplementary File S2). The locus JCGZ_19604 (*JcXIP1;1*) was predicted to encode 271 residues, which is relatively shorter than 289 residues of its ortholog in rubber tree (Zou et al., 2015a), thus we carefully investigated these two genes and found that a number of 67-bp sequences toward the 5′ of the CDS missed from the genome annotation (Supplementary File S3). Thereby, the CDS length of this locus was extended to 885 bp putatively encoding 294 residues (Supplementary File S3). The locus JCGZ_01828 (*JcSIP1;2*) was predicted to contain no intron encoding 235 residues, however, read alignments showed that the transcriptional region of this gene is 992 bp putatively encoding 242 residues (Supplementary File S4).

**Table 2:** **List of the 32 JcAQP genes identified in this study**.

**Name**	**Scaffold ID**	**Locus ID**	**Predicted position**	**Identified position**	**Chr**	**Nucleotide length (bp, from start to stop codons)**		**EST hits**	**Expressed**	**Alternative splicing**		**AtAQP ortholog**	**PtAQP ortholog**	**RcAQP ortholog**	**HbAQP ortholog**
						**CDS**	**Gene**			**EST**	**Read**				
*JcPIP1;1*	Scaffold11	JCGZ_01316	1070240–1071401	1069930–1071930	LG10	864	1162	66	Yes	–	–	AtPIP1;1	PtPIP1;3	RcPIP1;3	HbPIP1;2
*JcPIP1;2*	Scaffold660	JCGZ_20580	1435150–1432165	1435625–1431654	LG9	864	2986	26	Yes	Yes	Yes	AtPIP1;1	PtPIP1;3	RcPIP1;4	HbPIP1;4
*JcPIP1;3*	Scaffold843	JCGZ_25040	422332–423912	422332–423912	LG6	639	1581	–	–	–	–	AtPIP1;1	PtPIP1;5	RcPIP1;1	HbPIP1;1
*JcPIP1;4*	Scaffold392	JCGZ_14388	3034258–3032720	3034588–3032444	LG8	861	1539	1	Yes	–	Yes	AtPIP1;1	PtPIP1;5	RcPIP1;5	HbPIP1;5
*JcPIP2;1*	Scaffold473	JCGZ_16499	528238–527109	528393–526820	LG6	855	1185	17	Yes	–	–	AtPIP2;4	PtPIP2;7	RcPIP2;1	HbPIP2;1
*JcPIP2;2*	Scaffold18	JCGZ_05520	1671011–1673093	1670693–1673533	LG2	861	1654	9	Yes	–	Yes	AtPIP2;4	PtPIP2;8	RcPIP2;3	HbPIP2;4
*JcPIP2;3*	Scaffold63	JCGZ_20043	224967–221774	225179–220824	LG10	858	3194	7	Yes	–	Yes	AtPIP2;5	PtPIP2;4	RcPIP2;2	HbPIP2;6
*JcPIP2;4*	Scaffold540	JCGZ_18836	689605–690883	689338–691284	LG2	843	1939	50	Yes	–	Yes	AtPIP2;8	PtPIP2;2	RcPIP2;4	HbPIP2;7
*JcPIP2;5*	Scaffold872	JCGZ_25357	143964–140778	144178–140525	LG9	852	3187	4	Yes	–	Yes	AtPIP2;8	PtPIP2;9	RcPIP2;4	HbPIP2;9
*JcTIP1;1*	Scaffold473	JCGZ_16577	1075843–1074990	1076252–1074449	LG6	759	854	186	Yes	Yes	Yes	AtTIP1;1	PtTIP1;6	RcTIP1;1	HbTIP1;2
*JcTIP1;2*	Scaffold528	JCGZ_18448	657196–655908	657291–655693	LG10	759	1289	–	Yes	–	Yes	AtTIP1;3	PtTIP1;1	RcTIP1;2	HbTIP1;5
*JcTIP1;3*	Scaffold18	JCGZ_05655	2916885–2918031	2916391–2919915	LG2	759	1070	–	Yes	–	–	AtTIP1;3	PtTIP1;1	RcTIP1;3	HbTIP1;7
*JcTIP1;4*	Scaffold18	JCGZ_05430	531115–532094	531009–532425	LG2	765	980	–	Yes	–	–	AtTIP1;1	PtTIP1;7	RcTIP1;4	HbTIP1;3
*JcTIP2;1*	Scaffold191	JCGZ_06324	1941464–1942389	1941333–1942605	LG2	747	1304	40	Yes	–	–	AtTIP2;1	PtTIP2;2	RcTIP2;1	HbTIP2;1
*JcTIP2;2*	Scaffold137	JCGZ_03415	265803–267097	265749–267564	LG9	753	1295	3	Yes	Yes	Yes	AtTIP2;2	PtTIP2;4	RcTIP2;2	HbTIP2;3
*JcTIP3;1*	Scaffold23	JCGZ_08448	71937–71012	72143–70768	LG8	774	927	28	Yes	–	–	AtTIP3;2	PtTIP3;2	RcTIP3;1	HbTIP3;1
*JcTIP4;1*	Scaffold211	JCGZ_07757	4725255–4724215	4725438–4724041	LG11	744	1041	1	Yes	–	–	AtTIP4;1	PtTIP4;1	RcTIP4;1	HbTIP4;1
*JcTIP5;1*	Scaffold906	JCGZ_26261	2139472–2140440	2138913–2140560	LG5	759	969	–	Yes	–	–	AtTIP5;1	PtTIP5;2	RcTIP5;1	HbTIP5;1
*JcNIP1;1*	Scaffold684	JCGZ_21622	2673636–2675513	2673356–2675705	LG3	828	1878	–	Yes	–	–	AtNIP1;2	PtNIP1;1	RcNIP1;1	HbNIP1;1
*JcNIP2;1*	Scaffold617	JCGZ_19849	96979–94697	97540–94138	LG9	876	2283	–	Yes	–	Yes	–	PtNIP2;1	RcNIP2;1	HbNIP2;1
*JcNIP3;1*	Scaffold46	JCGZ_15791	1520636–1519402	1520800–1519402	LG4	834	1235	–	Yes	–	–	–	PtNIP3;1	RcNIP3;1	HbNIP3;1
*JcNIP3;2*	Scaffold8	JCGZ_24027	126258–127532	126113–127676	LG7	843	1275	–	Yes	–	–	–	PtNIP3;1	RcNIP3;1	HbNIP3;1
*JcNIP4;1*	Scaffold1210	JCGZ_02488	12952–14295	12952–14295	LG9	792	1344	1	Yes	–	–	AtNIP4;2	PtNIP4;2	RcNIP4;2	HbNIP4;2
*JcNIP5;1*	Scaffold660	JCGZ_20348	126085–128887	124382–130375	LG9	897	2803	2	Yes	–	Yes	AtNIP5;1	PtNIP5;1	RcNIP5;1	HbNIP5;1
*JcNIP6;1*	Scaffold96	JCGZ_27003	2282049–2284301	2281766–2285794	LG2	924	2253	–	Yes	Yes	Yes	AtNIP6;1	PtNIP6;1	RcNIP6;1	HbNIP6;1
*JcNIP7;1*	Scaffold119	JCGZ_02114	699621–702542	701200–702613	LG1	798	1124	–	Yes	–	–	AtNIP7;1	PtNIP7;1	RcNIP7;1	HbNIP7;1
*JcXIP1;1*	Scaffold595	JCGZ_19604	291308–292309	291239–292309	LG2	885	1071	–	–	–	–	–	PtXIP1;1	RcXIP1;3	HbXIP1;1
*JcXIP2;1*	Scaffold595	JCGZ_19603	288337–289806	288124–289999	LG2	912	1470	–	Yes	–	Yes	–	PtXIP2;1	RcXIP2;1	HbXIP2;1
*JcSIP1;1*	Scaffold2033	–	–	130442–125075	LG6	720	4477	–	Yes	–	Yes	AtSIP1;1	PtSIP1;2	RcSIP1;1	HbSIP1;3
*JcSIP1;2*	Scaffold1149	JCGZ_01828	248685–247978	248898–247991	LG7	729	729	–	Yes	–	–	AtSIP1;1	PtSIP1;4	RcSIP1;3	HbSIP1;1
*JcSIP1;3*	Scaffold1149	JCGZ_01827	247059–246328	247514–246040	LG7	732	732	–	Yes	–	–	AtSIP1;1	PtSIP1;4	RcSIP1;3	HbSIP1;1
*JcSIP2;1*	Scaffold407	JCGZ_14885	34721–25023	35046–24630	LG6	726	6716	–	Yes	–	Yes	AtSIP2;1	PtSIP2;1	RcSIP2;1	HbSIP2;1

The 32 identified JcAQP genes were found to be distributed across 26 scaffolds. Although most scaffolds harbor only one AQP gene, five scaffolds (i.e., scaffold660, scaffold473, scaffold595, scaffold1149, and scaffold18) were shown to have two or three AQP genes (Table 2). All JcAQP genes can be further assigned to the 11 chromosomes (Wu et al., 2015). Although all these chromosomes contain at least one AQP gene, the distribution of AQP loci seems unevenly. Among six chromosomes encoding more than one AQP locus, chromosome 2 occupies the largest number of 8 (Table 2).

Along with the genome sequences, as of Sep 2015, 46,865 Sanger ESTs derived from cDNA libraries (including flower, seed, endosperm, embryo, and root) and deep transcriptome sequencing data of several tissues such as callus, root, leaf, flower, inflorescence meristem, seed, and embryo were also available in NCBI. Sequence alignments showed that 15 out of 32 JcAQP genes had EST hits in GeneBank, and *JcTIP1;1* matched the maximum number of 186 ESTs. Except for *JcPIP1;3* and *JcXIP1;1*, read alignments further supported the expression of other 15 JcAQP genes. With the exception of *JcPIP1;3, JcXIP1;1*, and *JcNIP4;1*, the transcriptional region of other JcAQP genes was extended. In addition, alternative splicing isoforms existing in 15 AQP-encoding loci were supported by RNA sequencing reads, and four even by Sanger ESTs (i.e., *JcPIP1;2, JcTIP1;1, JcTIP2;2*, and *JcNIP6;1*) (Table 2).

**Figure 1:**
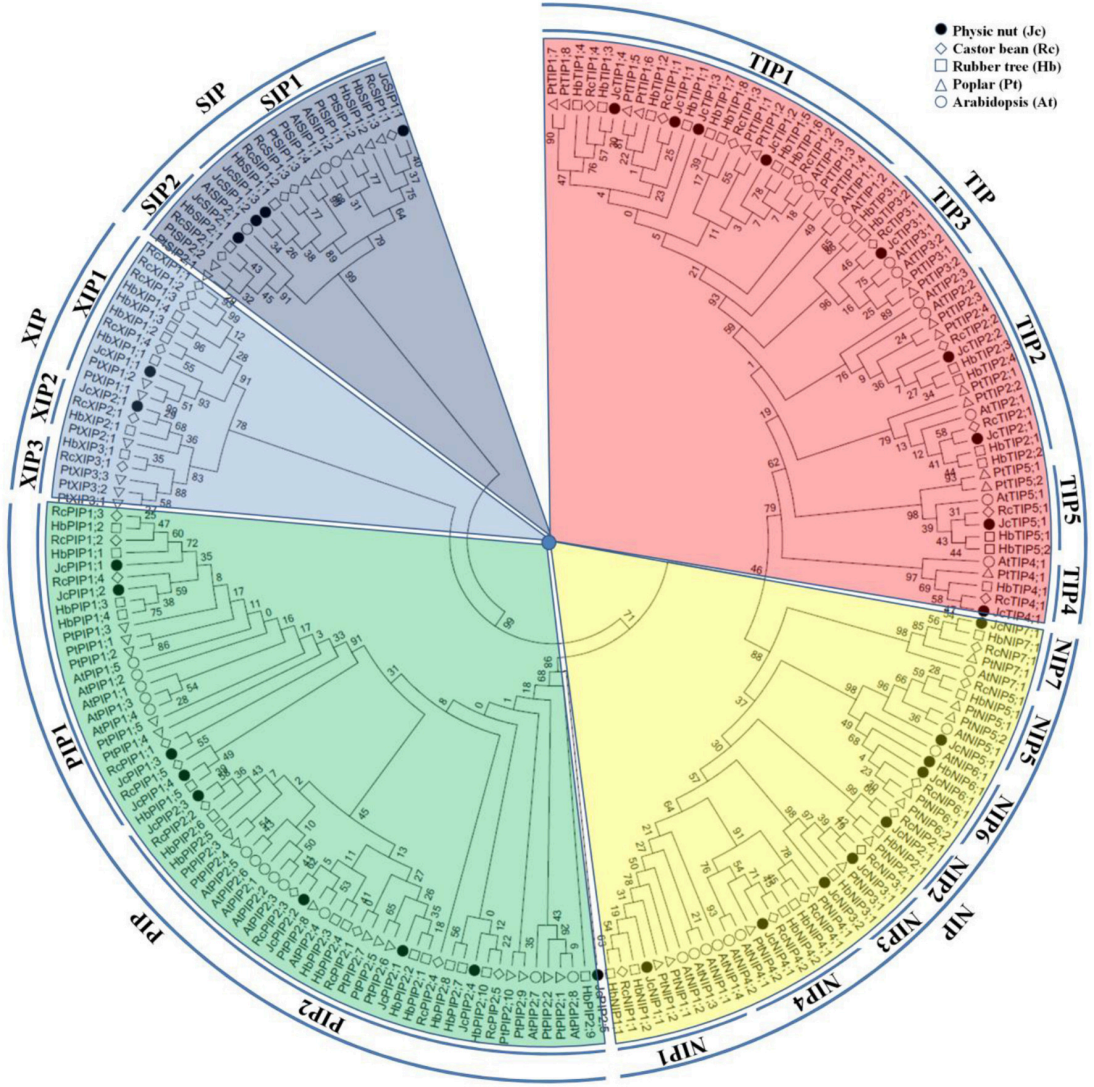
**Phylogenetic analysis of the 32 JcAQPs with ***Arabidopsis***, castor bean, rubber tree, and poplar homologs**. Deduced amino acid sequences were aligned using ClustalX and the phylogenetic tree was constructed using bootstrap maximum likelihood tree (1000 replicates) method and MEGA6 software. The distance scale denotes the number of amino acid substitutions per site. The name of each subfamily and subgroup is indicated next to the corresponding group. Species and accession numbers are listed in Table 2 and Supplementary Table S1.

**Figure 2:**
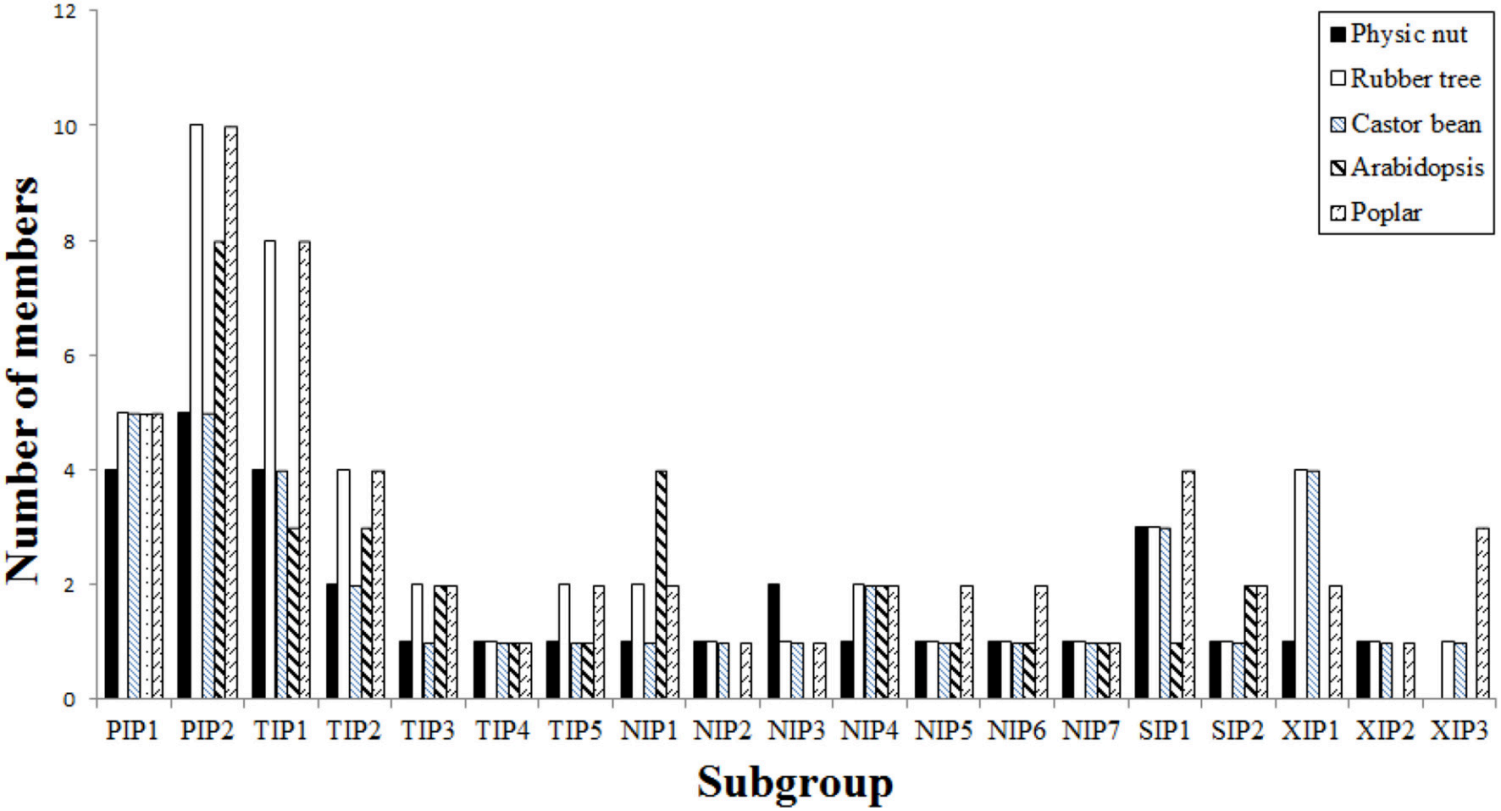
**Distribution of the 32 JcAQP genes and their ***Arabidopsis***, castor bean, rubber tree, and poplar homologs in subgroups**.

To reveal the evolutionary relationship and gain more information about their putative function, an unrooted phylogenetic tree was constructed using MEGA6 from the deduced amino acid sequences of JcAQPs together with that from castor bean (RcAQPs), rubber tree (HbAQPs) and two well-studied model plant species, *Arabidopsis* (AtAQPs) and poplar (PtAQPs) (Figure 1). According to the phylogenetic analysis, the identified JcAQPs were grouped into five subfamilies, i.e., PIP (9), TIP (9), NIP (8), SIP (4), and XIP (2) (Table 2 and Figure 1). Following the nomenclature of rubber tree, the JcPIP subfamily was further divided into two phylogenetic subgroups (4 JcPIP1s and 5 JcPIP2s), the JcTIP subfamily into five subgroups (4 JcTIP1s, 2 JcTIP2s, 1 JcTIP3, 1 JcTIP4, and 1 JcTIP5), the JcNIP subfamily into seven subgroups (1 JcNIP1, 1 JcNIP2, 2 JcNIP3s, 1 JcNIP4, 1 JcNIP5, 1 JcNIP6, and 1 JcNIP7), the JcSIP subfamily into two subgroups (3 JcSIP1s and 1 JcSIP2), and the JcXIP subfamily into two subgroups (1 JcXIP1 and 1 JcXIP2) (Figure 1). It's worth noting that AtNIP2;1 and AtNIP3;1 were assigned into the NIP1 subgroup in this study (Supplementary Table S1), mainly for their closer cluster with the NIP1 subgroup and sharing the highest similarity with NIP1s from physic nut, castor bean, rubber tree, and poplar (Figure 1). Thereby, *Arabidopsis* is shown to lose the NIP2 and NIP3 subgroups as well as the XIP subfamily in comparison to other four plant species (Figures 1, 2). Homology analysis indicated that the 32 JcAQPs have 30 counterparts in rubber tree, 29 in castor bean and 27 in poplar, whereas only 27 out of them have orthologs with a number of 18 in *Arabidopsis* (Table 2 and Figure 1), indicating the expansion and loss of certain AQP genes in castor bean, rubber tree, poplar, and *Arabidopsis*. Indeed, as shown in Figure 1, a high number of *Arabidopsis* (11), poplar (23), and rubber tree (17) AQP genes were grouped in pairs, corresponding to the occurrence of more than one WGD events in these plants (Bowers et al., 2003; Tuskan et al., 2006; Zou et al., 2015a). In contrast, very few AQP gene pairs were identified in physic nut (2) and castor bean (5) (Figure 1), which is consistent with no recent WGD event occurred in these two plant species (Chan et al., 2010; Wu et al., 2015). Besides gene expansion, gene loss was also observed in physic nut as seen in *Arabidopsis*. For example, castor bean, rubber tree and poplar harbor three XIP subgroups, whereas physic nut only contains the subgroups XIP1 and XIP2 (Figures 1, 2); castor bean, rubber tree and poplar have two NIP4s that are clustered with their counterparts, respectively, however, physic nut only contains the ortholog of *RcNIP4;2*/*HbNIP4;2*/*PtNIP4;2*; the ortholog of *RcPIP1;2*/*HbPIP1;3* was also lost in physic nut (Figure 1). In addition, compared with physic nut and castor bean, the PIP and TIP subfamilies in rubber tree are shown to be highly expanded (Figure 2).

### Analysis of exon-intron structure

**Figure 3:**
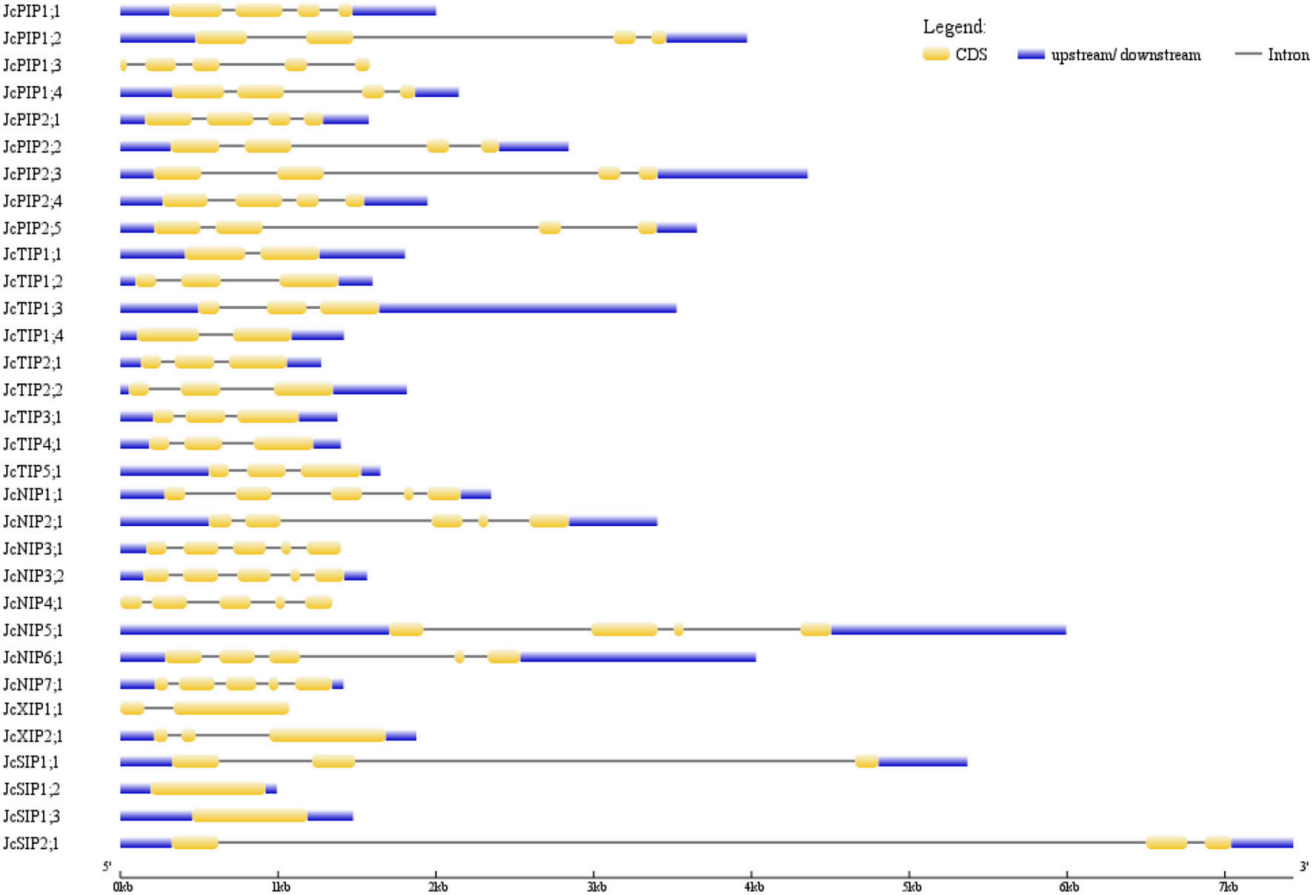
**Exon-intron structures of the 32 JcAQP genes**. The graphic representation of the gene models is displayed using GSDS.

The exon-intron structures of 32 *JcAQPs* were analyzed based on the optimized gene models. Although the ORF (open reading frame) length of each gene is consistent (639–924 bp, similar to 627–830 bp in castor bean, and 684–927 bp in rubber tree), the gene size (from start to stop codons) is distinct (729–6716 bp, longer than 705–4934 bp in castor bean and shorter than 720–13,833 bp in rubber tree) (Table 2 and Figure 3; Zou et al., 2015a,b). The *JcAQP* introns have an average length of about 380 bp (same as that in castor bean but relatively shorter than 404 bp in rubber tree), with the minimum of 63 bp in *JcNIP4;1* (corresponding to 46 bp in *RcPIP2;5* and 71 bp in *HbNIP2;1*) and the maximum of 5879 bp in *JcSIP2;1* (corresponding to 3360 bp in *RcNIP5;1* and 13,000 bp in *HbSIP2;1*) (Figure 3; Zou et al., 2015a,b). Like observed in rubber tree and castor bean (Zou et al., 2015a,b), AQP genes in different subfamilies harbor distinct exon-intron structures. Except for *JcPIP1;3* that contains four introns, other JcPIP subfamily members feature three introns (83–481, 90–1751, and 87–487 bp, respectively). It is worth noting that *JcPIP1;3* is more likely to be a pseudogene, because no evidence is available for its expression and a C deletion at the 82th position and an A/T mutation at the 456th position were observed when compared with other JcPIP1 genes. Most *JcTIPs* contain two introns (75–302 bp and 77–372 bp, respectively), while *JcTIP1;1* and *JcTIP1;4* contain only one intron. Most *JcNIPs* harbor four introns (70–1063, 72–957, 79–980, and 88–262 bp, respectively), whereas *JcNIP5;1* contain three introns instead. Two out of three *JcSIP1s* don't contain introns, in contrast, *JcSIP1;3* and the only JcSIP2 subgroup member *JcSIP2;1* harbor two introns. The two identified JcXIP subfamily members *JcXIP1;1* and *JcXIP2;1* contain one or two introns, respectively (Figure 3).

### Subcellular localization, structural features, and functional inference

Sequence analysis showed that the 32 deduced JcAQPs range from 212 to 307 amino acids, with the theoretical molecular weight of 22.57 to 32.29 kDa and the pI value of 4.96 to 10.02. Homology analysis of these deduced proteins revealed a high sequence diversity existing within and between the five subfamilies. The sequence similarities of 57.6–92.9% were found within JcTIPs, 56.5–94.4% within JcPIPs, 43.5–81.1% within JcSIPs, 42.4–73.3% within JcNIPs, and 49.5% within JcXIPs. JcPIPs share sequence similarities of 32.1–47.9, 29.9–41.0, 24.8–37.8, or 23.4–31.6% with JcTIPs, JcXIPs, JcNIPs, and JcSIPs, respectively. JcTIPs show 30.3–40.9, 29.9–37.3, or 29.5–35.6% sequence similarities with JcNIPs, JcSIPs, and JcXIPs, respectively. JcNIPs share sequence similarities of 29.4–36.7 or 23.9–32.0% with JcXIPs and JcSIPs, respectively, whereas JcSIPs share the lowest sequence similarity of 22.6–27.3% with JcXIPs (Supplementary Table S2). These results indicated that the SIP subfamily has formed an outstanding group to other subfamilies, and the XIP subfamily share a closer evolutionary relationship with the PIP subfamily than with other subfamilies. Despite the overall sequence similarities between different subfamily members are relatively low, topological analyses showed that all JcAQPs were predicted to harbor six transmembrane helical domains, which is consistent with the results from multiple alignments with structure proven AQPs (Table 3 and Supplementary File S5).

**Table 3:** **Structural and subcellular localization analysis of the JcAQPs**.

**Name**	**Len**	**Mw (KDa)**	**pI**	**TM^a^**	**Loc^b^**	**Ar/R selectivity filter**				**NPA motifs**		**Froger's positions**				
						**H2**	**H5**	**LE1**	**LE2**	**LB**	**LE**	**P1**	**P2**	**P3**	**P4**	**P5**
JcPIP1;1	287	30.73	8.60	6	Plas	F	H	T	R	NPA	NPA	E	S	A	F	W
JcPIP1;2	287	30.54	8.62	6	Plas	F	H	T	R	NPA	NPA	E	S	A	F	W
JcPIP1;3	212	22.57	8.53	6	Plas	F	H	T	R	NPA	NPA	E	S	A	F	W
JcPIP1;4	286	30.46	8.72	6	Plas	F	N	A	R	NPA	NPA	Q	S	A	F	W
JcPIP2;1	284	30.35	8.20	6	Plas	F	H	T	R	NPA	NPA	Q	S	A	F	W
JcPIP2;2	286	30.60	7.62	6	Plas	F	H	T	R	NPA	NPA	Q	S	A	F	W
JcPIP2;3	285	30.33	8.67	6	Plas	F	H	T	R	NPA	NPA	Q	S	A	F	W
JcPIP2;4	280	29.91	8.97	6	Plas	F	H	T	R	NPA	NPA	M	S	A	F	W
JcPIP2;5	283	29.67	9.10	6	Plas	F	H	T	R	NPA	NPA	V	S	A	F	W
JcTIP1;1	252	25.97	5.91	6	Plas	H	I	A	V	NPA	NPA	T	S	A	Y	W
JcTIP1;2	252	25.79	4.96	6	Vacu	H	I	A	V	NPA	NPA	T	S	A	Y	W
JcTIP1;3	252	25.79	5.13	6	Vacu	H	I	A	V	NPA	NPA	T	S	A	Y	W
JcTIP1;4	254	26.39	5.83	6	Vacu	H	V	A	V	NPA	NPA	T	S	A	Y	W
JcTIP2;1	248	25.26	5.59	6	Vacu	H	I	G	R	NPA	NPA	T	S	A	Y	W
JcTIP2;2	250	25.34	5.69	6	Vacu	H	I	G	R	NPA	NPA	T	S	A	Y	W
JcTIP3;1	257	27.34	6.49	6	Vacu	H	I	A	R	NPA	NPA	T	A	A	Y	W
JcTIP4;1	247	25.97	6.12	6	Vacu	H	I	A	R	NPA	NPA	T	S	A	Y	W
JcTIP5;1	252	26.01	7.85	6	Vacu	N	V	G	S	NPA	NPA	I	A	A	Y	W
JcNIP1;1	275	29.27	9.21	6	Plas	W	V	A	R	NPA	NPA	F	S	A	Y	L
JcNIP2;1	291	30.80	8.85	6	Plas	G	S	G	R	NPA	NPA	L	T	A	Y	I
JcNIP3;1	277	30.04	8.50	6	Plas	W	V	A	R	NPA	NPA	F	S	A	F	L
JcNIP3;2	280	30.04	5.52	6	Vacu	W	M	A	R	NPA	NPA	F	S	A	Y	I
JcNIP4;1	263	27.82	5.89	6	Plas	W	V	A	R	NPA	NPA	F	S	A	Y	I
JcNIP5;1	298	30.85	8.87	6	Plas	S	I	A	R	NPA	NPV	F	T	A	Y	L
JcNIP6;1	307	31.37	8.71	6	Plas	S	I	A	R	NPS	NPV	L	T	A	Y	L
JcNIP7;1	265	28.17	7.00	6	Plas	A	V	G	R	NPA	NPA	Y	S	A	Y	I
JcXIP1;1	294	32.27	6.05	6	Plas	I	I	V	R	SPI	NPA	M	C	A	F	W
JcXIP2;1	303	32.29	6.59	6	Plas	I	T	V	R	NPV	NPA	L	C	A	F	W
JcSIP1;1	239	25.79	9.58	6	Plas	V	L	P	N	NPT	NPA	I	A	A	Y	W
JcSIP1;2	242	26.01	9.73	6	Vacu	A	L	P	N	NPT	NPA	M	A	A	Y	W
JcSIP1;3	243	26.00	10.02	6	Extr	S	L	P	N	NPT	NPA	M	A	A	Y	W
JcSIP2;1	241	26.37	9.57	6	Vacu	S	L	G	S	NPL	NPA	F	V	A	Y	W

a *Representing the numbers of transmembrane helices predicted by TOPCONS*.

b *Best possible subcellular localization prediction by the WoLF PSORT (Chlo, chloroplast; Cyto, cytosol; ER, endoplasmic reticulum; Plas, plasma membrane; Vacu, vacuolar membrane)*.

As the names suggested, JcPIPs with an average *p*I value of 8.56 and JcTIPs with an average *p*I value of 5.95 were predicted to localize to the plasma membrane or vacuole, respectively, though several PIPs in other plant species were also shown to target the chloroplast membrane (Ferro et al., 2003; Uehlein et al., 2008; Beebo et al., 2013). NIPs were determined to localize to the plasma membrane, endoplasmic reticulum or peribacteroid membrane of root nodules in other organisms (Ma et al., 2006; Mizutani et al., 2006; Takano et al., 2006), whereas our *in silico* predictions indicated that JcNIPs with an average *p*I value of 7.82 are mainly localized to the plasma membrane except for the vacuole prediction of JcNIP3;2. JcSIPs with an average *p*I value of 9.73 were predicted to localize to the plasma membrane and vacuole, however, *Arabdopsis* and grapevine SIPs were shown to be localized to the endoplasmic reticulum (Ishikawa et al., 2005; Noronha et al., 2014). Two JcXIPs with an average *p*I value of 6.32 were predicted to localize to the plasma membrane, which is consistent with the experimental results (Bienert et al., 2011). Nevertheless, thus far, only the plasma membrane localization of JcPIP2;4 and the vacuole localization of JcTIP1;2 have been confirmed by experimental means yet (Khan et al., 2015).

Although AQPs were first identified for their high water permeability, accumulating evidence shows that some of them also transport glycerol, urea, boric acid, silicic acid, NH_3_, CO_2_, H_2_O_2_, etc. Atomic resolution and molecular dynamics stimulations indicated that the ar/R filter, NPA motifs and Froger's positions all affect the substrate specificity: the two opposite NPA motifs create an electrostatic repulsion of protons and act as a size barrier; the ar/R filter renders the pore constriction site diverse in both size and hydrophobicity; the residues at Froger's positions are helpful for discriminating aquaglyceroporins from AQPs, since aquaglyceroporins usually feather an aromatic residue at P1, an acidic residue at P2, a basic residue at P3, a proline followed by a nonaromatic residue at P4 and P5 (Froger et al., 1998; Törnroth-Horsefield et al., 2006). In addition, nine SDPs pivotal for the transport of non-aqua substrates (i.e., urea, boric acid, silicic acid, NH_3_, CO_2_, and H_2_O_2_) were also proposed by Hove and Bhave (2011). To learn more about the putative function of JcAQPs, the residues at these conserved positions were carefully identified based on the multiple alignments with structure/function characterized AQPs (Supplementary File S6). As shown in Table 3, most JcAQPs exhibit an AqpZ-like Froger's positions (A^103^-S^190^-A^194^-F^208^-W^209^) to favor the permeability of water, which is consistent with the high water transport activity of JcPIP2;4 and JcTIP1;2 (Khan et al., 2015). In contrast, JcSIP2;1 and NIP subfamily members possess mixed key residues of GlpF (Y^108^-D^207^-K^211^-P^236^-L^237^) for P1 and P5, and AqpZ for P2–P4. Given the glycerol permease activity of soybean NOD26 and *Arabidopsis* NIPs (Dean et al., 1999; Wallace and Roberts, 2005), JcNIPs are more likely to transport glycerol and may play a role in oil formation/translocation.

Besides high permeability to water, plant PIPs were shown to transport urea, boric acid, CO_2_, and H_2_O_2_ (Eckert et al., 1999; Uehlein et al., 2008; Fitzpatrick and Reid, 2009; Bienert et al., 2014). As shown in Table 3, except for JcPIP1;4 that harbors an ar/R filter of F-N-A-R, all other JcPIPs represent the F-H-T-R ar/R filter as observed in AqpZ (Savage et al., 2003), indicating their high water permeability. According to the SDP analysis, all JcPIPs except for JcPIP1;3 represent urea-type SDPs (H-P-F-F/L-L-P-G-G-N); JcPIP1;1, JcPIP1;2, and JcPIP1;3 represent boric acid-type SDPs (T-I-H-P-E-L-L-T-P); JcPIP1;2 represents CO_2_-type SDPs (L-I-C-A-I-D-W-D-W), whereas JcPIP1;1, JcPIP1;4, JcPIP2;1, JcPIP2;2, and JcPIP2;4 may represent novel SDPs of I/L-M-C-A-I/V-D-W-D-W; all JcPIPs except for JcPIP1;3 represent H_2_O_2_-type SDPs (A-G-V-F/L-I-H/Q-F-V-P) (Table 4 and Supplementary File S6), supporting their similar functionality.

**Table 4:** **Summary of typical SDPs and those identified in the JcAQPs^a^**.

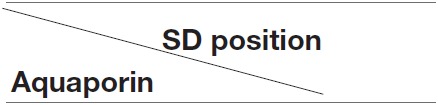	**SDP1**	**SDP2**	**SDP3**	**SDP4**	**SDP5**	**SDP6**	**SDP7**	**SDP8**	**SDP9**
**Typical NH**_3_ **transporter**	**F/T**	**K/L/N/V**	**F/T**	**V/L/T**	**A**	**D/S**	**A/H/L**	**E/P/S**	**A/R/T**
JcNIP1;1	F	K	F	T	A	D	L	E	T
**Typical boric acid transporter**	**T/V**	**I/V**	**H/I**	**P**	**E**	**I/L**	**I/L/T**	**A/T**	**A/G/K/P**
JcPIP1;1	T	I	H	P	E	L	L	T	P
JcPIP1;2	T	I	H	P	E	L	L	T	P
JcPIP1;3	T	I	H	P	E	L	L	T	P
JcNIP2;1	V	V	H	P	E	I	I	A	P
JcNIP5;1	T	I	H	P	E	L	L	A	P
JcNIP6;1	T	I	H	P	E	L	L	A	P
**Typical CO**_2_ **transporter**	**I/L/V**	**I**	**C**	**A**	**I/V**	**D**	**W**	**D**	**W**
JcPIP1;1	I	M	C	A	I	D	W	D	W
JcPIP1;2	L	I	C	A	I	D	W	D	W
JcPIP1;4	I	M	C	A	I	D	W	D	W
JcPIP2;1	I	M	C	A	V	D	W	D	W
JcPIP2;2	L	M	C	A	V	D	W	D	W
JcPIP2;4	L	M	C	A	I	D	W	D	W
**Typical H**_2_**O**_2_ **transporter**	**A/S**	**A/G**	**L/V**	**A/F/L/T/V**	**I/L/V**	**H/I/L/Q**	**F/Y**	**A/V**	**P**
JcPIP1;1	A	G	V	F	I	H	F	V	P
JcPIP1;2	A	G	V	F	I	H	F	V	P
JcPIP1;4	A	G	V	F	I	H	F	V	P
JcPIP2;1	A	G	V	F	I	Q	F	V	P
JcPIP2;2	A	G	V	F	I	Q	F	V	P
JcPIP2;3	A	G	V	F	I	Q	F	V	P
JcPIP2;4	A	G	V	F	I	H	F	V	P
JcPIP2;5	A	G	V	L	I	H	F	V	P
JcTIP1;2	S	A	L	A	I	H	Y	V	P
JcTIP3;1	S	A	L	V	I	H	Y	V	P
JcTIP5;1	S	A	L	A	I	Q	Y	V	P
JcNIP2;1	A	A	L	L	V	I	Y	V	P
JcNIP4;1	S	A	L	L	V	L	Y	A	P
JcNIP5;1	S	A	L	V	V	I	Y	V	P
JcXIP2;1	A	G	L	A	V	H	F	V	P
**Typical silicic acid transporter**	**C/S**	**F/Y**	**A/E/L**	**H/R/Y**	**G**	**K/N/T**	**R**	**E/S/T**	**A/K/P/T**
JcNIP2;1	G	F	V	H	G	N	R	T	K
**Typical urea transporter**	**H**	**P**	**F/I/L/T**	**A/C/F/L**	**L/M**	**A/G/P**	**G/S**	**G/S**	**N**
JcPIP1;1	H	P	F	F	L	P	G	G	N
JcPIP1;2	H	P	F	F	L	P	G	G	N
JcPIP1;4	H	P	F	F	L	P	G	G	N
JcPIP2;1	H	P	F	F	L	P	G	G	N
JcPIP2;2	H	P	F	F	L	P	G	G	N
JcPIP2;3	H	P	F	F	L	P	G	G	N
JcPIP2;4	H	P	F	F	L	P	G	G	N
JcPIP2;5	H	P	F	L	L	P	G	G	N
JcTIP1;1	H	P	F	F	L	A	G	S	N
JcTIP1;2	H	P	F	F	L	A	G	S	N
JcTIP1;3	H	P	F	F	L	A	G	S	N
JcTIP1;4	H	P	F	F	L	A	G	S	N
JcTIP2;1	H	P	F	A	L	P	G	S	N
JcTIP2;2	H	P	F	A	L	P	G	S	N
JcTIP4;1	H	P	L	L	L	A	G	S	N
JcTIP5;1	H	P	F	A	L	P	G	S	N
JcNIP1;1	H	P	L	A	L	P	G	S	N
JcNIP2;1	H	P	T	A	M	P	G	S	N
JcNIP3;1	H	P	I	A	L	P	G	S	N
JcNIP4;1	H	P	I	A	L	P	G	S	N
JcNIP5;1	H	P	I	A	L	P	G	S	N
JcNIP6;1	H	P	I	A	L	P	G	S	N

a *The SDP residues in the physic nut AQPs differing from the typical SDPs determined in this study are highlighted in red*.

Although highly variable in the ar/R filter, plant TIPs were shown to transport water as efficiently as PIPs. Additionally, they also allow urea, NH_3_ and H_2_O_2_ through (Dynowski et al., 2008a,b). As shown in Table 4 and Supplementary File S6, all JcTIPs except for JcTIP3;1 represent urea-type SDPs (H-P-F/L-A/F/L-L-A/P-G-S-N); JcTIP1;2, JcTIP3;1, and JcTIP5;1 represent H_2_O_2_-type SDPs (S-A-L-A/V-I-H/Q-Y-V-P), indicating similar functionality.

In addition to glycerol and water, plant NIPs have been found to transport urea, boric acid, silicic acid, NH_3_, and H_2_O_2_ (Ma et al., 2006; Dynowski et al., 2008a,b). As shown in Table 4 and Supplementary File S6, JcNIP1;1 is promised to be an NH_3_ and urea transporter with nine SDPs of F-K-F-T-A-D-L-E-T or H-P-L-A-L-P-G-S-N, respectively; JcNIP3;1 is promised to be a urea transporter with SDPs of H-P-I-A-L-P-G-S-N; JcNIP4;1 is promised to be an H_2_O_2_ and urea transporter with SDPs of S-A-L-L-V-L-Y-A-P or H-P-I-A-L-P-G-S-N, respectively; JcNIP5;1 is promised to be a boric acid, H_2_O_2_ and urea transporter with SDPs of T-I-H-P-E-L-L-A-P, S-A-L-V-V-I-Y-V-P or H-P-I-A-L-P-G-S-N, respectively; JcNIP6;1 is promised to be a boric acid and urea transporter with SDPs of T-I-H-P-E-L-L-A-P or H-P-I-A-L-P-G-S-N, respectively; JcNIP2;1 represent typical boric acid SDPs (V-V-H-P-E-I-I-A-P), NH_3_ SDPs (A-A-L-L-V-I-Y-V-P), and urea SDPs (H-P-T-A-M-P-G-S-N), however, whether it represents a novel silicic acid SDPs (G-F-V-H-G-N-R-T-K with the substitution of G for C/S at SDP1 and V for A/E/L at SDP3) needs to be experimentally validated. Nevertheless, JcNIP2;1 possesses a distance of 108 amino acids between two NPA motifs, which was shown to be a feature specific to silicon transporters (Deshmukh et al., 2015).

According to phylogenetic relationships, the newly identified XIPs can be divided into five subgroups (XIP1–5) and XIP1–3 were found in poplar, castor bean and rubber tree (Lopez et al., 2012; Zou et al., 2015a,b). Functional analysis indicated that XIPs are able to transport water, glycerol, urea, boric acid, and H_2_O_2_ (Bienert et al., 2011; Lopez et al., 2012). The physic nut harbors one XIP1 and one XIP2. Exhibiting an AqpZ-like Froger's positions, two JcXIPs are more likely to transport water. In addition, JcXIP2;1 is promised to be an H_2_O_2_ transporter with SDPs of A-G-L-V-L-H-F-V-P (Table 4 and Supplementary File S6).

### Tissue-specific expression profiles of JcAQP genes

**Figure 4:**
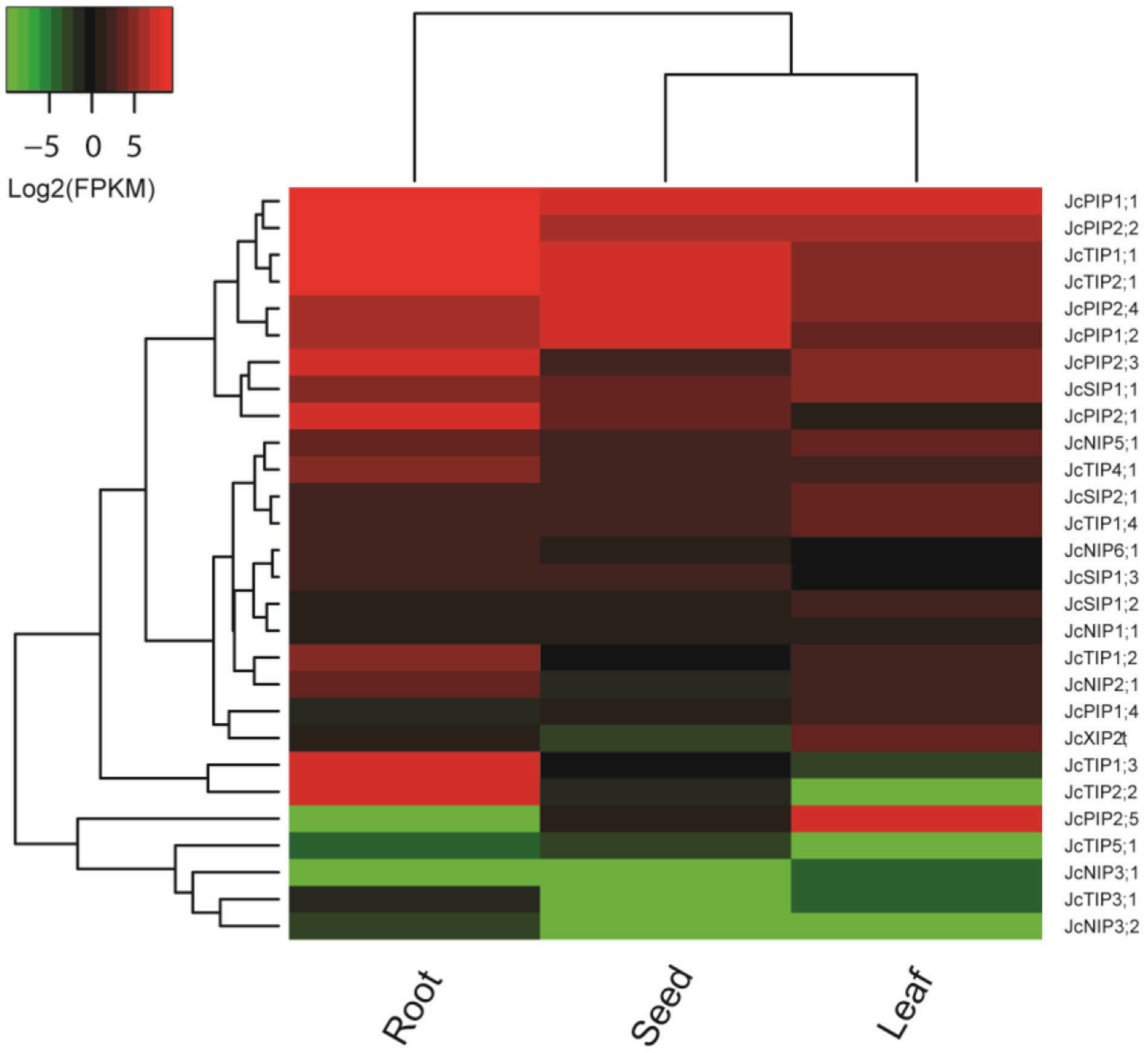
**Expression profiles of the 28 JcAQP genes in roots, leaves and seeds**. Color scale represents FPKM normalized log_2_ transformed counts where green indicates low expression and red indicates high expression.

As a part of the genome sequencing, the transcriptomes of three important tissues (i.e., root, leaf, and seed) of cultivar GZQX0401 were also deeply sequenced (all counting about 30 M 75-nt paired-end reads): roots were collected from 15-day old seedlings, whereas half expanded leaves and seeds from fruits harvested 19–28 DAP (days after pollination) were obtained from 4-year-old plants (Wu et al., 2015). Expression profiling indicated that, except for *JcPIP1;3, JcNIP4;1, JcNIP7;1*, and *JcXIP1;1*, other 28 JcAQP genes were all detected in one of the examined tissues (Figure 4). In contrast to *JcPIP1;3* and *JcXIP1;1* that the expression was not supported by currently available transcriptome data, *JcNIP7;1* was detected in roots after 24 h waterlogging stress (Juntawong et al., 2014), whereas *JcNIP4;1* was shown to be expressed in flowers as supported by one EST (GW619951), which is consistent with the flower-specific expression of its ortholog in castor bean (*RcNIP4;1*) (Zou et al., 2015b). According to the FPKM annotation, the JcAQP genes were shown to be expressed most in roots, which exhibited about 2.95 and 6.30 folds than that in seeds and leaves. PIPs represented the most abundant subfamily in all examined tissues, followed by TIPs, SIPs, NIPs, and XIPs: in roots, the total expression level of PIP members was about 1.55, 58.23, 74.51, and 2707.81 folds more than the TIP, SIP, NIP and XIP members, respectively; 1.42, 33.17, 105.01, and 17,702.00 folds in seeds, respectively; and 7.45, 13.85, 23.26, and 54.58 folds in leaves, respectively (Figure 4), indicating a crucial role of the PIP subfamily in the water balance of these tissues. In tissues such as roots and seeds with a large central vacuole, the plasma membrane-located JcPIPs facilitate the water transport from the extracellular space to the cell cytoplasm, whereas the vacuole-targeted JcTIPs play an essential role in maintaining the cell osmotic balance (Hunter et al., 2007). In contrast, in immature tissues characterized by polydispersed microvacuoles, the role of JcTIPs is less important. Indeed, as shown in Figure 4, the total TIP transcript level in half expanded leaves was considerably lower than that in roots and seeds, only counting about 4.37 or 12.28%, respectively; by contrast, the PIP transcripts counted 20.96 or 64.31%, respectively.

Different JcAQP members exhibited distinct expression profiles in a certain tissue. In roots, two most highly abundant PIP members (*JcPIP1;1* and *JcPIP2;1*) occupied 71.15% of the total PIP transcripts; *JcTIP1;1* and *JcTIP2;1* counted 76.13% of the total TIP transcripts; *JcNIP5;1* and *JcNIP2;1* counted 78.42% of the total NIP transcripts; *JcSIP1;1* counted 70.73% of the total SIP transcripts. In seeds, *JcPIP2;4* and *JcPIP1;1* counted 69.36% of the total PIP transcripts; *JcTIP1;1* and *JcTIP2;1* counted 98.59% of the total TIP transcripts; *JcNIP5;1, JcNIP6;1*, and *JcNIP1;1* counted 98.56% of the total NIP transcripts; *JcSIP1;1* counted 71.48% of the total SIP transcripts. In leaves, *JcPIP2;5* and *JcPIP1;1* counted 74.51% of the total PIP transcripts; *JcTIP1;1* and *JcTIP2;1* counted 76.86% of the total TIP transcripts; *JcNIP5;1* counted 69.20% of the total NIP transcripts; *JcSIP1;1* and *JcSIP2;1* counted 91.27% of the total SIP transcripts. Except for the JcXIP subfamily, more than one subfamily members were detected in a certain tissue. Compared with roots and seeds, *JcXIP2;1* was shown to be expressed considerably higher in leaves. Among 26 JcAQP genes detected in roots (excluding *JcPIP2;5* and *JcNIP3;1*), *JcPIP1;1, JcTIP1;1, JcNIP5;1*, and *JcSIP1;1* represented the most abundant PIP, TIP, NIP, and SIP subfamily members, respectively. Except for *JcTIP3;1, JcNIP3;1*, and *JcNIP3;2*, other 25 members were shown to be expressed in seeds and *JcPIP2;4, JcTIP1;1, JcNIP5;1*, and *JcSIP1;1* represented the most abundant PIP, TIP, NIP, and SIP subfamily members, respectively. In leaves, 25 JcAQP genes (excluding *JcTIP2;2, JcTIP5;1*, and *JcNIP3;2*) were detected and *JcPIP2;5, JcTIP1;1, JcNIP5;1*, and *JcSIP1;1* represented the most abundant PIP, TIP, NIP, and SIP subfamily members, respectively. In contrast to the highly abundant and constitutive expression of *JcPIP1;1, JcPIP2;2, JcTIP1;1* and *JcTIP2;1, JcPIP1;2* and *JcPIP2;4* preferred to express in roots and seeds; *JcPIP2;1, JcPIP2;3, JcTIP1;2, JcTIP1;3*, and *JcTIP2;2* preferred to express in roots; *JcPIP2;5* preferred to express in leaves. In addition, *JcNIP3;1* and *JcNIP3;2*, two orthologs of castor bean *RcNIP3;1*, exhibited an organ-specific expression pattern. *RcNIP3;1* was shown to be expressed in leaves but not in seeds (Zou et al., 2015b), whereas *JcNIP3;1* and *JcNIP3;2* was expressed only in leaves and roots, respectively (Figure 4).

### Tissue-specific expression profiles of HbAQP genes

**Figure 5:**
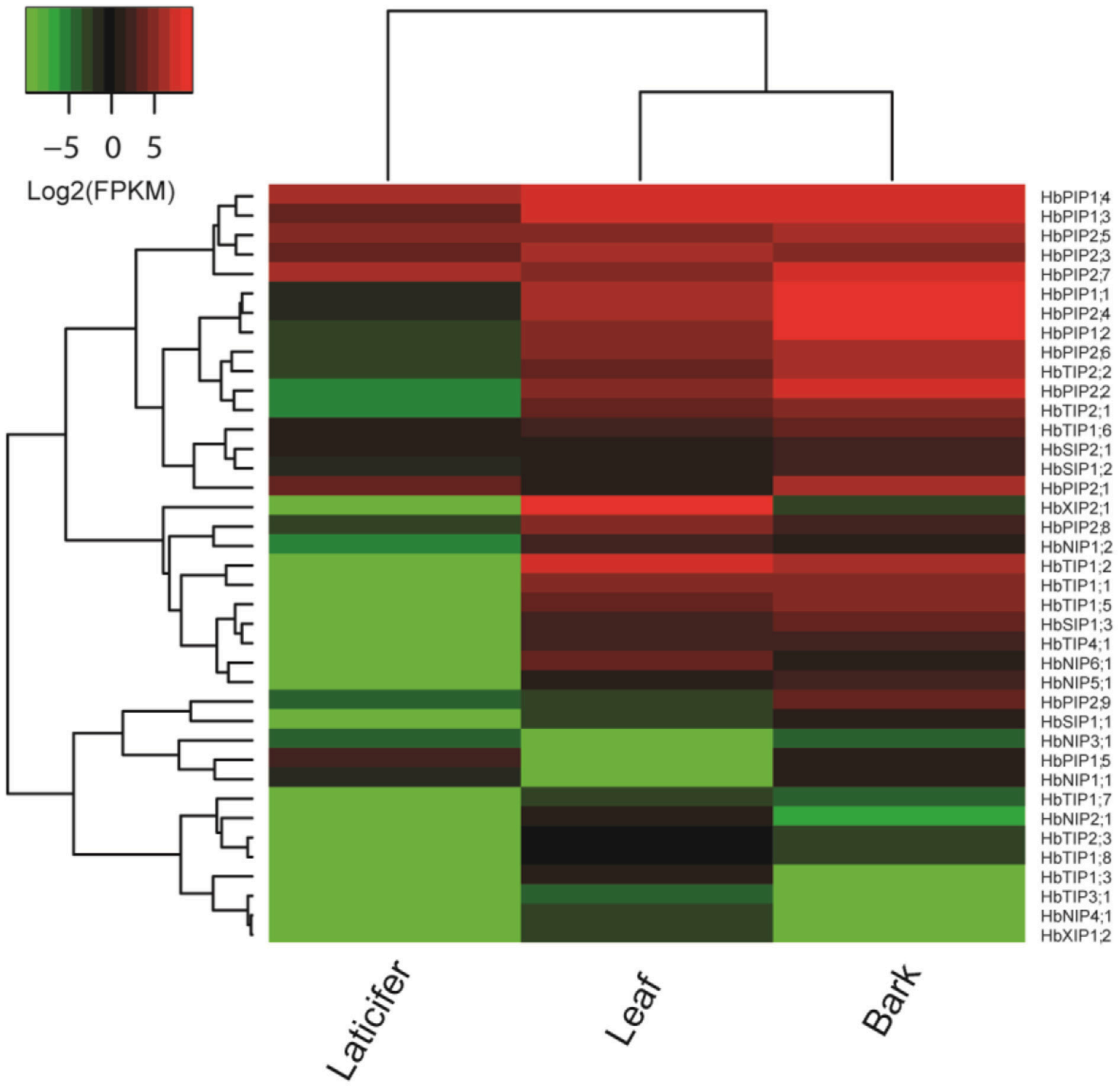
**Expression profiles of the 39 HbAQP genes in laticifers, barks and leaves**. Color scale represents FPKM normalized log_2_ transformed counts where green indicates low expression and red indicates high expression.

In the previous study, we reported the identification of 51 AQP genes from rubber tree genome, and focused on their response to ethephon stimulation in the rubber-producing tissue termed laticifer (Zou et al., 2015a) which is not found in physic nut and castor bean. To gain insights into the expression evolution of duplicated HbAQP genes, in the present study, we take advantage of deep transcriptome sequencing data to investigate their expression profiles in two more important tissues, i.e., bark and leaf (all counting about 25 M 100-nt paired-end reads). As shown in Figure 5, 39 out of 51 HbAQP genes representing all five subfamilies were detected in at least one of the examined tissues, though the expression of the XIP subfamily members was not observed in the laticifer. FPKM annotation indicated HbAQP genes were expressed most in barks, exhibiting 1.79 and 11.49 folds more than that in leaves and laticifers, respectively. As observed in castor bean and physic nut, PIPs represented the most abundant subfamily in all examined tissues: in barks, the total expression level of PIP members was 8.77, 95.51, 235.92, and 12,090.95 folds more than the TIP, SIP, NIP, or XIP members, respectively; 1.20, 2.70, 36.52, and 80.52 folds more than the XIP, TIP, NIP, or SIP members in leaves, respectively; and 116.11, 122.84, and 752.87 folds more than the SIP, TIP, or NIP members in laticifers, respectively (Figure 5). Nevertheless, compared with laticiferous cells that are characterized by a high number of polydispersed microvacuoles (Wang X. C. et al., 2013), cells of bark and mature leaf usually contain a large central vacuole and the role of HbPIPs is less important. Instead, the total TIP transcripts in barks and leaves were 147.25 and 135.28 folds more than that in laticifers, respectively. Compared with the PIPs, TIPs, and SIPs expressed more in barks, NIPs and XIPs were shown to be expressed more in leaves (Figure 5). It is worth noting that the expression level of *HbXIP2;1* was particularly high in leaves, counting 99.98% of the total XIP transcripts. Similar expression pattern was also observed in physic nut and castor bean, where its orthologs *JcXIP2;1* and *RcXIP2;1* were shown to be preferentially expressed in leaves (Figure 4; Zou et al., 2015b). Compared with laticifers where three highly abundant PIP members (i.e., *HbPIP2;7, HbPIP1;4*, and *HbPIP2;5*) occupied 80.99% of the total PIP transcripts, seven PIPs (i.e., *HbPIP1;2, HbPIP1;1, HbPIP2;4, HbPIP1;4, HbPIP2;7, HbPIP1;3*, and *HbPIP2;2*) occupied 89.53% of the total PIP transcripts in barks, and seven abundant PIPs (i.e., *HbPIP1;4, HbPIP1;3, HbPIP2;3, HbPIP2;4, HbPIP1;1, HbPIP2;6*, and *HbPIP1;2*) occupied 83.57% of the total PIP transcripts in leaves. In barks, *HbTIP1;2, HbTIP2;2*, and *HbTIP2;1* counted 73.04% of the total TIP transcripts; *HbNIP5;1* and *HbNIP1;2* counted 67.31% of the total NIP transcripts; *HbSIP1;3* counted 56.32% of the total SIP transcripts. In leaves, *HbTIP1;2*, and *HbTIP1;1* counted 81.44% of the total TIP transcripts; *HbNIP6;1, HbNIP1;2*, and *HbNIP5;1* counted 92.39% of the total NIP transcripts; *HbSIP1;3* and *HbSIP2;1* counted 78.49% of the total SIP transcripts. Among 35 HbAQP genes detected in barks, *HbPIP1;2, HbTIP1;2, HbNIP5;1, HbXIP2;1*, and *HbSIP1;3* represented the most abundant PIP, TIP, NIP, XIP, and SIP subfamily members, respectively. Among 36 HbAQP genes detected in leaves, *HbPIP1;4, HbTIP1;2, HbNIP6;1, HbXIP2;1*, and *HbSIP1;3* represented the most abundant PIP, TIP, NIP, XIP, and SIP subfamily members, respectively (Figure 5).

## Discussion

Gene duplication is a major mechanism for acquiring new genes and creating genetic novelty in eukaryotes. Gene duplicates may originate from single gene duplications such as local (tandem or proximal), dispersed and transposed duplications, or large-scale duplications such as WGDs and segmental duplications (Wang et al., 2012). WGDs are widespread and play an important role in the origin and diversification of the angiosperms (Bowers et al., 2003). According to the comparative genomics analysis, all core eudicot plant species including *Arabidopsis*, poplar, rubber tree, castor bean, and physic nut underwent one so-called γ whole-genome triplication event occurred at about 117 million years ago (Mya) (Jiao et al., 2012). Moreover, it is well established that poplar and *Arabidopsis* underwent one or two recent doubling events, respectively (Bowers et al., 2003; Tuskan et al., 2006). The doubling event occurred in poplar was shown to be specific to Salicaceae date to about 60–65 Mya (Tuskan et al., 2006), whereas the β and α WGDs occurred in *Arabidopsis* are Brassicaceae-specific and not distantly separated, probably date to 20–40 Mya (Blanc and Wolfe, 2004). By contrast, like castor bean, physic nut didn't undergo any recent WGD (Chan et al., 2010; Wu et al., 2015). From this perspective, the recently available physic nut genome may provide a good chance to analyze the lineage-specific expansion and evolution of certain gene families in Euphorbiaceus.

### Physic nut encodes fewer AQP genes than other plants including rubber tree

Our genome-wide survey indicated that physic nut encodes 32 AQP genes, and 30 out of them were shown to be expressed. To our knowledge, this family number is the fewest in high plants reported to date (Table 1 and references as in). Nevertheless, physic nut contains members representing all five subfamilies (i.e., PIP, TIP, NIP, XIP, and SIP) found in high plants. In contrast, monocot, and Brassicaceae plants are shown to have lost the XIP subfamily (Table 1 and references as in).

The phylogenetic analysis further divided the JcAQP subfamilies into subgroups. Except for the XIP subfamily, the classification is shown to be the same as that in castor bean, rubber tree, and poplar (Zou et al., 2015a,b). The XIPs in the above three Malpighiales plants can be divided into three subgroups, which is supported by the sequence similarity and the ar/R filter (Zou et al., 2015a,b). By contrast, physic nut only retains the XIP1 and XIP2 subgroups.

In addition to gene loss, the comparative analysis also revealed the expansion of specific JcAQP genes, i.e., two gene pairs (*JcNIP3;1*/*JcNIP3;2* and *JcSIP1;2*/*JcSIP1;3*) as shown in Figure 1. *JcNIP3;1*/*JcNIP3;2* can be defined as dispersed duplicate genes for their distribution on two different chromosomes, whereas *JcSIP1;2*/*JcSIP1;3* as well as five RcAQP gene pairs can be classed as tandem duplicates since they are characterized by same-direction neighbors (foot-to-head order) on the same scaffolds. Thereby, tandem duplications act as the main force for the expansion of AQP genes in physic nut and castor bean. By contrast, in *Arabidopsis*, poplar and rubber tree, WGDs seem to play a more important role in the family expansion. For example, studies showed that the 35 AtAQP genes are more likely to be derived from 17 parents, including 9, 3, and 1 genes resulted from α, β, or γ WGDs, respectively (Wang Y. et al., 2013).

Consistent with that all HbAQPs have orthologs in poplar (Zou et al., 2015a), all Jc/RcAQPs are shown to have orthologs in rubber tree and poplar (Table 1 and Zou et al., 2015b). By using poplar as an outgroup, we estimate that there are 31 AQP family members in the ancestral Euphorbiaceae species, including 3 PIP1s, 5 PIP2s, 4 TIP1s, 2 TIP2s, 1 TIP3, 1 TIP4, 1 TIP5, 1 NIP1, 1 NIP2, 1 NIP3, 2 NIP4, 1 NIP5, 1 NIP6, 1 NIP7, 1 XIP1, 1 XIP2, 1 XIP3, 2 SIP1s, and 1 SIP2. After a round of a recent WGD and subsequent chromosomal rearrangement, rubber tree preferred to retain the genes of the PIP (especially the PIP2 subgroup) and TIP (especially the subgroups TIP1, TIP2, TIP3, and TIP5) subfamilies, corresponding to their high water permeability (e.g., HbPIP2;1, HbPIP2;5, and HbTIP1;1), the particular importance of water balance in a big tree, and a highly differentiated laticifer tissue which is tapped for the cytoplasm in the form of aqueous latex (Tungngoen et al., 2009; An et al., 2015; Zou et al., 2015a). In fact, as a big tree, poplar implemented a very similar strategy, though relatively more NIP5s, NIP6s, and XIP3s have been preserved (Figure 2).

Since the majority of Jc/Rc/HbAQP sequences were confirmed with available cDNAs, ESTs, and/or RNA sequencing read (Table 1 and Zou et al., 2015a,b), we are allowed to investigate the structural divergence of AQP genes in these plant species. As shown in Supplementary Table S3, the comparative analysis indicated that most orthologous genes exhibit the same exon-intron structures. However, *JcPIP1;3* and *RcPIP2;5* harbor four introns instead of the usual three, and *RcXIP1;3* seems to have lost the intron shared by its orthologs and paralogs. In addition, *JcPIP1;3, HbPIP1;5, RcPIP2;5, HbTIP1;4, JcNIP7;1*, and *RcXIP1;4* encode relatively fewer amino acids than their orthologs (Supplementary Table S3), suggesting the occurrence of insertions/deletions in their coding-regions as well as the usual nucleotide substitution. In fact, the comparative analysis revealed that nucleotide substitution have played an important role on the diversification of conserved residues that determine the substrate specificity, and the situation is particularly universal in members of NIP, XIP, and SIP subfamilies (Supplementary Table S4), reflecting a variety of their substrate transport capacity.

### Expression divergence of duplicated HbAQP genes

In addition to structural divergence, expression divergence also plays a key role in the evolution of duplicate genes. Microarray has been frequently used to study the gene expression evolution in model species such as *Arabidopsis* and rice (Blanc and Wolfe, 2004; Li et al., 2009). With the development of the second generation sequencing technologies, RNA sequencing provides an alternative method for such studies (Harikrishnan et al., 2015). Based on the transcriptional profiling of Hb/Jc/RcAQP genes in several important tissues, the expression evolution patterns of duplicated HbAQP genes are discussed as follows.

Among 17 HbAQP gene pairs, *HbPIP1;1*/*HbPIP1;2* exhibited similar expression profiles in all tissues examined, which is consistent with that of *RcPIP1;2*/*RcPIP1;3*, their orthologs in castor bean (Figure 5; Zou et al., 2015b). Nevertheless, the expression levels of *HbPIP1;1* and *HbPIP1;2* were extremely low in laticifers (Figure 5), though they were highly abundant in barks and leaves and their orthologs in physic nut (*JcPIP1;1*) and castor bean were also constitutively expressed in tested tissues (Figure 4; Zou et al., 2015b). *HbPIP1;3* and *HbPIP1;4* showed similar expression profiles in barks and leaves and their high abundance was also similar to that of *JcPIP1;2* and *RcPIP1;4*, their orthologs in physic nut and castor bean, respectively (Figures 4, 5; Zou et al., 2015b). In contrast, the transcript level of *HbPIP1;4* was relatively higher than that of *HbPIP1;3* in laticifers (Figure 5). *HbPIP2;5*/*HbPIP2;6* exhibited similar evolution pattern to that of *HbPIP1;3*/*HbPIP1;4*, and *HbPIP2;5* was expressed considerably more than that of *HbPIP2;6* in laticifers (Figure 5). Although both *HbPIP2;1* and *HbPIP2;2* were highly abundant in barks, *HbPIP2;1* preferred to express in laticifers whereas *HbPIP2;2* preferred to express in leaves (Figure 5). The moderate expression of *HbPIP2;2* in leaves was similar to that of their orthologs in physic nut and castor bean, i.e., *JcPIP2;1* and *RcPIP2;1*, respectively (Figures 4, 5; Zou et al., 2015b). Like the high abundance of their orthologs in physic nut (*JcPIP2;2*) and castor bean (*RcPIP2;3*) in leaves (Figure 4; Zou et al., 2015b), both *HbPIP2;3* and *HbPIP2;4* were highly expressed in rubber tree leaves, however, *HbPIP2;3* was preferentially expressed in laticifers while *HbPIP2;4* was preferentially expressed in barks (Figure 5). Similar to *HbPIP2;3*/*HbPIP2;4, HbPIP2;7*/*HbPIP2;8* were highly abundant in leaves as their orthologs in physic nut (*JcPIP2;4*) and castor bean (*RcPIP2;4*), however, the transcript level of *HbPIP2;7* was considerably higher than that of *HbPIP2;8* in both barks and laticifers (Figure 5). Like the high abundance of *RcTIP1;1* and *JcTIP1;1* in various tissues tested, *HbTIP1;1*/*HbTIP1;2* were highly expressed in rubber tree barks and leaves, though the transcript level of *HbTIP1;2* was relatively higher than that of *HbTIP1;1* (Figure 5). Nevertheless, their expression was not detected in laticifers. In contrast to *HbTIP1;4* whose expression was not detected in all tissues tested, *HbTIP1;3* was only lowly expressed in rubber tree leaves (Figure 5). However, their orthologs in physic nut (*JcTIP1;4*) and castor bean (*RcTIP1;4*) were shown to be expressed in all tested tissues and the transcript levels in leaves were relatively high (Figure 4; Zou et al., 2015b). Similar to *JcTIP1;2* and *RcTIP1;2* (Figure 4; Zou et al., 2015b), both *HbTIP1;5* and *HbTIP1;6* were moderately expressed in leaves as well as in barks, though the transcript level of *HbTIP1;5* was relatively higher than that of *HbTIP1;6*. In addition, the expression of *HbTIP1;5* was not detected in laticifers, whereas *HbTIP1;6* represented the most abundant TIP member (Figure 5). Similar to *JcTIP1;3* and *RcTIP1;3* (Figure 4; Zou et al., 2015b), both *HbTIP1;7* and *HbTIP1;8* were lowly expressed in leaves as well as in barks. Nevertheless, the transcript level of *HbTIP1;8* was considerably higher than that of *HbTIP1;7* (Figure 5). Similar to *JcTIP2;1* and *RcTIP2;1* (Figure 4; Zou et al., 2015b), both *HbTIP2;1* and *HbTIP2;2* represented two of the most abundant TIP members in leaves as well as in barks. However, the transcript level of *HbTIP2;2* was shown to be relatively higher than that of *HbTIP2;1* in both barks and laticifers, though their expression levels in laticifers were extremely low (Figure 5). In contrast to *HbTIP2;4* whose expression was not detected in all tissues tested, *HbTIP2;3* was only lowly expressed in rubber tree leaves and barks (Figure 5). Similar expression profiles were also observed in physic nut and castor bean, their orthologs *JcTIP2;2* and *RcTIP2;2* were lowly expressed in all tested tissues except for physic nut roots (Figure 4; Zou et al., 2015b). *HbTIP3;1/HbTIP3;2* exhibited similar evolution pattern to that of *HbTIP2;3*/*HbTIP2;4* and the only detected *HbTIP3;1* was shown to be lowly expressed in leaves (Figure 5). Except for *RcTIP3;1* that was highly abundant in endosperms, the low expression of their orthologs in physic nut (*JcTIP3;1*) and castor bean was also observed in tested tissues (Figure 4; Zou et al., 2015b). The expression of both *HbTIP5;1* and *HbTIP5;2* was not detected in all tested tissues (Figure 5). In contrast, their orthologs in physic nut (*JcTIP5;1*) and castor bean (*RcTIP5;1*) were shown to be lowly expressed in tested tissues except for physic nut leaves (Figure 4; Zou et al., 2015b). Similar to *JcNIP1;1* and *RcNIP1;1* (Figure 4; Zou et al., 2015b), in leaves, *HbNIP1;2* was shown to be moderately expressed, whereas the expression of *HbNIP1;1* was not detected. In addition, *HbNIP1;1* preferred to express in laticifers whereas *HbNIP1;2* preferred to express in barks (Figure 5). *HbSIP1;2*/*HbSIP1;3* exhibited similar evolution pattern to that of *HbNIP1;1*/*HbNIP1;2*, where *HbSIP1;2* was shown to be expressed more in laticifers and *HbSIP1;3* was expressed more in barks and leaves. In contrast to the high abundance of *RcXIP1;1* in castor bean leaves (Zou et al., 2015b), the expression of both *HbXIP1;3* and *HbXIP1;4* was not detected in all tested tissues (Figure 5).

## Conclusions

Our paper presents the first genome-wide study of the physic nut AQP gene family and using systematic nomenclature assigned 32 JcAQPs into five subfamilies. Furthermore, their structural and functional properties were investigated and the global expression profiles of 32 *JcAQPs* and 51 *HbAQPs* were examined with deep transcriptome sequencing data, which provides insights into the evolution of the duplicated HbAQP genes. Results obtained from this study not only provide valuable information for future functional analysis and utilization of Jc/HbAQP genes, but also provide a useful reference to survey the gene family expansion and evolution in Euphorbiaceus plants and other plant species.

## Author contributions

The study was conceived and directed by ZZ. All the experiments and analysis were directed by ZZ and carried out by ZZ, LY, JG, YM, JW, JC, and FA. ZZ and GX wrote the paper. All the authors read and approved the final manuscript.

### Conflict of interest statement

The authors declare that the research was conducted in the absence of any commercial or financial relationships that could be construed as a potential conflict of interest.
