# Supplementary material for: Genome-Wide Identification of Jatropha curcas Aquaporin Genes and the Comparative Analysis Provides Insights into the Gene Family Expansion and Evolution in Hevea brasiliensis
Source: Front Plant Sci. 2016 Mar 31;7:395. doi: 10.3389/fpls.2016.00395 (PMC4814485; doi:10.3389/fpls.2016.00395)
Supplement: Supplementary file 5 [file Image1.PDF]

Supplementary File S1 The gene model for *JcSIP1;1*. The coding region is marked with uppercase letters, above which is its deduced amino acids. The transcribed untranslated regions, including 5' UTR, intron and 3' UTR sequences, are marked with lowercase letters. The start and stop codons are marked with bold letters.

```
   1 agattagagatccctaattgctaatggaatacaaacccaaaagcttatctaaattagcag
  61 ccaccaaactgccaaacagagctcttctggtagttaggtcaatcacaatcactccatttc
 121 tctgtacattttattttggtacttcaattccaacttgggaacccaaaaaacaacccaatt
 181 ttaaacgtctgctttcctatttcctcccaaaatctcagaccaaccaatcaacaataaagt
 241 tgcttcttttacttcataccagttgctgcctttgctcatacttaaatatacaatcactat
   1                                 M  G  A  V  K  A  A  I  G  D  G
 301 tcactatcaaactatagcaggaattagtgATGGGGGCAGTGAAGGCGGCGATTGGTGATG
  12  V  L  T  F  M  W  V  F  C  S  S  M  F  G  L  F  T  S  L  I
 361 GAGTGCTCACTTTCATGTGGGTTTTCTGTTCTTCAATGTTTGGATTGTTCACTAGCCTCA
  32  A  A  A  L  R  V  Q  N  Y  L  W  A  T  L  F  I  T  T  V  L
 421 TAGCCGCTGCTCTTCGGGTTCAAAATTATTTGTGGGCTACTCTGTTTATCACTACCGTTC
  52  V  F  V  F  V  Y  L  F  G  L  I  A  G  F  L  G  G  A  S  F
 481 TTGTTTTCGTGTTTGTTTACTTGTTTGGCTTGATCGCTGGGTTCTTGGGTGGAGCCAGTT
  72  N  P  T  G  N  A  A  F  Y  A  A  G  V  G  G  D  N  L  F  S
 541 TTAACCCTACTGGTAATGCCGCCTTTTATGCTGCTGGAGTTGGTGGGGATAATCTTTTTT
  92  L  A  L  R  F  P  A  Q
 601 CTTTGGCTCTTAGATTCCCTGCTCAGgtgacattcattatcgtataaaacatatctttct
 661 ttatatatttgcatataggtttagtatatactaatagctgaattcgaattgtcctgaatc
 721 tttggctcttagattccctgctcaggtgatattttgatttcttaatttttatgtgcttgt
 781 ttatattgtttctggtagagagaacataaattggagtgaattttactttctttattttta
 841 gtatcttccgtttttgggtcaaatcacctttttttttttttttgggggggggggggggg
 901 gttttttttttgtttggtgggaggggggggggaatttcttaaagtctctaattgttttt
 961 tctagcaacaggaacagaacatattataattgttgcctgtttttgtgtattgttgttgaa
1021 aattaaatggaagaatatagcttttatagtgagccatgttccagaaaagagaaagggtaa
1081 ttcagagacggtgatagatgtacaagaacttgtttctccaaaaagacagcaactttaaag
1141 gagcgacttagatttataataataacaaacaatatgcttaagaactcgtattaagcatgt
 100                   A  A  G  A  V  G  G  V  L  A  I  L  E  V  I
1201 aataccttcttgacagGCAGCAGGTGCTGTGGGTGGGGTACTGGCTATCTTGGAGGTGAT
 115  P  P  Q  Y  K  H  M  L  G  G  P  S  L  K  V  D  L  H  T  G
1261 ACCACCACAGTATAAGCACATGCTTGGAGGTCCTTCGCTGAAAGTTGACTTGCATACTGG
 135  A  I  A  E  G  V  L  T  F  I  I  T  F  V  V  L  L  I  V  L
1321 AGCCATTGCCGAGGGAGTGTTGACCTTTATAATTACCTTTGTTGTTCTGTTAATTGTCCT
 155  R  G  P  R  N  S  F  V  Q  N  W  L  I  A  V  A  T  V  T  L
1381 TAGAGGTCCTCGCAACTCATTTGTGCAGAATTGGCTGATTGCAGTTGCAACTGTAACATT
 175  V  V  T  G  S  K  Y  T  G  P  S  M  N  P  A  N
1441 GGTTGTCACAGGTTCCAAATACACAGGCCCTTCTATGAACCCTGCTAATgtgagtagtcc
1501 agctttctttacatgttccagattcatttttttgcttttctttttatgtcttaaatatgtg
1561 ctatggtttaaatgatgtgtcaattttgcccctttagtttctaataagaagcagaaattc
```

1621 ctttaaattataagcttattaaatgctttcaaggatttgttgctgtattttgtataatat
1681 gcaattaggtactggttttatttgtgttgcatcttgtcagttttgagtgtaactgtacac
1741 ccagctgctctgactttcttgcaatcaccctttttgttgctactctatccaactctcaaa
1801 agttctgatcactttctttttcattattccatctgatttttttttcctattctcagagtct
1861 tacattatggattatatttatattatttgtttgaagatgtgtaatacactgctttaacat
1921 ttatatgaaatcattgaagatgattttcacatactgtctcatcacactaatatgttatat
1981 cgattggaactggtacctcttcaatttgctttagttttgttcttatgtccctttaacatt
2041 ctagctcttgtgctctttctttatttgcgcatttattgacattgcattgctgagttgcag
2101 atgtttagacataaggttgcaaacatgcttggtcagtctattctctgtccgttctattgg
2161 catgtggctagtttgcctttattcatattatgtgatatgtttattgagtcatccatttta
2221 ttcattggaaccaaaaaaaaaaaaaaaaagaaaaaagtaaatcaagaaacatattgaactt
2281 tgaatagaagaaaatggaaataaaggatttatatccaacctcaactagtttgggaaattg
2341 ggattaaggcttggtttttgttgttgttgtactgaacttttgaaggcaacctatcttgga
2401 acatggtttaagggtagctacatgatggcgcattggcacttatgggatatacttactgaa
2461 aagaagaagggtttatctttttattggcatattttatggtgtatatatattttcaaatgag
2521 aaaatcatcattcatattatgtagtcctttctgaatgacaggctcggactcccactcctt
2581 gcccacctctgattataacagattaataatgcagttctaacaaatgagataataaatcgc
2641 tatatttgataaagcacttggtcaatgaacatattatgaaattgaaatggacatctctac
2701 ataattctgtcagtggaagatttcaaattgcattcatctttttatacccctgattaaatct
2761 tttctctacagtattttggcatctttattaaaaagtaaaaaacattcgttggtttaatag
2821 taatattccagcttaactaacgatggagaattaccttattgcttttatattgaccggatg
2881 agaagcaagtttgctttttacccttctatgattaggatcggagattaatggtaggaaggg
2941 aagggaaaggaaagaaatattaatcttatttccttgtctgttggtatctggctagtaga
3001 agtgtaaattttagatttagtgcatctatattaattaataattattcaatttgtcaagac
3061 tatggagataaggaaaacaaataaattgtctttttttgaaatcctttgggacactcactct
3121 ctgatttaatatagacgatgatgtaggaagttctttctttgtcttaatgatggtgctaac
3181 caggaaaatggttgacttctgttgacagtagtggttgtctatggaagttgaagacatgga
3241 ttgtataacatggaaagtaacatccatagagaaaagtggagatgttgatagtgaggtgga
3301 actataatctcactgctggggtggagatggtgcaatgttgtaaatagagattggaatata
3361 aaataatcgtacaaagatatatttgttttgaaactataaggtttgagattattacatgct
3421 gtattgatccaaaaatgattgttattgactcatgcaaaggtctttgagagatattgagga
3481 tcatgggaattttagggataattcggggaaggaaatgaaatgccacgacaagcgtttgat
3541 ctcaaagtttacttttgactgtaggctttgaagattaaattgataatggcctccacaata
3601 gtttttacaagttagttccagtagattttacaacctgcctttcgatgttatacttttagg
3661 atctgttacctgccttttgcccttcaggattactgtttgatcagattaaactcctttgcg
3721 atttgtttgacatgggacctgggtttttgcagattgatctcccatctttcatattatgtt
3781 ttactctcttcttctctcttgtattggcattacctattttgatattaggctaattgatga
3841 tcagtctacttgcactgctctaacttctaatgaactaaataaattgtgggatgatgtaac
3901 ctaatatggtgtaatgtaccaagccacatcttttaacgctaatctttaagcatatcatct
3961 tttcctttaggttttgggagaaaagttagggagctttgccacaatcctcgtttgggtagg
4021 atttgagttatatatgataaaatcagattgctctcctgcggaaagaaattactgggtctt
4081 ccagcaaaattgcctgcattaggacaagtcatattctctatgaattaattgtttattact
4141 ttaactaagtctttacaaatgtacaccaaagttatttgcttttcacataaactatcatca
4201 gaagaatctgaaaatataggtctgggatactgagttggctcttcaattcaactttttaga

4261 cttccatcctgatactttttacttcttcatggattggtttagtaaggatgcagattgagt
4321 tcccttgatagcatgcttatccctgatggaaatgatgtatttgtccttctactaaaccaa
4381 aatctttttactttttaaaaaaaaaaaaaaatttggtgttccgactagcaactttacagt
4441 ctagagtcaacttgaataccattgagccaaggctcattggtgaaatctcgtttggttatt
4501 gaaggctctgttagaatgtccatatagttatataaaacattgtaaatgtgtatatatgtg
4561 tttatatgtgtatatcacatatgtttttcagtttgccctttcctttaaaagatcatttag
191 A F G W A Y I N
4621 aaaccttactaaatattggaatcattatttctgcagGCTTTTGGGTGGGCATACATAAAC
199 K W H N T W E Q F Y V Y W I C P F I G A
4681 AAATGGCATAATACATGGGAACAGTTCTATGTGTATTGGATTTGCCCCTTCATAGGAGCA
219 I L A A W V F R L V F P L P A P K Q K K
4741 ATATTGGCTGCTTGGGTCTTCCGCTTAGTCTTCCCTCTGCCAGCACCAAAACAGAAGAAA
239 A *
4801 GCC**TAG**aagagcgcattatgttcatcacatggtagataagcatatgctgtaataaatact
4861 ttcacatttttcaactctttaattattgaagcgatctttagttaagttgctttattgctt
4921 gtattcatggaaattgacaagtctctcttctttcttcctctcccacatgttactttcttt
4981 catgccatttttatttctttatgtgactttatttctcaatgaaatatgcagaagattttg
5041 cagggcaagtaggtaggataggcgcatgcaagtaatgccattttagtgcatattcattcc
5101 tggggaatgaactgtcggattgaattgtcataaaaattacaagatgatgccttgtatatc
5161 aattggtggagagtgaactctgttaagcagttttggtacttgccaaaatcatgcatatca
5221 gcccttatatggttgtacaggtcgggctgagcttgagctcaccaaaactaggttcggcag
5281 agctcagctcagcctacacaaccatataagggtatgctctagctggatcagttcattgta
5341 aaatagccaagctcgaacaaggctgggg
