# Supplementary material for: Genome-Wide Identification of Jatropha curcas Aquaporin Genes and the Comparative Analysis Provides Insights into the Gene Family Expansion and Evolution in Hevea brasiliensis
Source: Front Plant Sci. 2016 Mar 31;7:395. doi: 10.3389/fpls.2016.00395 (PMC4814485; doi:10.3389/fpls.2016.00395)
Supplement: Supplementary file 6 [file Image2.PDF]

Supplementary File S2 The gene model for *JcNIP7;1*. The coding region is marked with uppercase letters (the intergenic spacer between two identified genes is also shown in uppercase letters but labeled with single underlines), above which is its deduced amino acids. The transcribed untranslated regions, including 5' UTR, intron and 3' UTR sequences, are marked with lowercase letters. The start and stop codons are marked with bold letters.

1 M A F P K T

1 attatcatctttttcttctcttattcaacctgttttcttttca**ATG**GCTTTTCCCAAAAC

7 S M P Q L M L F L F L I S I S F S L A Q

61 TTCCATGCCTCAACTAATGCTTTTCTTATTTCTCATTTCCATCTCTTTCTCCCTTGCCCA

27 T A S K P K A L V L P V S K D A T T L Q

121 AACGGCTTCAAAACCCAAAGCCCTAGTACTTCCAGTCTCCAAAGATGCAACAACTCTCCA

47 Y L T K F K M G T P P T K R S F V V D L

181 ATATCTAACGAAATTTAAAATGGGAACCCCTCCCACCAAAAGAAGTTTTGTGGTAGACCT

67 G G R H L W M D C D D G S Y L S S T L K

241 TGGAGGCCGACATTTGTGGATGGATTGTGATGATGGGTCATACTTATCATCCACACTCAA

87 H S F C G S A P C S V A K A Q C F G Q C

301 GCATAGCTTTTGTGGTTCAGCTCCATGTTCTGTAGCCAAAGCTCAATGTTTTGGTCAATG

107 Q P G R R R P G C D K E H C Y I L A D N

361 CCAACCTGGTAGGCGCAGACCAGGTTGCGATAAAGAACACTGTTATATCCTTGCTGACAA

127 T I P A L G S G F D I G L I S L D K I A

421 CACAATTCCTGCTTTAGGTAGTGGATTTGATATTGGATTGATTTCCTTAGACAAAATTGC

147 L Q S T D G S K F G P T V T V S D F I F

481 CCTCCAATCCACGGACGGTTCAAAATTCGGCCCAACTGTCACAGTTTCCGATTTTATCTT

167 G C L G A R E R L S N L A K G A D G M I

541 CGGTTGTTTAGGGGCTCGTGAGCGCCTAAGCAATCTTGCTAAAGGTGCAGACGGGATGAT

187 G L G R Q P I T L P T Q L S S G G S F R

601 TGGCCTCGGAAGGCAACCAATTACTCTACCGACACAACTTTCTTCTGGTGGTAGCTTCCG

207 K K F A I C L P S T P K L N G V M F F G

661 GAAAAAGTTCGCTATATGCTTGCCTTCAACTCCAAAATTAAACGGCGTTATGTTCTTTGG

227 D S P Y A F Y P S Y N T S K T I D V S T

721 CGATTCTCCTTATGCATTTTATCCAAGTTATAATACATCGAAGACCATTGATGTATCAAC

247 R F H Y T K L Y V R T E F S G S S I V T

781 CAGATTTCATTACACAAAACTCTACGTCCGTACTGAGTTCTCAGGATCATCCATCGTGAC

267 R G P P S P E Y F V N V T S I L V N R K

841 ACGAGGACCTCCATCGCCGGAATACTTTGTCAACGTCACTTCTATCTTGGTAAACAGAAA

287 P I F I N P T F L E F H R N G K G G A K

901 ACCCATTTTCATAAACCCAACATTTCTTGAATTCCATAGGAATGGGAAGGGAGGAGCGAA

307 I A T V E P Y T K L E T T I Y K S L V K

961 AATAGCAACAGTAGAACCTTACACCAAACTTGAAACCACTATCTATAAATCTCTTGTTAA

327 A F D K E I A V L S G S K V S P V A P F

1021 GGCGTTTGATAAGGAGATTGCAGTTTTGAGTGGGAGTAAGGTGTCCCCTGTGGCACCTTT

347 T D C Y K I D H I G M T P L G I G V P D
1081 TACAGATTGCTATAAGATAGATCATATAGGAATGACACCTCTTGGAATTGGGGTCCCAGA
367 L A F E F E N N K N E Q W E M Y G F N S
1141 TCTTGCATTCGAGTTCGAGAACAACAAAAATGAGCAGTGGGAAATGTATGGGTTTAATTC
387 M V E V S R D V A C L A F L D A G D D P
1201 AATGGTGGAAGTGAGTCGTGATGTGGCTTGTTTAGCATTCTTAGATGCAGGTGATGATCC
407 I V T T P I V I G A H Q L Q D N L L Q F
1261 TATTGTAACTACACCAATAGTGATAGGAGCACACCAGTTGCAGGATAATCTTCTTCAGTT
427 D L A S N R L A F T R T L L L A A A E C
1321 CGATCTTGCTTCTAACAGGCTTGCATTTACTAGAACACTTCTATTAGCAGCTGCTGAATG
447 S N F K F *
1381 TTCCAACTTCAAGTTT**TAA**gatgatgtaattatttatttatatgtggttaattaactgct
1441 tttgaagtttgttgatgcaagaattgctttttgcgattaattcaagttcagttcgtagcg
1501 agggaatcctcatctctcttgtgttcaactgaaattcgtgcattttgtaatctcactttg
1561 gcccataacttctggataattatattaatttatcaattgggcccattttgagttaaaagc
1621 tataaagcccacgaaataatttccgc<u>TCTCGGAATTTTTTCAAAATGTTATTGAAGGTAG</u>
<u>1681 TAGACCTGATTAACCAAATTTAATAGTAATATTGGTAAAATGCTGATAGCGTTTTAAAAC</u>
<u>1741 TTATACAGTTAACAAGCACAAACAACACTCTTTAATCCATCTACAGGTGTTAAAAACAAG</u>
<u>1801 TTCATCTTAGTTCAATTTCCTAACAAACATCAATCTTC</u>tatctaaacaatcaaacatatt
1861 tacttatgtctctttaaaatgacctccaatttatatttaagctgattgcatgcaagaaac
1921 atctctccacctcctttcaattttccatttgccctcatacttcttttcatgtaagtgttc
1981 gttaatttatcagaattgtaatttcaatcttgcctgacatttctataactatacattaac
1 M E S N A M S L H E D V F T K
2041 tttatctagagattgtc**ATG**GAATCAAATGCAATGTCATTACATGAAGATGTTTTCACTA
16 F F P Q G I D L N P A R M
2101 AATTCTTTCCTCAAGGAATTGATCTAAATCCGGCAAGAATGgtaagaaatgactaattta
29 V I A
2161 gtttcttaaaaagcctgcattatgatgtaattgcaactaagaaatgcgcagGTGATAGCA
32 E M M G T F V L M I C V C G I I G T T Q
2221 GAGATGATGGGGACATTTGTATTAATGATATGTGTGTGTGGGATCATTGGAACCACGCAG
52 L T R D Q L G L L E Y A T T A G L T V I
2281 CTAACGCGAGACCAATTGGGTCTGTTGGAATATGCAACAACAGCAGGATTAACAGTCATC
72 V L V F S I G P I S G A H V N P A I T I
2341 GTCTTGGTTTTCTCCATAGGCCCTATTTCTGGAGCTCATGTGAACCCTGCTATCACAATT
92 A F A T F G H F P W S R
2401 GCCTTTGCAACTTTTGGTCATTTTCCATGGTCCAGGgtaaatattatatatccctctttc
104 V P F Y
2461 aaaaccacttggatcacctaacctgatcttgtttaattaatatggcagGTTCCGTTTTAT
108 V S A Q I V G S A L A S Y A G G S I Y G
2521 GTATCAGCACAAATCGTTGGATCTGCATTGGCATCATATGCCGGAGGATCCATTTACGGC
128 I K P D L M T T R P F H G C S S A F W V
2581 ATCAAACCTGATCTAATGACCACCCGACCTTTCCATGGATGCAGTTCTGCTTTCTGGGTC
148 E F I A T F I I M F V A A S L A Y Q T S
2641 GAATTCATTGCAACCTTCATCATCATGTTTGTTGCTGCTTCACTCGCATATCAAACATCA

2701 gtaaggaaacattcactgtataattttaaacccttttttaaggacctaataaccagacttg

168 V R Q L S G F V L G V A I A

2761 aatttgaaaacttttacagGTAAGACAGTTGTCAGGCTTTGTTCTTGGAGTTGCCATTGC

182 L A V L I T G

2821 ACTTGCAGTACTTATTACAGGgtatatcatggcttatgagcatcccttttttttttcccct

2881 taaattcattatatactctagagttatccagtcatgggattggttataattcatatttca

189 P L S G G S L N P A R S L G P A I V

2941 tgttagGCCCCTTTCAGGAGGATCGTTGAATCCTGCAAGGTCATTAGGGCCTGCAATTGT

207 S R N F K D I W V Y I T A P I L G S I T

3001 TTCCAGGAACTTCAAGGACATATGGGTGTATATTACTGCGCCAATCTTAGGATCTATCAC

227 G A L M F H V L R I Q R R P C S S T S S

3061 AGGTGCTCTCATGTTTCATGTCCTGCGTATTCAACGCCGGCCATGCAGTTCTACTTCCTC

247 P D T G L L A H S M D F G G T V D S S *

3121 TCCTGATACGGGTCTACTTGCTCACTCTATGGACTTCGGTGGGACGGTGGATTCTAGC**TA**

3181 **A**atccacacaaggttgtgttgtggattaaatgcaattgccttcagaattaatgaagcgta

3241 acaaaaaccgcc
