# Supplementary material for: Genome-Wide Identification of Jatropha curcas Aquaporin Genes and the Comparative Analysis Provides Insights into the Gene Family Expansion and Evolution in Hevea brasiliensis
Source: Front Plant Sci. 2016 Mar 31;7:395. doi: 10.3389/fpls.2016.00395 (PMC4814485; doi:10.3389/fpls.2016.00395)
Supplement: Supplementary file 7 [file Image3.PDF]

Supplementary File S3 The gene model for *JcXIP1;1*. The coding region is marked with uppercase letters, above which is its deduced amino acids. The intron sequences are marked with lowercase letters. The start and stop codons are marked with bold letters.

1 M E L A S A Q E D N N Q Q F S I S H V N

1 **ATG**GAATTGGCCAGCGCCCAAGAAGATAATAATCAACAATTCTCCATATCACATGTAAAT

21 F E A M N S F K E T I R T T K T G F L F

61 TTCGAAGCAATGAATAGCTTCAAAGAGACTATAAGAACCACAAAGACAGGGTTTCTTTTT

41 R I G A H E L F S Q E

121 CGCATTGGTGCCCATGAATTGTTTTCACAAGAGgtaatgatatatgagcttcaaacttct

181 tccaattattgcttgtttatacatacataaatggctgcccgactcatatagttcagttct

241 tgtttttaagaaactggaattgaaatcatttaaataaaaaaaaaaaaccaaaattctggt

52 M W R A A L T

301 gtttatgtacttatattatattatgttaattatttccagATGTGGAGGGCAGCACTAACT

59 E L V A T A C L L F T L T I S I I S C L

361 GAGCTAGTAGCAACAGCTTGTCTTCTCTTCACATTAACAATTTCCATAATTTCATGCTTG

79 D S H E A D P K L L I P F T I F I I A F

421 GACTCACACGAGGCAGACCCCAAACTTCTAATCCCATTTACCATTTTCATAATTGCCTTC

99 F L L L T T I P L S G G H M S P I F T F

481 TTCCTCCTCTTAACAACAATCCCCTTATCAGGAGGTCACATGAGCCCGATCTTCACCTTC

119 I A A L K G L T T L V R A L F Y I L A Q

541 ATCGCAGCCCTAAAAGGACTCACAACTCTTGTCCGAGCCTTGTTCTATATATTAGCACAA

139 C I G S V M A Y M L I K S V M D H R I A

601 TGTATAGGCTCTGTAATGGCATATATGTTAATAAAGAGTGTAATGGACCACAGAATAGCA

159 E K Y Y L G G C I I D G N G K G I A Q T

661 GAAAAGTACTATTTAGGTGGCTGCATAATCGATGGAAATGGGAAAGGGATAGCACAAACA

179 T A L V L E F C C T F V V L F V G V T V

721 ACTGCTCTGGTGTTGGAATTTTGTTGCACTTTTGTGGTATTATTTGTGGGTGTAACGGTA

199 G F D T R R F K E L G L V M V C V I L A

781 GGATTTGATACAAGAAGGTTTAAGGAATTAGGGTTAGTTATGGTGTGTGTTATATTGGCA

219 A S M G L A V F V S I S V T G R A G Y G

841 GCTTCAATGGGACTTGCAGTTTTTGTGTCAATTAGTGTAACTGGAAGGGCTGGTTATGGT

239 G V G L N P A R C L G P A L L H G G S L

901 GGAGTTGGGTTGAACCCTGCTAGATGTTTAGGACCAGCTTTGTTGCATGGAGGTTCTTTG

259 W D G H W V F W V G P F L A C I F Y H A

961 TGGGATGGGCATTGGGTTTTCTGGGTCGGTCCATTTTTGGCTTGTATTTTTTACCATGCC

279 F S F T L P R Q E M E S V D E N *

1021 TTTTCCTTTACCTTACCAAGACAAGAAATGGAAAGTGTAGATGAGAAT**TAA**
