# Supplementary material for: Genome-Wide Identification of Jatropha curcas Aquaporin Genes and the Comparative Analysis Provides Insights into the Gene Family Expansion and Evolution in Hevea brasiliensis
Source: Front Plant Sci. 2016 Mar 31;7:395. doi: 10.3389/fpls.2016.00395 (PMC4814485; doi:10.3389/fpls.2016.00395)
Supplement: Supplementary file 8 [file Image4.PDF]

Supplementary File S4 The gene model for *JcSIP1;2*. The coding region is marked with uppercase letters, above which is its deduced amino acids. The transcribed untranslated regions, including 5’ UTR, intron and 3’ UTR sequences, are marked with lowercase letters. The start and stop codons are marked with bold letters.

1 ggttaggtacgtctgcgtgtatacaaagcggtggtggaaatttcaaagtcttgctatgca
61 aaagaggacaacgaaagggatctttagaatctcttttctacgctttacatcgctaaacga
121 cataatcctcacttggccttcacttttcttgtttttatcttcagtttcaaacctgcaagc
1 M S L I K A A M A D S I L T T M
181 ggacttgcagat**ATG**AGTTTAATCAAAGCAGCCATGGCAGATTCAATTTTAACCACCATG
17 W V F S L P F L G I F T S I I A S N I G
241 TGGGTCTTTAGCCTGCCATTTCTCGGCATTTTCACTTCCATTATAGCATCAAATATAGGC
37 V E P K S I P A L F I A I N I A T P F V
301 GTTGAACCCAAATCAATACCAGCCCTTTTCATAGCTATAAATATTGCCACTCCTTTTGTT
57 L I F S L I G A A L G G A S F N P T T T
361 CTAATTTTCAGCCTGATCGGCGCCGCGTTAGGAGGTGCCAGTTTCAACCCAACAACCACC
77 V S L Y A A G L K P D V S L I S M A I R
421 GTATCATTATATGCCGCGGGGCTTAAGCCAGATGTGTCTTTAATATCCATGGCGATCCGG
97 F P A Q A A G G V F G A K A I L Q F M P
481 TTTCCTGCTCAGGCGGCTGGTGGAGTTTTTGGAGCCAAAGCAATTTTGCAATTTATGCCA
117 I K Y K N F L K G P S L K V D L H T G A
541 ATAAAATATAAAAATTTCCTGAAGGGTCCTTCTTTGAAAGTGGATTTGCATACAGGTGCA
137 T A E G V L S F V F C L F L L I V L V K
601 ACTGCAGAAGGGGTTTTGAGTTTTGTGTTTTGCCTTTTTTTGCTTATTGTTTTGGTTAAA
157 G P K N F L V K V W L L A V A T V G L V
661 GGGCCCAAGAATTTTTTGGTCAAGGTCTGGTTGCTGGCGGTGGCGACGGTGGGTTTGGTG
177 V T G G K Y T G P S L N P A N A Y G W A
721 GTTACCGGCGGGAAATATACAGGGCCTTCCTTAAACCCTGCTAATGCTTATGGATGGGCT
197 Y M N N W H N S W E L F Y V Y W I C P L
781 TATATGAACAATTGGCATAATAGTTGGGAGTTGTTTTATGTGTATTGGATTTGCCCTTTG
217 I G A T L A A W V F R L L F S F S P P V
841 ATTGGAGCAACTTTGGCTGCTTGGGTTTTCCGTTTACTATTTAGCTTTAGCCCTCCTGTT
237 V K P K Q A *
901 GTCAAGCCTAAGCAAGCT**TGA**aaagaagggcatattaatgatagtgatagctctctctct
961 cctctctctctctctctttttccacacagcac
