# Supplementary material for: Genome-Wide Identification of Jatropha curcas Aquaporin Genes and the Comparative Analysis Provides Insights into the Gene Family Expansion and Evolution in Hevea brasiliensis
Source: Front Plant Sci. 2016 Mar 31;7:395. doi: 10.3389/fpls.2016.00395 (PMC4814485; doi:10.3389/fpls.2016.00395)
Supplement: Supplementary file 9 [file Image5.PDF]

**Supplementary File S5 Alignment of deduced amino acid sequences of physic nut AQPs with structure determined Spinach PIP2;1.** Multiple alignments were performed using ClustalX. Transmembrane helices (TM1–TM6) and the two short helices forming the two NPAs (HB and HE) (shaded), $P_1$–$P_5$ residues (shown in blue), NPA motifs (shown in red), and ar/R selectivity filter residues (shown in green) are indicated. The highly conserved cysteine residues in XIPs are shown in **bold**. The positions corresponding to S115 and S274 in SoPIP2;1（Genbank accession number 1Z98）are highlighted in bright green. The residues corresponding to S262 in GmNOD26 (Genbank accession number P08995) are shown in sky blue. The residues at the position corresponding to L197 from SoPIP2;1, determined to be the key residue involved in gating (occurring just before TM5) are underlined.

```
                                                                       TM1
SoPIP2;1     -------MSKEVSE-EAQAH--------------QHGKDYVDPPPAPFFDLGELKLWS--
JcPIP1;1     -------MEGKEEDVRLGANKFRETQPIGTAAQSQDDKDYTEPPPAPLFEPTELTSWS--
JcPIP1;2     -------MEGKEEDVRLGANKFTERQPIGTAAQT--DKDYKEPPPAPLFEPGELSSWS--
JcPIP1;3     -------MEGREEDVKMGAN----------------------------------------
JcPIP1;4     -------MEGREEDVRVGATKFPEKQAIGTSAQT--DKDYKEPPSTPLFEPGELQSWS--
JcPIP2;1     -------MGKDVE----GGD--------------FQAKDYHDPPPAPLIDAEEFTQWS--
JcPIP2;2     -------MAKDVEVAEPTGE--------------FSGKDYHDPPPAPLIDMDELGQWS--
JcPIP2;3     -------MVKDVAE---QGS--------------FSAKDYHDPPPAPLIDAEELTKWS--
JcPIP2;4     -------MAKEVSE-ETQT---------------THAKDYVDPPPAPLIDMAEIKLWS--
JcPIP2;5     -------MANKELG-EEVAH--------------QHGKDYVDPPPAPLLDIEELTKWS--
JcTIP1;1     -------------------------------------MPIR--NIAIGHPQE-ATHPD--
JcTIP1;2     -------------------------------------MPIT--RIAIGNPGE-ASQPD--
JcTIP1;3     -------------------------------------MPIT--SIAIGSPAE-VGQAD--
JcTIP1;4     -------------------------------------MPPVNFRIAVGLPHEDITHPG--
JcTIP2;1     -----------------------------------------MAKIAFGRFDD-SFSLG--
JcTIP2;2     -----------------------------------------MVKIAFGNFGD-SFSVG--
JcTIP3;1     -------------------------------------MPPR--RYIFGRTEE-ATHPD--
JcTIP4;1     -----------------------------------------MAKIALGSRRE-ATQPD--
JcTIP5;1     -----------------------------------------MAPTSLIVRFQQSVSPD--
JcNIP1;1     ---------------------------MGEIAGS-NGKQ---VVLDVKDDNNPPPCPS--
JcNIP2;1     ---------------------------MATTSAS-DHTASPSTEYLVSVENPKSENPF--
JcNIP3;1     ---------------------------MASHSIT-STDISPKLQLPVKRP--VLQVPE--
JcNIP3;2     ---------------------------MAAVSPSPLDEFSPKQPRVVITTPDLITIEE--
JcNIP4;1     ---------------------------MPDIIEE-ETQIS-NTEKGLVVKSSKPNNPN--
JcNIP5;1     ------------------------MPESEAGTPTVSAPATPGTPGGPLFSSLRVDSLS--
JcNIP6;1     ------------------------MDNNEEIPSAPSTPATPGTPGAPLFGGFKGTERAGG
JcNIP7;1     ---------------------------MESNAMSLHEDVFTKFFPQGIDLNP--------
JcXIP1;1     ----MELASAQEDNNQQFSISHVNFEAMNSFKET---IRTTKTGFLFRIGAHELFSQE--
JcXIP2;1     MAENLRFVEDEENEHGGTKIQPLASTPMPNLDKTEGGKKQSPTNMNKVLGLEELSSSN--
JcSIP1;1     ------------------------------------------------------------
JcSIP1;2     ------------------------------------------------------------
JcSIP1;3     ------------------------------------------------------------
JcSIP2;1     ------------------------------------------------MGDGSVTS----
```

**TM1**

```
SoPIP2;1        -----------------------------------------FWRAAIAEFIATLLFLYIT
JcPIP1;1        -----------------------------------------FYRAGIAEFIATFLFLYIS
JcPIP1;2        -----------------------------------------FYRAGIAEFVATFLFLYIT
JcPIP1;3        -------------------------------------------RAGIAEFVATFLFLYIT
JcPIP1;4        -----------------------------------------FWRAGIAEFMASFLFLYVA
JcPIP2;1        -----------------------------------------FYRAIIAEFIATLLFLYIT
JcPIP2;2        -----------------------------------------FYRALIAEFIATLLFLYIT
JcPIP2;3        -----------------------------------------FYRALIAEFIATLLFLYIT
JcPIP2;4        -----------------------------------------FYRALIAEFIATLLFLYIT
JcPIP2;5        -----------------------------------------FYRAVIAELVATLIFLFVT
JcTIP1;1        -----------------------------------------ALKAALAEFISTLIFVFAG
JcTIP1;2        -----------------------------------------ALRAALAEFFSMVIFVFAG
JcTIP1;3        -----------------------------------------ALKAALAEFISTLIFVFAG
JcTIP1;4        -----------------------------------------AIKAALAEFISTAIFVFAG
JcTIP2;1        -----------------------------------------SFKAYLAEFISTLLFVFAG
JcTIP2;2        -----------------------------------------SLKAYLSEFIATLLFVFAG
JcTIP3;1        -----------------------------------------SIRATLAEFVSTLIFVFAG
JcTIP4;1        -----------------------------------------CIKALIVEFITTFLFVFAG
JcTIP5;1        -----------------------------------------ALRSYLAEFISTFFYVFAV
JcNIP1;1        ----------------------KHKEDSALSISVP------FMQKMIAEVAGTYFLIFAG
JcNIP2;1        ----------------------LIPLLSFQNHYPPR-----FFRKVVAEMIATFLLVFVT
JcNIP3;1        ----------------------DNPNSTPSSFLLF--------QKILAEFLGTYVLIFIG
JcNIP3;2        ----------------------GKPTPSQASTTDIPVVSPNNAHKIIAEMVGTYVIVLIG
JcNIP4;1        ----------------------DNTASRSSTCFVT------IIQKLVAEVIGTYFVIFAG
JcNIP5;1        YDRKSMPR-CKCLPVN--APSWGQTHTCFTDFPAPD---VSLTRKLGAEFVGTFILIFAA
JcNIP6;1        LTRKSILNGCRCFSIEEWALEEGRLPPVSCSIPPPP---VSLARKVGAEFIGTLILIFAG
JcNIP7;1        ------------------------------------------ARMVIAEMMGTFVLMICV
JcXIP1;1        -----------------------------------------MWRAALTELVATACLLFTL
JcXIP2;1        -----------------------------------------VWRASLAELLGTAALVFAM
JcSIP1;1        ---------------------------------------MGAVKAAIGDGVLTFMWVFCS
JcSIP1;2        ---------------------------------------MSLIKAAMADSILTTMWVFSL
JcSIP1;3        ---------------------------------------MGLIRVAMADAILTSLWVFSV
JcSIP2;1        -------------------------------------------RLIISDFVISFMWVWSG
                                                           :    :       :
```

```
                  TM1                         TM2                       HB
SoPIP2;1        VATVIGHSKETVV------CGSVGLLGIAWAFGGMIFVLVYCTAGISGGHINPAVTFGLF
JcPIP1;1        VLTVMGVVKAPTK------CSTVGIQGIAWAFGGMIFALVYCTAGISGGHINPAVTFGLF
JcPIP1;2        VLTVMGVSKSGNK------CATVGTQGIAWAFGGMIFALVYCTAGISGGHINPAVTFGLF
JcPIP1;3        FLTVIGVAKSPTK------CSTVGIQGIAWAFGGMIFALVYCTAGISGGHINPA------
JcPIP1;4        ISTVMGYSRSSNK------CATVGVQGIAWAFGGMIFALVYSTAGISGGHINPAVTFGLL
JcPIP2;1        VLTVIGYKSQTDPSKNSDACGGVGILGIAWAFGGMIFILVYCTAGISGGHINPAVTFGLF
JcPIP2;2        VLTVIGYKSQTDPNKNADACGGVGILGIAWAFGGMIFILVYCTAGISGGHINPAVTFGLF
JcPIP2;3        VLTVIGYKSQTDPAKNADSCGGVGILGIAWAFGGMIFILVYCTAGISGGHINPAVTFGLF
JcPIP2;4        VATVIGYKKQTDP------CGGVGLLGIAWAFGGMIFILVYCTAGISGGHINPAVTFGLF
JcPIP2;5        VATVIANKASTMSSG----CGGVGLLGVAWSFGSTIFILVYCTAGISGGHINPAVTFGLL
JcTIP1;1        EGSGMAFNKLTNNGAT----TPAGLVAASLAHGFGLFVAVSVGANISGGHVNPAVTFGAF
JcTIP1;2        EGSGMAFNKLTSDGSS----TPAGLVAASLAHAFALFVAVSVGANISGGHVNPAVTFGAF
JcTIP1;3        EGSGMAFNKLTSDGST----TPAGLVAASLAHGFALFVAVSVGANISGGHVNPAVTFGAF
JcTIP1;4        EGSGMAFNKLTDDGSS----TPAGIIMASLAHAFGLFIGVATAANISGGHVNPAVTFGAF
JcTIP2;1        VGSAIAYNKLTGNAAL----DPAGLVAIAICHGFALFVAVAVGANISGGHVNPAVTFGLA
JcTIP2;2        VGSAIAYSKLTADAAL----DPPGLVAVAVAHAFALFVGVSIAANISGGHLNPAVTFGLA
JcTIP3;1        EGSVLALEKLYRETGP----PASGLVMIALAHALALFAAVAASINISGGHVNPAVTLGAL
JcTIP4;1        VGSAMAADKLVGG-------PLVGLFFVAVAHALVVAVMISAG-HISGGHLNPAVTLGLL
JcTIP5;1        VGSSMAARKLMPAAD------PSSLIIVATANSFALSSAIYIAANISGGHVNPAVTFSMA
JcNIP1;1        CTAVAVN-----LNFD----KVVTHPGISIVWGLAVMVLVYSVGHISGAHFNPAVTLAFA
JcNIP2;1        CGTAAIS-----RSDE----KRVSELGASVAGGLIVTVMIYAVGHVSGAHMNPAVTTAFA
JcNIP3;1        CGSALTN-----DIQ------KLTILGIAIVWGVVLMALIYAVGHISGAHFNPAVSIALA
JcNIP3;2        CGSLIVD-----RING-----PLSVVGIAVAWGLVVMVMIYTFGHISGGHFNPAITIAFA
JcNIP4;1        CGAVAVN-----KIYG-----SVTFPGICVSWGLIVMVMIYTVGHVSGAHFNPAVTIASA
JcNIP5;1        TAGPIVN-----QKYS----GVETLIGNAACSGLAVMIVILSTGHISGAHLNPALTIAFA
JcNIP6;1        TATAIVN-----QKTQ----GAETLVGLAASSGLAVMIVILSTGHISGAHLNPSVTIAFA
JcNIP7;1        CGIIGTT-----QLTR----DQLGLLEYATTAGLTVIVLVFSIGPISGAHVNPAITIAFA
JcXIP1;1        TISIISCLDSHEA---------DPKLLIPFTIFIIAFFLLLTTIPLSGGHMSPIFTFIAA
JcXIP2;1        DTIVISTYETETK---------TPNLIMSALIAITVTILLIATSPISHGHINPVITFAAL
JcSIP1;1        SMFGLFTSLIAAALRVQN-YLWATLFITTVLVFVFVYLFGLIAGFLGGASFNPTGNAAFY
JcSIP1;2        PFLGIFTSIIASNIGVEP-KSIPALFIAINIATPFVLIFSLIGAALGGASFNPTTTVSLY
JcSIP1;3        PILRVLTTIIASTVGVEP-KSLSGLFISINLSTSFMLIFILVGAALGGASYNPTTTVSLY
JcSIP2;1        ALIKLFVNHVLGLAHQ-----PSGEAINYALSVIVMFFFAFLGKITKGGGYNPLTIFSSA
                                                                .  .*
```

**TM3**

```
SoPIP2;1    LARKVS-----LLRALVYMIAQCLGAICGVGLVKAFM-KGPYNQFGGGANSVA-------
JcPIP1;1    LARKLS-----LTRAIYYMVMQCLGAICGAGVVKGFEGRKQYTLLGGGANSVA-------
JcPIP1;2    LARKLS-----LTRALFYIIMQCLGAICGAGVVKGFEGNRVYESLGGGANVVA-------
JcPIP1;3    ----------------------------------GFE-KNQYERLGGGANTIS-------
JcPIP1;4    LARKVS-----LTRAIFYMVMQCLGAICGAGVVKGFQ-PTPYQMGGGGANMVQ-------
JcPIP2;1    LARKVS-----LVRAILYMAAQCLGAICGCGLVKAFQ-KAYYTRYGGGANELA-------
JcPIP2;2    LGRKVS-----LIRALGYMIAQCLGAICGCGLVKAFQ-KAYYNRYGGGANELA-------
JcPIP2;3    LARKVS-----LVRAVMYMVAQSLGAICGVGLVKAFQ-SAYYKRYGGGANTLA-------
JcPIP2;4    LARKVS-----LIRALAYMVAQCLGAICGVGLVKAFM-KNPYNHLGGGANSVN-------
JcPIP2;5    LARKVS-----LNRAVAYIVAQCVGAIIGVALVKGLV-KDLYKSLGGGANSVT-------
JcTIP1;1    VGGNIT-----LLRGILYWIAQLLGSTVACLLLKFSTGGLTTSAFALSSGVG--------
JcTIP1;2    IGGNIT-----LLRGILYWIAQLLGSVVACLLLKYATGGLETSAFALSSGVS--------
JcTIP1;3    VGGHIT-----LMRSILYWVGQLLGSVVACLLLKFATGGLETSAFALSSGVS--------
JcTIP1;4    VGGNIT-----LLRGILYWIAQLLGSTVACLLLKFSTHGMTTSAFALSSGVN--------
JcTIP2;1    LGGQIT-----ILTGIFYWIAQLLGSIVACLLLKVVTGGLETPTHSLAAGVG--------
JcTIP2;2    IGGHIT-----LLTGLFYWIAQSLGSIVACLLLKFVTNGKSIPTHGVASGVN--------
JcTIP3;1    VGGRIS-----VVRAFYYWIAQLLGSIVASLLLRLVTNGMRPEGFHVTAGVG--------
JcTIP4;1    FGGHIT-----LVRSILYWIDQLLASSAACLLLNYLTGGLATPVHTLASGVG--------
JcTIP5;1    VGGHIS-----VPTALFYWISQMLASVMACVFLKVAIVGQNLPTYTIAEEMT--------
JcNIP1;1    TCKRFP-----WKQVPAYIVCQVIGSTLAAGTIRLIFTGKQDHFVG-TMPAG--------
JcNIP2;1    AVREFP-----WKQVPFYAVAQMTGAIGASFTLKVLLH--PIKQLGTTSPSG--------
JcNIP3;1    AVRKFS-----WKHVPVFVLAQVLGSTLAILTLKVLFH-DQDDIQATMTQYKNS------
JcNIP3;2    ISCKYP-----WRQVPGYVASQLAGSTLAILTLNVMFHREKIDIKITTTQYEGR------
JcNIP4;1    IFRRFP-----FREVPLYIVAQVIGSILASGTLALLFDITPMAYFG-TLPVG--------
JcNIP5;1    ALRHFP-----WVQVPAYIAAQVSASICASFALKGVFH--PFMSGGVTIPSV--------
JcNIP6;1    ALNHFP-----WKHVPVYIGAQVLASLGAAFALKGILH--PIMGGGVTVPSG--------
JcNIP7;1    TFGHFP-----WSRVPFYVSAQIVGSALASYAGGSIYGIKPDLMTTRPFHGCS-------
JcXIP1;1    LKGLTT-----LVRALFYILAQCIGSVMAYMLIKSVMDHRIAEKYYLGGCIIDGNGK---
JcXIP2;1    FTGRVS-----LSRAAVYILAQCLGAILGALALKAVLNSTVEETFSLGGCTLSIVAPGPH
JcSIP1;1    AAGVGGD---NLFSLALRFPAQAAGAVGGVLAILEVIPPQYKHMLGGPSLKVD-------
JcSIP1;2    AAGLKP--DVSLISMAIRFPAQAAGGVFGAKAILQFMPIKYKNFLKGPSLKVD-------
JcSIP1;3    AAGLKPSGSLSLKTMAVRFPAQAAGGVVGVKAILQAMPRTYRNLLKGPSLKVD-------
JcSIP2;1    ISGDFS---QFLLTVGARIPAQVIGSIYGVRLIIETFPEVGRGPRLNVD-----------
```

```
                  TM4                       TM5
SoPIP2;1    ----LGYNKGTALGAEIIGTFVLVYTVFSATDPKRSARDS---HVPILAPLPIGFAVFMV
JcPIP1;1    ----PGYTKGDGLGAEIVGTFVLVYTVFSATDAKRNARDS---HVPILAPLPIGFAVFLV
JcPIP1;2    ----SGYTKGDGLGAEIVGTFVLVYTVFSATDAKRSARDS---HVPILAPLPIGFAVFLV
JcPIP1;3    ----AGYSKGDGLGAEIVGTFLLVYTVFSATDAKRNARDS---HVPILAPLPIGFAVFLV
JcPIP1;4    ----PGYSKGDGLGAEIVGTFVLVYTVFSATDAKRSARDS---HVPILAPLPIGFAVFVV
JcPIP2;1    ----DGYSKGTGLGAEIIGTFVLVYTVFSATDPKRNARDS---HVPVLAPLPIGFAVFMV
JcPIP2;2    ----DGYNKGTGLGAEIIGTFVLVYTVFSATDPKRNARDS---HVPVLAPLPIGFAVFMV
JcPIP2;3    ----DGYSTGVGLGAEIIGTFVLVYTVFSATDPKRSARDS---HVPVLAPLPIGFAVFMV
JcPIP2;4    ----TGYSKGTALGAEIIGTFVLVYTVFSATDPKRSARDS---HVPILAPLPIGFAVFMV
JcPIP2;5    ----AGFSIGTGLGVEILGTFFLEYTVLSATDPKRKARDS---HVPVLAPLPIGFTVFVV
JcTIP1;1    --------VWNAFVFEIVMTFGLVYTVYATAIDPKK------GSLGTIAPIAIGFIVGAN
JcTIP1;2    --------AWNAVVFEIVMTFGLVYTVYATAVDPKK------GNLGIIAPIAIGFIVGAN
JcTIP1;3    --------SWNALVFEIVMTFGLVYTVYATAVDPRK------GNLGTIAPIAIGFIVGAN
JcTIP1;4    --------VWNALVFEIVMTFGLVYTVYATAIDPRK------GQLGTIAPIAIGFIVGAN
JcTIP2;1    --------AIEGVVMEIIVTFALVYTVYATAADPKK------GSLGTIAPIAIGFIVGAN
JcTIP2;2    --------AFEGVVFEIVITFGLVYTVYATAADPKK------GSLGIIAPIAIGFIVGAN
JcTIP3;1    --------EVHGLIMEIVMTFGLVYTVYATAIDPKR------GSLGIIAPLAIGLIVGAN
JcTIP4;1    --------YLQGVVWEIILTFSLLFTVYATIVDPKK------GAIDGLGPTLTGFVVGAN
JcTIP5;1    --------GFGASILEGVLTFGLVYTIYAAG-DPRR------SLPGAIGPLAIGLVAGAN
JcNIP1;1    -------SNMQSFVVEFIITFYLMFVISGVATDNR--------AIGELAGLAVGATVLLN
JcNIP2;1    -------SDFQALVMEIVVTFSMMFVTSAVATDTK--------AIGELAGIAVGSAVCIT
JcNIP3;1    ------TSHLEAIIWEFIITFILMFNICAVATDHR--------ASKDFSGVAIGGTLLVN
JcNIP3;2    ------ATDLESFIWEFITSFILMLTICGVAIDTK--------AINELSGVAVGSAMLFD
JcNIP4;1    -------SNVQSLVIEIIITFLLMFVVSGVNTDDR--------AVGDLGGIAVGMTILLN
JcNIP5;1    -------STGQAFALEFLITFNLLFVVTAVATDTR--------AVGELAGIAVGATVALN
JcNIP6;1    -------GYGQAFALEFIISFNLMFVVTAVATDTR--------AVGELAGIAVGATVMLN
JcNIP7;1    ----------SAFWVEFIATFIIMFVAASLAYQT---------SVRQLSGFVLGVAIALA
JcXIP1;1    -----GIAQTTALVLEFCCTFVVLFVGVTVGFDTRRFKELGLVMVCVILAASMGLAVFVS
JcXIP2;1    GPILIGLETDRALWLEIICTFFFLFSSIWLAFDKRQSTPLGRVIVCCIIGLVVGLLVFIS
JcSIP1;1    --------LHTGAIAEGVLTFIITFVVLLIVLRGPR---------NSFVQNWLIAVATVT
JcSIP1;2    --------LHTGATAEGVLSFVFCLFLLIVLVKGPK---------NFLVKVWLLAVATVG
JcSIP1;3    --------LHTGGLAEGILSFGLCFSLLLVMVRGPK---------NLWVKIWLVKAATAG
JcSIP2;1    --------IHRGALTEGVLTFIIVIISLGLARKIPG---------SFFRKTWISSISKLA
                       .   *   :* .
```

```
                          HE                         TM6
SoPIP2;1        HLAT--IPITGTGINPARSFGAAVIFNSNKVWDDQWIFWVGPFIGAAVAAAYHQYVLRAA
JcPIP1;1        HLAT--IPITGTGINPARSLGAAIIFNKDQGWDDHWIFWVGPFIGAALAALYHQVVIRAI
JcPIP1;2        HLAT--IPITGTGINPARSLGAAIIFNKDHAWDDHWVFWVGPFIGAALAALYHQIVIRAI
JcPIP1;3        HLAT--IPITGTGINPARSLGAALIYNKEQAWDDHWIFWIGPFIGAALAALYHQIVIRAI
JcPIP1;4        NMAT--IPITGAGINPARSLGAAVIYNNDNGWDDHWIFWVGPFIGAALAALYHQIVIRAI
JcPIP2;1        HLAT--IPITGTGINPARSFGAAVIYNKDKAWDDQWIFWVGPFIGAAIAAIYHQYILRAG
JcPIP2;2        HLAT--IPITGTGINPARSFGAAVIYNKDKAWDDQWIFWVGPFVGAAIAAFYHQYILRAA
JcPIP2;3        HLAT--IPITGTGINPARSLGAAVIYNHDKAWDDQWIFWVGPFLGAAIAAFYHQFILRAG
JcPIP2;4        HLAT--IPITGTGINPARSFGAAVIYNNDKVWDDHWIFWVGPFVGAIAAAAYHQYILRAA
JcPIP2;5        HMAT--LPITGTGINPARSFGAAVIYNKKMVWDDHWIFWVGPLVGAAAAAAYHQIVLRAG
JcTIP1;1        ILAGG--AFDGASMNPAVSFGPALVSW---SWDNHWVYWAGPLVGGGLAGLVYEFFFIGH
JcTIP1;2        ILAGG--AFDGASMNPAVSFGPAVVSW---TWTNHWVYWVGPLIGAAIAALVYDNIFIGE
JcTIP1;3        ILAGG--AFDGASMNPAVSFGPAVVSW---TWSNHWVYWLGPFIGGGIAAVVYETFFISP
JcTIP1;4        VLAGG--AFEGASMNPAVSFGPALVSW---DWTHHWVYWAGPLIGGGLAGLIYETIFISR
JcTIP2;1        ILAAG--PFSGGSMNPARSFGPAVASG---DFHDNWIYWVGPLIGGGLAGLIYGNLYINN
JcTIP2;2        ILAAG--PFSGGSMNPARSLGPAVVSG---DFSQIWIYWLGPLIGGGLAGLVYGDIFIGS
JcTIP3;1        ILVGG--PFDGAAMNPARAFGPALVGW---RWSNHWIYWVGPFIGGALAGLIYEYMVIPT
JcTIP4;1        ILAGG--AFSGASMNPARSFGPALVSW---DWTDHWVYWVGPLIGGGLAGFIYENFFIIR
JcTIP5;1        VLAAG--PFSGGSMNPASAFGSAVVAG---RFKNQAVYWVGPLIGATVAGLLYDNVVFPN
JcNIP1;1        VIFAG--PISGASMNPARSLGPAIVSC---KFKGLWIYLVSPTLGAQAGAWVYNMIRYTD
JcNIP2;1        SILAG--PISGGSMNPARTIGPAIASA---YYKGIWVYIVGPVAGTLLGAWSYNLIRVTD
JcNIP3;1        VMVAG--PITGASMNPARSLGPAIVSG---VYKNLWVFLLSPVLGALAATMVYNMLRVPK
JcNIP3;2        MLIAG--NITGASMNPARSIGPALVSK---DFCGLWVYIFAPILGMIAASTMYTFLWPPT
JcNIP4;1        VFVAG--PVSGASMNPARSLGPAIVKH---TYKGLWVYIAGPIVGAIAGAFAYNLLRSTD
JcNIP5;1        ILVAG--PSSGASMNPVRTLGPAVAAG---NYKAIWIYLVAPILGGLAGGTTYTAVKLRE
JcNIP6;1        ILIAG--PATGASMNPVRTLGPAIAAN---NYKAIWIYLTAPILGALCGAGTYSAVKLPE
JcNIP7;1        VLITG--PLSGGSLNPARSLGPAIVSR---NFKDIWVYITAPILGSITGALMFHVLRIQR
JcXIP1;1        ISVTGRAGYGGVGLNPARCLGPALLHG-GSLWDGHWVFWVGPFLACIFYHAFSFTLPRQE
JcXIP2;1        TTVTAQKGYAGVGMNPARCLGPALIRG-GHLWSGHWVFWVGPIISCIAFGVYVKIIPVAN
JcSIP1;1        LVVTG-SKYTGPSMNPANAFGWAYINKWHNTWEQFYVYWICPFIGAILAAWVFRLVFP--
JcSIP1;2        LVVTG-GKYTGPSLNPANAYGWAYMNNWHNSWELFYVYWICPLIGATLAAWVFRLLFSFS
JcSIP1;3        LVVIG-GKYTGPCMNPANAYGWAYANNWHNSWDLFYVYWICPLIGATSAAWVFRFLFN-P
JcSIP2;1        LHILG-SDLTGGCMNPASVMGWAYARGDHITKEHILVYWLAPIEATLLAVWTFKLLVRPK
                          *  :**.   * *             ::   *  .           .
```

```
SoPIP2;1        AIKALGSFRSNPTN------------------
JcPIP1;1        PFKK----------------------------
JcPIP1;2        PFKSRA--------------------------
JcPIP1;3        PFKSK---------------------------
JcPIP1;4        PFKSRA--------------------------
JcPIP2;1        AVKALGSFRSTSNV------------------
JcPIP2;2        AIKALGSFRSNA--------------------
JcPIP2;3        AVKALGSFRSNPTV------------------
JcPIP2;4        AIKALGSFRSNPTN------------------
JcPIP2;5        AVKALGSFRGNPVM------------------
JcTIP1;1        N-----THEQLPTADY----------------
JcTIP1;2        G-----AHEPLSTSDF----------------
JcTIP1;3        N-----THEQLPSADF----------------
JcTIP1;4        ------THEPVPSPEF----------------
JcTIP2;1        ------DHAPL-SNDF----------------
JcTIP2;2        Y-----SPAPS-SEEYA---------------
JcTIP3;1        EPPLHHTHQPLAPEDY----------------
JcTIP4;1        S----HIPIPIVEENY----------------
JcTIP5;1        QVP---DSIRGISDGVRV--------------
JcNIP1;1        KPLREITKSASF-IKSTGRA------------
JcNIP2;1        KPVQAISPSFSFRIRRTSIDEQTNNKDPLSAV
JcNIP3;1        PDNPEEKTKNIFNHLYTHADP-----------
JcNIP3;2        QNVDKDNSKSV---------------------
JcNIP4;1        KPLDDE--------------------------
JcNIP5;1        DEADPPRQ----VRSFRR--------------
JcNIP6;1        EDGNARQKPLAATASFRR--------------
JcNIP7;1        RPCSSTSSPDTGLLAHSMDFGGTVDSS-----
JcXIP1;1        MESVDEN-------------------------
JcXIP2;1        A-------------------------------
JcSIP1;1        LPAPKQKKA-----------------------
JcSIP1;2        PPVVKPKQA-----------------------
JcSIP1;3        TINPKPKQA-----------------------
JcSIP2;1        KEEKKEKSEGKSD-------------------
```
