# Supplementary material for: Genome-Wide Identification of Jatropha curcas Aquaporin Genes and the Comparative Analysis Provides Insights into the Gene Family Expansion and Evolution in Hevea brasiliensis
Source: Front Plant Sci. 2016 Mar 31;7:395. doi: 10.3389/fpls.2016.00395 (PMC4814485; doi:10.3389/fpls.2016.00395)
Supplement: Supplementary file 10 [file Image6.PDF]

Supplementary File S6 SDP analysis of physic nut AQPs from alignments with putative amino acid sequences of AQPs transporting non-aqua substrates. Multiple alignments were performed using ClustalW. Shown is the sequences containing the nine SD positions which are highlighted in yellow and the representative sequences are marked in blue. The Genbank accession numbers: AtPIP1;2 (Q06611), AtPIP2;1 (P43286), AtPIP2;4 (Q9FF53), AtTIP1;1 (P25818), AtTIP1;2 (Q41963), AtTIP1;3 (NP_192056), AtTIP2;1 (Q41951), AtTIP2;3 (Q9FGL2), AtTIP4;1 (O82316), AtTIP5;1 (NP_190328), AtNIP1;1 (CAA16760), AtNIP1;2 (Q8LFP7), AtNIP5;1 (NP_192776), AtNIP6;1 (NP_178191), CmNIP2;1 (BAK09176), CpNIP1 (CAD67694), GmNOD26 (P08995), GmNIP2;1 (XP_003534451), GmNIP2;2 (NP_001240190), HvPIP1;3 (BAA23745), HvPIP1;4 (BAF33068), HvPIP2;1 (BAA23744), HvNIP2;1 (BAH24163), NtAQP1 (O24662), NtTIPa (Q9XG70), NtXIP1;1α (ADO66667), NtXIP1;1β (ADO66666), OsNIP2;1 (Q6Z2T3), OsNIP2;2 (Q67WJ8), OsNIP3;2 (Q7EYH7), SlXIP1;1α (ADO66672), SlXIP1;1β (ADO66671), StXIP1;1α (ADO66669), StXIP1;1β (ADO66670), TaLsi1 (ADM47602), TaTIP2;1 (AAS19468), TaTIP2;2 (AAS19469), TgTIP1;1 (BAL41683), TgTIP1;2 (BAL41684), ZmPIP1;1 (Q41870), ZmPIP1;5 (Q9AR14), ZmNIP2;1 (Q19KC1), ZmNIP2;2 (Q9ATN2).

Ammonia (NH_3) Transporters

```
JcNIP1;1        FPWKQVPAYIVCQVIGSTLAAGTIRLIFTGKQDHFVGTMPAGSNMQSFVVEFIITFYLMF
AtTIP2;3        ITLITGFFYWIAQCLGSIVACLLLVFVTNGKSVPTHGVSAGLGAVEGVVMEIVVTFALVY
AtTIP2;1        ITVITGVFYWIAQLLGSTAACFLLKYVTGGLAVPTHSVAAGLGSIEGVVMEIIITFALVY
TaTIP2;1        ITILTGIFYWVAQLLGAIVGAFLVQFCT-GVATPTHGLS-GVGAFEGVVMEIIVTFGLVY
TaTIP2;2        ITILTGIFYWVAQLLGAIVGAFLVQFCT-GVATPTHGLS-GVGAFEGVVMEIIVTFGLVY
GmNOD26         FPLIQVPAYVVAQLLGSILASGTLRLLFMGNHDQFSGTVPNGTNLQAFVFEFIMTFFLMF
                :.      * :.* :*:  ..  :     *      .       .:..*.*:::** *::

JcNIP1;1        VISGVATDNR--AIGELAGLAVGATVLLNVIFAGPISGASMNPARSLGPAIVSCKFKGLW
AtTIP2;3        TVYATAADPKKGSLGTIAPIAIGFIVGANILAAGPFSGGSMNPARSFGPAVVSGDLSQIW
AtTIP2;1        TVYATAADPKKGSLGTIAPLAIGLIVGANILAAGPFSGGSMNPARSFGPAVAAGDFSGHW
TaTIP2;1        TVYATAADPKKGSLGTIAPIAIGFIVGANILVAGPFSGGSMNPARSFGPAVASGDFTNIW
TaTIP2;2        TVYATAADPKKGSLGTIAPIAIGFIVGANILVAGPFSGGSMNPARSFGPAVASGDFTNIW
GmNOD26         VICGVATDNR--AVGEFAGIAIGSTLLLNVIIGGPVTGASMNPARSLGPAFVHGEYEGIW
                .: ..*:* :  ::* :* :*:*  :  *:: .**.:*.*******:***..  .    *

JcNIP1;1        IYLVSPTLGAQAGAWVYNMIRYTDKPLREITKSASFIKSTGRA-
AtTIP2;3        IYWVGPLVGGALAGLIYGDVFIGSYEAVETREIRV---------
AtTIP2;1        VYWVGPLIGGGLAGLIYGNVFMGSSEHVPLASADF---------
TaTIP2;1        VYWAGPLIGGGLAGVVYRYLYMCD-DHSSVAGNDY---------
TaTIP2;2        IYWAGPLIGGGLAGVVYRYVYMCD-DHSSVAGNDY---------
GmNOD26         IYLLAPVVGAIAGAWVYNIVRYTDKPLSETTKSASFLKGRAASK
                :*  .* :*.  .. :*  :   .       .
```

Boric Acid Transporters

```
JcPIP1;1        VGIQGIAWAFGGMIFALVYCTAGISGGHINPAVTFGLFLARKLSLTRAIYYMVMQCLGAI
JcPIP1;2        VGTQGIAWAFGGMIFALVYCTAGISGGHINPAVTFGLFLARKLSLTRALFYIIMQCLGAI
JcPIP1;3        VGIQGIAWAFGGMIFALVYCTAGISGGHINPA----------------------------
JcNIP2;1        LGAS---VAGGLIVTVMIYAVGHVSGAHMNPAVTTAFAAVREFPWKQVPFYAVAQMTGAI
JcNIP5;1        IGNA---ACSGLAVMIVILSTGHISGAHLNPALTIAFAALRHFPWVQVPAYIAAQVSASI
JcNIP6;1        VGLA---ASSGLAVMIVILSTGHISGAHLNPSVTIAFAALNHFPWKHVPVYIGAQVLASL
JcXIP2;1        LIMS---ALIAITVTILLIATSPISHGHINPVITFAALFTGRVSLSRAAVYILAQCLGAI
HvPIP1;3        VGIQGIAWSFGGMIFVLVYCTAGISGGHINPAVTFGLFLARKLSLTRAVFYIVMQCLGAI
HvPIP1;4        VGIQGIAWSFGGMIFVLVYCTAGISGGHINPAVTFGLFLARKLSLTRAVFYIVMQCLGAI
ZmPIP1;1        VGIQGIAWSFGGMILALVYCTAGISG-HINPAVTFGLFLARKLSLTRAVFYIIMQCLGAI
NtXIP1;1α       LIMS---ILIAIVITILLLAVVPVSGGHINPVISFSAALVGIISMSRAIIYMVAQCVGAI
NtXIP1;1β       LIMS---ILIAIVITILLLAVVPVSGGHINPVISFSAALVGIISMSRAIIYMVAQCVGAI
StXIP1;1α       LIMS---ILIAVVITILLLAVVPVSGGHINPVISFSAALVGIISMSRAIIYIVAQCLGAV
StXIP1;1β       LIMS---ILIAVVITILLLAVVPVSGGHINPVISFSAALVGIISMSRAIIYIVAQCLGAV
AtNIP5;1        IGNA---ACAGLAVMIIILSTGHISGAHLNPSLTIAFAALRHFPWAHVPAYIAAQVSASI
AtNIP6;1        IGCA---ASAGLAVMIVILSTGHISGAHLNPAVTIAFAALKHFPWKHVPVYIGAQVMASV
OsNIP2;1        LGQS---IAGGLIVTVMIYAVGHISGAHMNPAVTLAFAVFRHFPWIQVPFYWAAQFTGAI
                :         .  :  :: ..  :*  *:**

JcPIP1;1        CGAGVVKGFEGRKQYTLL-----------GGGANSVAPGYTKDGLGAEIVGTFVLVYTV
JcPIP1;2        CGAGVVKGFEGNRVYESL-----------GGGANVVASGYTKDGLGAEIVGTFVLVYTV
JcPIP1;3        -------GFEKN-QYERL-----------GGGANTISAGYSKDGLGAEIVGTFLLVYTV
JcNIP2;1        GASFTLKVLLHP-----------------IKQLGTTSPSGSDFQALVMEIVVTFSMMFVT
JcNIP5;1        CASFALKGVFHP-----------------FMSGGVTIPSVSTGQAFALEFLITFNLLFVV
JcNIP6;1        GAAFALKGILHP-----------------IMGGGVTVPSGGYGQAFALEFIISFNLMFVV
JcXIP2;1        LGALALKAVLNSTVEETFSLGGCTLSIVAPGPHGPILIGLETDRALWLEIICTFFFLFSS
HvPIP1;3        CGAGVVKGFQTT-LYQGN-----------GGGANSVAAGYTKDGLGAEIVGTFVLVYTV
HvPIP1;4        CGAGVVKGFQTT-LYQGN-----------GGGANSVAAGYTKDGLGAEIVGTFVLVYTV
ZmPIP1;1        CGRGVVKGFQQG-LYMGN-----------GGRRNVVAPGYTKDGLGAEIVGTFILVYTV
NtXIP1;1α      LGALALKAVVSSTIAQTFSLGGCTITVIAPGPNGPITVGLEMAQALWLEIFCTFVFLFAS
NtXIP1;1β      LGALALKAVVSSTIAQTFSLGGCTITVIAPGPNGPITVGLEMAQALWLEIFCTFVFLFAS
StXIP1;1α      LGALALRAVVSSSIEDTFSLGGCTVTIIAPGPNGPVTVGLETAQALWLEIFCTFVFLFAS
StXIP1;1β      LGALALRAVVSSSIEDTFSLGGCTVTIIAPGPNGPVTVGLETAQALWLEIFCTFVFLFAS
AtNIP5;1        CASFALKGVFHP-----------------FMSGGVTIPSVSLGQAFALEFIITFILLFVV
AtNIP6;1        SAAFALKAVFEP-----------------TMSGGVTVPTVGLSQAFALEFIISFNLMFVV
OsNIP2;1        CASFVLKAVIHP-----------------VDVIGTTTPVGPHWHSLVVEVIVTFNMMFVT
                       .                        .          .:   *..  :*  :::

JcPIP1;1        FSATDAKRNARDS---HVPILAPLPIGFAVFLVHLATIP--ITGTGINPARSLGAAIIFN
JcPIP1;2        FSATDAKRSARDS---HVPILAPLPIGFAVFLVHLATIP--ITGTGINPARSLGAAIIFN
JcPIP1;3        FSATDAKRNARDS---HVPILAPLPIGFAVFLVHLATIP--ITGTGINPARSLGAALIYN
JcNIP2;1        SAVATDTK--------AIGELAGIAVGSAVCITSILAGP--ISGGSMNPARTIGPAIASA
JcNIP5;1        TAVATDTR--------AVGELAGIAVGATVALNILVAGP--SSGASMNPVRTLGPAVAAG
JcNIP6;1        TAVATDTR--------AVGELAGIAVGATVMLNILIAGP--ATGASMNPVRTLGPAIAAN
JcXIP2;1        IWLAFDKRQSTPLGRVIVCCIIGLVVGLLVFISTTVTAQKGYAGVGMNPARCLGPALIRG
HvPIP1;3        FSATDAKRSARDS---HVPILAPLPIGFAVFLVHLATIP--ITGTGINPARSLGAAIIYN
HvPIP1;4        FSATDAKRSARDS---HVPILAPLPIGFAVFLVHLATIP--ITGTGINPARSLGAAIIYN
ZmPIP1;1        FSATDAKRRARDS---HVPILAPLPIGFAVFLVHLATMG--ITGTGINPARSLGAAVIYN
NtXIP1;1α       IWMAYDHRQAKALGLVTVLSIVGIVLGLLVFISTTVTMKKGYAGAGMNPARCFGAAVVRG
NtXIP1;1β       IWMAYDHRQAKALGLVTVLSIVGIVLGLLVFISTTVTMKKGYAGAGMNPARCFGAAVVRG
StXIP1;1α       IWMAYDHRQAKALGHVTVLSIVGLVLGLLVFISTTVTAKKGYGGAGINPARCLGPAIIRG
StXIP1;1β       IWMAYDHRQAKALGHVTVLSIVGLVLGLLVFISTTVTAKKGYGGAGINPARCLGPAIIRG
AtNIP5;1        TAVATDTR--------AVGELAGIAVGATVMLNILVAGP--STGGSMNPVRTLGPAVASG
AtNIP6;1        TAVATDTR--------AVGELAGIAVGATVMLNILIAGP--ATSASMNPVRTLGPAIAAN
OsNIP2;1        LAVATDTR--------AVGELAGLAVGSAVCITSIFAGA--ISGGSMNPARTLGPALASN
                   :   :         :  :  : :*  * :    :      .  .:**.* :*.*:
```

Carbon dioxide (CO_2) Transporters

```
JcPIP1;2         HINPAVTFGLFLARKLSLTRALFYIIMQCLGAICGAGVVKGFEGNRVYESLGGGANVVAS
AtPIP1;2         HINPAVTFGLFLARKLSLTRAVYYIVMQCLGAICGAGVVKGFQ-PKQYQALGGGANTIAH
NtAQP1           HINPAVTFGLFLARKLSLTRAIFYIVMQCLGAICGAGVVKGFM-VGPYQRLGGGANVVNH
HvPIP2;1         HINPAVTFGLFLARKVSLIRALLYIIAQCLGAICGVGLVKGFQ-SSYYVRYGGGANELSA
                 ***************:** **: **: ********.*:****     *   ***** :

JcPIP1;2         GYTKGDGLGAEIVGTFVLVYTVFSATDAKRSARDSHVPILAPLPIGFAVFLVHLATIPIT
AtPIP1;2         GYTKGSGLGAEIIGTFVLVYTVFSATDAKRNARDSHVPILAPLPIGFAVFLVHLATIPIT
NtAQP1           GYTKGDGLGAEIIGTFVLVYTVFSATDAKRNARDSYVPILAPLPIGFAVFLVHLATIPIT
HvPIP2;1         GYSKGTGLAAEIIGTFVLVYTVFSATDPKRNARDSHIPVLAPLPIGFAVFMVHLATIPIT
                 **:** **.***:**************.**.****::*:***********:*********

JcPIP1;2         GTGINPARSLGAAIIFNKDHAWDDHWVFWVGPFIGAALAALYHQIVIRAIPFKSRA----
AtPIP1;2         GTGINPARSLGAAIIFNKDNAWDDHWVFWVGPFIGAALAALYHVIVIRAIPFKSRS----
NtAQP1           GTGINPARSLGAAIIYNTDQAWDDHWIFWVGPFIGAALAAVYHQIIIRAIPFHKSS----
HvPIP2;1         GTGINPARSLGAAVIYNTDKAWDDQWIFWVGPLIGAAIAAAYHQYVLRASAAKLGSYRSN
                 *************:*:*.*:****:*:*****:****:** **  ::** . :  :
```

H_2O_2 Transporters

```
JcPIP1;1         QGIAWAFGGMIFALVYCTAGISGGHINPAVTFGLFLARKLSLTRAIYYMVMQCLGAICGA
JcPIP1;2         QGIAWAFGGMIFALVYCTAGISGGHINPAVTFGLFLARKLSLTRALFYIIMQCLGAICGA
JcPIP1;4         QGIAWAFGGMIFALVYSTAGISGGHINPAVTFGLLLARKVSLTRAIFYMVMQCLGAICGA
JcPIP2;1         LGIAWAFGGMIFILVYCTAGISGGHINPAVTFGLFLARKVSLVRAILYMAAQCLGAICGC
JcPIP2;2         LGIAWAFGGMIFILVYCTAGISGGHINPAVTFGLFLGRKVSLIRALGYMIAQCLGAICGC
JcPIP2;3         LGIAWAFGGMIFILVYCTAGISGGHINPAVTFGLFLARKVSLVRAVMYMVAQSLGAICGV
JcPIP2;4         LGIAWAFGGMIFILVYCTAGISGGHINPAVTFGLFLARKVSLIRALAYMVAQCLGAICGV
JcPIP2;5         LGVAWSFGSTIFILVYCTAGISGGHINPAVTFGLLLARKVSLNRAVAYIVAQCVGAIIGV
JcTIP1;2         VAASLAHAFALFVAVSVGANISGGHVNPAVTFGAFIGGNITLLRGILYWIAQLLGSVVAC
JcTIP3;1         VMIALAHALALFAAVAASINISGGHVNPAVTLGALVGGRISVVRAFYYWIAQLLGSIVAS
JcTIP5;1         IIVATANSFALSSAIYIAANISGGHVNPAVTFSMAVGGHISVPTALFYWISQMLASVMAC
JcNIP2;1         LGASVAGGLIVTVMIYAVGHVSGAHMNPAVTTAFAAVREFPWKQVPFYAVAQMTGAIGAS
JcNIP4;1         PGICVSWGLIVMVMIYTVGHVSGAHFNPAVTIASAIFRRFPFREVPLYIVAQVIGSILAS
JcNIP5;1         IGNAACSGLAVMIVILSTGHISGAHLNPALTIAFAALRHFPWVQVPAYIAAQVSASICAS
JcXIP2;1         LIMSALIAITVTILLIATSPISHGHINPVITFAALFTGRVSLSRAAVYILAQCLGAILGA
AtTIP1;1         VAAAVAHAFGLFVAVSVGANISGGHVNPAVTFGAFIGGNITLLRGILYWIAQLLGSVVAC
AtTIP1;2         VAAALAHAFGLFVAVSVGANISGGHVNPAVTFGVLLGGNITLLRGILYWIAQLLGSVAAC
TgTIP1;1         VAAAIAHAFALFVAVSVGANISGGHVNPAVTFGAFLGGNITLLRGVLYIIAQLLGSSVAC
TgTIP1;2         VAAAIAHAFALFVAVSVGANISGGHVNPAVTFGASLGGNITLLRGVLYIIAQLLGSSVAC
AtTIP2;3         VAIAIAHAFALFVGVSIAANISGGHLNPAVTLGLAIGGNITLITGFFYWIAQCLGSIVAC
AtPIP2;5         LGIAWAFGGMIFILVYCTAGISGGHINPAVTFGLLLARKVTLVRAVMYMVAQCLGAICGV
AtPIP2;1         LGIAWAFGGMIFILVYCTAGISGGHINPAVTFGLFLARKVSLPRALLYIIAQCLGAICGV
AtPIP2;2         LGIAWAFGGMIFILVYCTAGISGGHINPAVTFGLFLARKVSLIRAVLYMVAQCLGAICGV
AtPIP2;4         LGIAWAFGGMIFVLVYCTAGISGGHINPAVTVGLFLARKVSLVRTVLYIVAQCLGAICGC
ZmPIP2;5         LGIAWAFGGMIFILVYCTAGVSGGHINPAVTFGLFLARKVSLVRALLYIVAQCLGAICGV
AtPIP2;7         LGIAWAFGGMIFVLVYCTAGISGGHINPAVTFGLFLARKVSLVRALGYMIAQCLGAICGV
NtXIP1;1α        LIMSILIAIVITILLLAVVPVSGGHINPVISFSAALVGIISMSRAIIYMVAQCVGAILGA
NtXIP1;1β        LIMSILIAIVITILLLAVVPVSGGHINPVISFSAALVGIISMSRAIIYMVAQCVGAILGA
StXIP1;1α        LIMSILIAVVITILLLAVVPVSGGHINPVISFSAALVGIISMSRAIIYIVAQCLGAVLGA
StXIP1;1β        LIMSILIAVVITILLLAVVPVSGGHINPVISFSAALVGIISMSRAIIYIVAQCLGAVLGA
SlXIP1;1α        LIMSILIAVVITILLLAVVPVSGGHINPVISFSAALVGIISMSRAIIYIVAQCVGAILGA
SlXIP1;1β        LIMSILIAVVITILLLAVVPVSGGHINPVISFSAALVGIISMSRAIIYIVAQCVGAILGA
AtNIP1;2         PGIAIVWGLTVMVLVYSLGHISGAHFNPAVTIAFASCGRFPLKQVPAYVISQVIGSTLAA
                    .   .  :   :     :* .*.**.:: .      ..      *   *  .:  .
```

```
JcPIP1;1        GVVKGFEGRKQYTLLGGG-----------ANSVAPGYTKGDGLGAEIVGTFVLVYTVFSA
JcPIP1;2        GVVKGFEGNRVYESLGGG-----------ANVVASGYTKGDGLGAEIVGTFVLVYTVFSA
JcPIP1;4        GVVKGFQPTP-YQMGGGG-----------ANMVQPGYSKGDGLGAEIVGTFVLVYTVFSA
JcPIP2;1        GLVKAFQKAY-YTRYGGG-----------ANELADGYSKGTGLGAEIIGTFVLVYTVFSA
JcPIP2;2        GLVKAFQKAY-YNRYGGG-----------ANELADGYNKGTGLGAEIIGTFVLVYTVFSA
JcPIP2;3        GLVKAFQSAY-YKRYGGG-----------ANTLADGYSTGVGLGAEIIGTFVLVYTVFSA
JcPIP2;4        GLVKAFMKNP-YNHLGGG-----------ANSVNTGYSKGTALGAEIIGTFVLVYTVFSA
JcPIP2;5        ALVKGLVKDL-YKSLGGG-----------ANSVTAGFSIGTGLGVEILGTFFLEYTVLSA
JcTIP1;2        LLLKYATGGLETSAFALS----------------SGVSAWNAVVFEIVMTFGLVYTVYAT
JcTIP3;1        LLLRLVTNGMRPEGFHVT----------------AGVGEVHGLIMEIVMTFGLVYTVYAT
JcTIP5;1        VFLKVAIVGQNLPTYTIA----------------EEMTGFGASILEGVLTFGLVYTIYA-
JcNIP2;1        FTLKVLLHPIK-----------------QLGTTSPSGSDFQALVMEIVVTFSMMFVTSAV
JcNIP4;1        GTLALLFDITP----------------MAYFGTLPVGSNVQSLVIEIIITFLLMFVVSGV
JcNIP5;1        FALKGVFHPFM-----------------SGGVTIPSVSTGQAFALEFLITFNLLFVVTAV
JcXIP2;1        LALKAVLNSTVEETFSLGGCTLSIVAPGPHGPILIGLETDRALWLEIICTFFFLFSSIWL
AtTIP1;1        LILKFATGGLAVPAFGLS----------------AGVGVLNAFVFEIVMTFGLVYTVYAT
AtTIP1;2        FLLSFATGGEPIPAFGLS----------------AGVGSLNALVFEIVMTFGLVYTVYAT
TgTIP1;1        LLLRFTTG-LGTGTFGLV----------------AGVSVWSGLVMEIVMTFGLVYTVYAT
TgTIP1;2        LLLRFTTG-LGTGTFGLV----------------AGVSVWSGLVMEIVMTFGLVYTVYAT
AtTIP2;3        LLLVFVTNGKSVPTHGVS----------------AGLGAVEGVVMEIVVTFALVYTVYAT
AtPIP2;5        ALVKAFQSAY-FTRYGGG-----------ANGLSDGYSIGTGVAAEIIGTFVLVYTVFSA
AtPIP2;1        GFVKAFQSSY-YTRYGGG-----------ANSLADGYSTGTGLAAEIIGTFVLVYTVFSA
AtPIP2;2        GFVKAFQSSY-YDRYGGG-----------ANSLADGYNTGTGLAAEIIGTFVLVYTVFSA
AtPIP2;4        GFVKAFQSSY-YTRYGGG-----------ANELADGYNKGTGLGAEIIGTFVLVYTVFSA
ZmPIP2;5        GLVKGFQSAF-YVRYGGG-----------ANELSAGYSKGTGLAAEIIGTFVLVYTVFSA
AtPIP2;7        GFVKAFMKTP-YNTLGGG-----------ANTVADGYSKGTALGAEIIGTFVLVYTVFSA
NtXIP1;1α       LALKAVVSSTIAQTFSLGGCTITVIAPGPNGPITVGLEMAQALWLEIFCTFVFLFASIWM
NtXIP1;1β       LALKAVVSSTIAQTFSLGGCTITVIAPGPNGPITVGLEMAQALWLEIFCTFVFLFASIWM
StXIP1;1α       LALRAVVSSSIEDTFSLGGCTVTIIAPGPNGPVTVGLETAQALWLEIFCTFVFLFASIWM
StXIP1;1β       LALRAVVSSSIEDTFSLGGCTVTIIAPGPNGPVTVGLETAQALWLEIFCTFVFLFASIWM
SlXIP1;1α       LALRAVVSSSIEDTFSLGGCTVTIIAPGPNGPVIVGLETAQALWLEIFCTFVFLFASIWM
SlXIP1;1β       LALRAVVSSSIEDTFSLGGCTVTIIAPGPNGPVIVGLETAQALWLEIFCTFVFLFASIWM
AtNIP1;2        ATLRLLFGLDQDVCSGKH---------DVFVGTLPSGSNLQSFVIEFIITFYLMFVISGV
                  :                                      .   * . ** : :
```

```
JcPIP1;1        TDAKRNARDS---HVPILAPLPIGFAVFLVHLATIP--ITGTGINPARSLGAAIIFNKDQ
JcPIP1;2        TDAKRSARDS---HVPILAPLPIGFAVFLVHLATIP--ITGTGINPARSLGAAIIFNKDH
JcPIP1;4        TDAKRSARDS---HVPILAPLPIGFAVFVVNMATIP--ITGAGINPARSLGAAVIYNNDN
JcPIP2;1        TDPKRNARDS---HVPVLAPLPIGFAVFMVHLATIP--ITGTGINPARSFGAAVIYNKDK
JcPIP2;2        TDPKRNARDS---HVPVLAPLPIGFAVFMVHLATIP--ITGTGINPARSFGAAVIYNKDK
JcPIP2;3        TDPKRSARDS---HVPVLAPLPIGFAVFMVHLATIP--ITGTGINPARSLGAAVIYNHDK
JcPIP2;4        TDPKRSARDS---HVPILAPLPIGFAVFMVHLATIP--ITGTGINPARSFGAAVIYNNDK
JcPIP2;5        TDPKRKARDS---HVPVLAPLPIGFTVFVVHMATLP--ITGTGINPARSFGAAVIYNKKM
JcTIP1;2        AVDPKKGNLG------IIAPIAIGFIVGANILAGGA--FDGASMNPAVSFGPAVVSW---
JcTIP3;1        AIDPKRGSLG------IIAPLAIGLIVGANILVGGP--FDGAAMNPARAFGPALVGW---
JcTIP5;1        AGDPRRSLPG------AIGPLAIGLVAGANVLAAGP--FSGGSMNPASAFGSAVVAG---
JcNIP2;1        ATDTK--AIG------ELAGIAVGSAVCITSILAGP--ISGGSMNPARTIGPAIASA---
JcNIP4;1        NTDDR--AVG------DLGGIAVGMTILLNVFVAGP--VSGASMNPARSLGPAIVKH---
JcNIP5;1        ATDTR--AVG------ELAGIAVGATVALNILVAGP--SSGASMNPVRTLGPAVAAG---
JcXIP2;1        AFDKRQSTPLGRVIVCCIIGLVVGLLVFISTTVTAQKGYAGVGMNPARCLGPALIRG-GH
AtTIP1;1        AIDPKNGSLG------TIAPIAIGFIVGANILAGGA--FSGASMNPAVAFGPAVVSW---
AtTIP1;2        AVDPKNGSLG------TIAPIAIGFIVGANILAGGA--FSGASMNPAVAFGPAVVSW---
TgTIP1;1        AVDPKKGDIG------TIAPIAIGFIVGANILVGGA--FTGASMNPAIAFGPALVSW---
TgTIP1;2        AVDPKKGDIG------TIAPIAIGFIVGANILVGGA--FTGASMNPAIAFGPALVSW---
AtTIP2;3        AADPKKGSLG------TIAPIAIGFIVGANILAAGP--FSGGSMNPARSFGPAVVSG---
AtPIP2;5        TDPKRSARDS---HVPVLAPLPIGFAVFIVHLATIP--ITGTGINPARSLGAAIIYNKDK
AtPIP2;1        TDPKRSARDS---HVPVLAPLPIGFAVFMVHLATIP--ITGTGINPARSFGAAVIYNKSK
AtPIP2;2        TDPKRNARDS---HVPVLAPLPIGFAVFMVHLATIP--ITGTGINPARSFGAAVIYNKSK
AtPIP2;4        TDPKRNARDS---HVPVLAPLPIGFAVFMVHLATIP--ITGTGINPARSFGAAVIYNNEK
ZmPIP2;5        TDPKRNARDS---HVPVLAPLPIGFAVFMVHLATIP--ITGTGINPARSLGAAVIYNNDK
AtPIP2;7        TDPKRSARDS---HIPVLAPLPIGFAVFMVHLATIP--ITGTGINPARSFGAAVIYNNEK
NtXIP1;1α       AYDHRQAKALGLVTVLSIVGIVLGLLVFISTTVTMKKGYAGAGMNPARCFGAAVVRG-GH
NtXIP1;1β       AYDHRQAKALGLVTVLSIVGIVLGLLVFISTTVTMKKGYAGAGMNPARCFGAAVVRG-GH
StXIP1;1α       AYDHRQAKALGHVTVLSIVGLVLGLLVFISTTVTAKKGYGGAGINPARCLGPAIIRG-GH
StXIP1;1β       AYDHRQAKALGHVTVLSIVGLVLGLLVFISTTVTAKKGYGGAGINPARCLGPAIIRG-GH
SlXIP1;1α       AYDHRQAKALGHVTVLSIVGLVLGLLVFISTTVTAKKGYGGAGINPARCLGPAIIRG-GH
SlXIP1;1β       AYDHRQAKALGHVTVLSIVGLVLGLLVFISTTVTAKKGYGGAGINPARCLGPAIIRG-GH
AtNIP1;2        ATDNR--AIG------ELAGLAVGSTVLLNVIIAGP--VSGASMNPGRSLGPAMVYS---
                    :            :  : :*                * .:**   :*.*:
```

```
JcPIP1;1          GWDDHWIFWVGPFIGAALAALYHQVVIRAIPFKK--------------------------
JcPIP1;2          AWDDHWVFWVGPFIGAALAALYHQIVIRAIPFKSRA------------------------
JcPIP1;4          GWDDHWIFWVGPFIGAALAALYHQIVIRAIPFKSRA------------------------
JcPIP2;1          AWDDQWIFWVGPFIGAAIAAIYHQYILRAGAVKALGSFRSTSNV----------------
JcPIP2;2          AWDDQWIFWVGPFVGAAIAAFYHQYILRAAAIKALGSFRSNA------------------
JcPIP2;3          AWDDQWIFWVGPFLGAAIAAFYHQFILRAGAVKALGSFRSNPTV----------------
JcPIP2;4          VWDDHWIFWVGPFVGAIAAAAYHQYILRAAAIKALGSFRSNPTN----------------
JcPIP2;5          VWDDHWIFWVGPLVGAAAAAAYHQIVLRAGAVKALGSFRGNPVM----------------
JcTIP1;2          TWTNHWVYWVGPLIGAAIAALVYDNIFIGEG-----AHEPLS--TSDF------------
JcTIP3;1          RWSNHWIYWVGPFIGGALAGLIYEYMVIPTEPPLHHTHQPLA--PEDY------------
JcTIP5;1          RFKNQAVYWVGPLIGATVAGLLYDNVVFPN-----QVPDSIRGISDGVRV----------
JcNIP2;1          YYKGIWVYIVGPVAGTLLGAWSYNLIRVTDKPVQAISPSFSFRIRRTSIDEQTNNKDPLS
JcNIP4;1          TYKGLWVYIAGPIVGAIAGAFAYNLLRSTDKPLDDE------------------------
JcNIP5;1          NYKAIWIYLVAPILGGLAGGTTYTAVKLREDEADPPRQVRSFRR----------------
JcXIP2;1          LWSGHWVFWVGPIISCIAFGVYVKIIP----------------VANA-------------
AtTIP1;1          TWTNHWVYWAGPLVGGGIAGLIYEVFFINT------THEQLP--TTDY------------
AtTIP1;2          TWTNHWVYWAGPLIGGGLAGIIYDFVFIDEN-----AHEQLP--TTDY------------
TgTIP1;1          SWTSHWVYWVGPLIGGGIAGIVYDLFFINS------THEQLP--STDY------------
TgTIP1;2          SWASHWVYWVGPLIGGGIAAIVYDLFFISG------THEQLP--TTDY------------
AtTIP2;3          DLSQIWIYWVGPLVGGALAGLIYGDVFIGS-------YEAVE--TREIRV----------
AtPIP2;5          AWDHHWIFWVGPFAGAAIAAFYHQFVLRAGAIKALGSFRSQPHV----------------
AtPIP2;1          PWDDHWIFWVGPFIGAAIAAFYHQFVLRASGSKSLGSFRSAANV----------------
AtPIP2;2          PWDDHWIFWVGPFIGAAIAAFYHQFVLRASGSKSLGSFRSAANV----------------
AtPIP2;4          AWDDQWIFWVGPMIGAAAAAFYHQFILRAAAIKALGSFGSFGSFRSFA------------
ZmPIP2;5          AWDDHWIFWVGPFIGAAIAAAYHQYVLRASAAK-LGSSASFSR-----------------
AtPIP2;7          AWDDQWIFWVGPFLGALAAAAYHQYILRASAIKALGSFRSNATN----------------
NtXIP1;1α         LWDGHWIFWVGPTIACVAFYVYTKIIPPKHFHAD-GYKYDFIGVVKASFGLHE-------
NtXIP1;1β         LWDGHWIFWVGPTIACVAFYVYTKIIPPKHFHAD-GYKYDFIGVVKASFGLHE-------
StXIP1;1α         LWDGHWIFWVGPTIGCVAFYVYTKIIPPKHFLAEYGFKHDFVGVVKALSNV---------
StXIP1;1β         LWDGHWIFWVGPTIGCVAFYVYTKIIPPKHFLAEYGFKHDFVGVVKALSNV---------
SlXIP1;1α         LWDGHWIFWVGPTIGCVAFYVYTKIIPTKHFLAEYGFKHDFVGVVKALSNV---------
SlXIP1;1β         LWDGHWIFWVGPTIGCVAFYVYTKIIPTKHFLAEYGFKHDFVGVVKALSNV---------
AtNIP1;2          CYRGLWIYIVSPIVGAVSGAWVYNMVRYTDKPLREITKSGSFLKTVRNGSSR--------
                        :: ..*  .          .
```

Silicic Acid Transporters

```
JcNIP2;1          VREFPWKQVPFYAVAQMTGAIGASFTLKVLLHPIKQLGTTSPSGSDFQALVMEIVVTFSM
HvNIP2;1          FRHFPWIQVPFYWAAQFTGAICASFVLKAVLHPITVIGTTEPVGPHWHALVIEVVVTFNM
TaLsi1            FRHFPWIQVPFYWAAQFTGAICASFVLKAVLHPITVIGTTEPVGPHWHALVIEVVVTFNM
ZmNIP2;1          FRHFPWIQVPFYWAAQFTGSICASFVLKAVLHPIAVLGTTTPTGPHWHSLVIEIIVTFNM
OsNIP2;1          FRHFPWIQVPFYWAAQFTGAICASFVLKAVIHPVDVIGTTTPVGPHWHSLVVEVIVTFNM
ZmNIP2;2          FRHFPWIQVPFYWAAQFTGAMCAAFVLKAVLHPIAVIGTTTPSGPHWHALLIEIVVTFNM
OsNIP2;2          FRHFPWIQVPFYWAAQFTGAMCAAFVLRAVLYPIEVLGTTTPTGPHWHALVIEIVVTFNM
CmNIP2;1a         TRHFPWKQVPLYAAAQLSGATCAAFTLRLLLHPIKHLGTTTPSGSDLQALVMEIVVTFSM
CpNIP1            TRHFPWKQVPLYAAAQLSGATCAAFTLRLLLHPIKHLGTTTPSGSDLQALVMEIVVTFSM
CmNIP2;1b         TRHFPWKQVPLYAAAQLSGATCAAFTLRLLLHPIKHLGTTTPSGSDLQALVMEIVVTFSM
GmNIP2;1          VRHLPWPQLPFYIAAQLTGAISASYTLRELLRPSNEIGGTSPAGSHIQALIMEMVTTYTM
GmNIP2;2          VRHLPWPQLPFYVAAQLTGAISASYTLRELLRPSDEIGGTSPAGSHIQALIMEMVSTYTM
                   **:** ::* * ***::*: .*::.*: :: *   :* * * *.. ::*::*:: *: *
```

```
JcNIP2;1        MFVTSAVATDTKAIGELAGIAVGSAVCITSILAGPISGGSMNPARTIGPAIASAYYKGIW
HvNIP2;1        MFVTLAVATDTRAVGELAGLAVGSSVCITSIFAGAVSGGSMNPARTLGPALASNRYPGLW
TaLsi1          MFVTLAVATDTRAVGELAGLAVGSSVCITSIFAGAVSGGSMNPARTLGPALASNRYPGLW
ZmNIP2;1        MFVTLAVATDTRAVGELAGLAVGSAVCITSIFAGAVSGGSMNPARTLGPALASNLYTGLW
OsNIP2;1        MFVTLAVATDTRAVGELAGLAVGSAVCITSIFAGAISGGSMNPARTLGPALASNKFDGLW
ZmNIP2;2        MFVTCAVATDSRAVGELAGLAVGSAVCITSIFAGPVSGGSMNPARTLAPAVASNVFTGLW
OsNIP2;2        MFVTCAVATDSRAVGELAGLAVGSAVCITSIFAGPVSGGSMNPARTLAPAVASNVYTGLW
CmNIP2;1a       MFVTCAVATDTKAVGELAGLAVGSAVCITSILAGPVSGGSMNPVRTLGPAMASDNYKGLW
CpNIP1          MFVTCAVATDTKAVGELAGLAVGSAVCITSILAGPVSGGSMNPVRTLGPAMASDNYKGLW
CmNIP2;1b       MFVTCAVATDTKAVGELAGLAVGSAVCITSILAGPVSGGSMNPVRTLGPAMASDNYKGLW
GmNIP2;1        VFISMAVATDSNATGQLSGVAVGSSVCIASIVAGPISGGSMNPARTLGPAIATSYYKGLW
GmNIP2;2        VFISMAVATDSNATGQLSGVAVGSSVCIASIVAGPISGGSMNPARTLGPAIATSYYKGLW
                :*:: *****:.* *:*:*:****:***:**.**.:*******.***.**:*:  : *:*

JcNIP2;1        VYIVGPVAGTLLGAWSYNLIRVTDKPVQAIS--P-SFS-FRIRRT-SI-DEQTNNKDPLS
HvNIP2;1        LYFLGPVLGTLSGAWTYTYIRFEDPP--KDA--PQKLSSFKLRRLQSQ-SVAADD-DELD
TaLsi1          LYFLGPVLGTLSGAWTYTYIRFEDPP--KDG--PQKLSSFKLRRLQSQ-SVAADD-DELD
ZmNIP2;1        IYFLGPVLGTLSGAWTYTYIRFEEAPSHKDM--SQKLSSFKLRRLQSQ-SVAVDD-DELD
OsNIP2;1        IYFLGPVMGTLSGAWTYTFIRFEDTP-KEGS--SQKLSSFKLRRLRSQQSIAADDVDEME
ZmNIP2;2        IYFLGPVIGTLSGAWVYTYIRFEEAPAAKD---TQRLSSFKLRRMQSQ--LAADEFDTV-
OsNIP2;2        IYFLGPVVGTLSGAWVYTYIRFEEAPAAAGGAAPQKLSSFKLRRLQSQ-SMAADEFDNV-
CmNIP2;1a       VYFVGLVTGTLLGAWSYKFIRASDKPVHLIS--PHSFS-LKLRRMSRS-DVGEGER----
CpNIP1          VYFVGPVTGTLLGAWSYKFIRASDKPVHLIS--PHSFS-LKLRRMSRS-DVGEGER----
CmNIP2;1b       VYFVGPVTGTLLGAWSYKFIRASDKPVHLIS--PHSFS-LKLRRMSRS-DVGEGER----
GmNIP2;1        VYFVGPITGAVLAAWSYNVIRDTEHPGFPIS--LSSIS-SKVRQSIGGTEQKSDQRCLV-
GmNIP2;2        VYFVGPITGAVLAAWSYNVIRDTEHPGFPIS--LSSIS-SKVRQSIGGTEQKSDQRCLV-
                :*::* : *:: .:* *. **  : *          :*  *:*:          :
```

Urea Transporters

```
JcPIP1;1        ------CSTVGIQGIAWAFGGMIFALVYCTAGISGGHINPAVTFGLFLARKLSLTRAIYY
JcPIP1;2        ------CATVGTQGIAWAFGGMIFALVYCTAGISGGHINPAVTFGLFLARKLSLTRALFY
JcPIP1;4        ------CATVGVQGIAWAFGGMIFALVYSTAGISGGHINPAVTFGLLLARKVSLTRAIFY
JcPIP2;1        SKNSDACGGVGILGIAWAFGGMIFILVYCTAGISGGHINPAVTFGLFLARKVSLVRAILY
JcPIP2;2        NKNADACGGVGILGIAWAFGGMIFILVYCTAGISGGHINPAVTFGLFLGRKVSLIRALGY
JcPIP2;3        AKNADSCGGVGILGIAWAFGGMIFILVYCTAGISGGHINPAVTFGLFLARKVSLVRAVMY
JcPIP2;4        ------CGGVGLLGIAWAFGGMIFILVYCTAGISGGHINPAVTFGLFLARKVSLIRALAY
JcPIP2;5        SG----CGGVGLLGVAWSFGSTIFILVYCTAGISGGHINPAVTFGLLLARKVSLNRAVAY
JcTIP1;1        ----GATTPAGLVAASLAHGFGLFVAVSVGANISGGHVNPAVTFGAFVGGNITLLRGILY
JcTIP1;2        ----GSSTPAGLVAASLAHAFALFVAVSVGANISGGHVNPAVTFGAFIGGNITLLRGILY
JcTIP1;3        ----GSTTPAGLVAASLAHGFALFVAVSVGANISGGHVNPAVTFGAFVGGHITLMRSILY
JcTIP1;4        ----GSSTPAGIIMASLAHAFGLFIGVATAANISGGHVNPAVTFGAFVGGNITLLRGILY
JcTIP2;1        ----AALDPAGLVAIAICHGFALFVAVAVGANISGGHVNPAVTFGLALGGQITILTGIFY
JcTIP2;2        ----AALDPPGLVAVAVAHAFALFVGVSIAANISGGHLNPAVTFGLAIGGHITLLTGLFY
JcTIP4;1        -------PLVGLFFVAVAHALVVAVMISAG-HISGGHLNPAVTLGLLFGGHITLVRSILY
JcTIP5;1        -----AADPSSLIIVATANSFALSSAIYIAANISGGHVNPAVTFSMAVGGHISVPTALFY
JcNIP1;1        ----NFDKVVTHPGISIVWGLAVMVLVYSVGHISGAHFNPAVTLAFATCKRFPWKQVPAY
JcNIP2;1        ----SDEKRVSELGASVAGGLIVTVMIYAVGHVSGAHMNPAVTTAFAAVREFPWKQVPFY
JcNIP3;1        ----IQK--LTILGIAIVWGVVLMALIYAVGHISGAHFNPAVSIALAAVRKFSWKHVPVF
JcNIP4;1        ----IYG-SVTFPGICVSWGLIVMVMIYTVGHVSGAHFNPAVTIASAIFRRFPFREVPLY
JcNIP5;1        ----KYSGVETLIGNAACSGLAVMIVILSTGHISGAHLNPALTIAFAALRHFPWVQVPAY
JcNIP6;1        ----KTQGAETLVGLAASSGLAVMIVILSTGHISGAHLNPSVTIAFAALNHFPWKHVPVY
ZmPIP1;5        ------CATVGIQGIAWSFGGMIFALVYCTAGISGGHINPAVTFGLFLARKLSLTRALFY
NtAQP1          ------CSSVGIQGVAWAFGGMIFALVYCTAGISGGHINPAVTFGLFLARKLSLTRAIFY
AtTIP1;1        ----GATTPSGLVAAAVAHAFGLFVAVSVGANISGGHVNPAVTFGAFIGGNITLLRGILY
AtTIP1;2        ----GATTPSGLVAAALAHAFGLFVAVSVGANISGGHVNPAVTFGVLLGGNITLLRGILY
AtTIP1;3        ----GPATPAGLVAASLSHAFALFVAVSVGANVSGGHVNPAVTFGAFIGGNITLLRAILY
AtTIP2;1        ----AALDTPGLVAIAVCHGFALFVAVAIGANISGGHVNPAVTFGLAVGGQITVITGVFY
AtTIP4;1        -------TLVGLFAVAVAHAFVVAVMISAG-HISGGHLNPAVTLGLLLGGHISVFRAFLY
NtTIPa          -------PLVSLFFVAMAHALVVAVTISAGFRISGGHLNPAVTLGLCMGGHITVFRSILY
AtTIP5;1        ----DVSGPFGVLIPAIANALALSSSVYISWNVSGGHVNPAVTFAMAVAGRISVPTAMFY
CpNIP1          ----SDAQRVSQLGASVAGGLIVTVMIYAVGHISGAHMNPAVTTAFAATRHFPWKQVPLY
OsNIP2;1        ----SDLSRISQLGQSIAGGLIVTVMIYAVGHISGAHMNPAVTLAFAVFRHFPWIQVPFY
AtNIP6;1        ----KTDGAETLIGCAASAGLAVMIVILSTGHISGAHLNPAVTIAFAALKHFPWKHVPVY
NtXIP1;1α       ------DVKMPNLIMSILIAIVITILLLAVVPVSGGHINPVISFSAALVGIISMSRAIIY
NtXIP1;1β       ------DVKMPNLIMSILIAIVITILLLAVVPVSGGHINPVISFSAALVGIISMSRAIIY
StXIP1;1α       ------DTKMPNLIMSILIAVVITILLLAVVPVSGGHINPVISFSAALVGIISMSRAIIY
StXIP1;1β       ------DTKMPNLIMSILIAVVITILLLAVVPVSGGHINPVISFSAALVGIISMSRAIIY
                               .   .  :   :     :**.*.** :: .      ..      :

JcPIP1;1        MVMQCLGAICGAGVVKGFEGRKQYTLLGGGAN-----------SVAPGYTKGDGLGAEIV
JcPIP1;2        IIMQCLGAICGAGVVKGFEGNRVYESLGGGAN-----------VVASGYTKGDGLGAEIV
JcPIP1;4        MVMQCLGAICGAGVVKGFQPT-PYQMGGGGAN-----------MVQPGYSKGDGLGAEIV
JcPIP2;1        MAAQCLGAICGCGLVKAFQKA-YYTRYGGGAN-----------ELADGYSKGTGLGAEII
JcPIP2;2        MIAQCLGAICGCGLVKAFQKA-YYNRYGGGAN-----------ELADGYNKGTGLGAEII
JcPIP2;3        MVAQSLGAICGVGLVKAFQSA-YYKRYGGGAN-----------TLADGYSTGVGLGAEII
JcPIP2;4        MVAQCLGAICGVGLVKAFMKN-PYNHLGGGAN-----------SVNTGYSKGTALGAEII
JcPIP2;5        IVAQCVGAIIGVALVKGLVKD-LYKSLGGGAN-----------SVTAGFSIGTGLGVEIL
JcTIP1;1        WIAQLLGSTVACLLLKFSTGGL---TTSAFA-------------LSSGVGVWNAFVFEIV
JcTIP1;2        WIAQLLGSVVACLLLKYATGGL---ETSAFA-------------LSSGVSAWNAVVFEIV
JcTIP1;3        WVGQLLGSVVACLLLKFATGGL---ETSAFA-------------LSSGVSSWNALVFEIV
JcTIP1;4        WIAQLLGSTVACLLLKFSTHGM---TTSAFA-------------LSSGVNVWNALVFEIV
JcTIP2;1        WIAQLLGSIVACLLLKVVTGGL---ETPTHS-------------LAAGVGAIEGVVMEII
JcTIP2;2        WIAQSLGSIVACLLLKFVTNGK---SIPTHG-------------VASGVNAFEGVVFEIV
JcTIP4;1        WIDQLLASSAACLLLNYLTGGL---ATPVHT-------------LASGVGYLQGVVWEII
JcTIP5;1        WISQMLASVMACVFLKVAIVGQ---NLPTYT-------------IAEEMTGFGASILEGV
JcNIP1;1        IVCQVIGSTLAAGTIRLIFTGK---QDHFVG-------------TMPAGSNMQSFVVEFI
JcNIP2;1        AVAQMTGAIGASFTLKVLLHPI----KQLGT-------------TSPSGSDFQALVMEIV
JcNIP3;1        VLAQVLGSTLAILTLKVLFHDQDDIQATMTQ-------------YKNSTSHLEAIIWEFI
JcNIP4;1        IVAQVIGSILASGTLALLFDIT---PMAYFG-------------TLPVGSNVQSLVIEII
```

```
JcNIP5;1        IAAQVSASICASFALKGVFHPF----MSGGV-------------TIPSVSTGQAFALEFL
JcNIP6;1        IGAQVLASLGAAFALKGILHPI----MGGGV-------------TVPSGGYGQAFALEFI
ZmPIP1;5        MVMQCLGAICGAGVVKGFQEG-LYMGAGGGAN-----------AVNPGYTKGDGLGAEIV
NtAQP1          IVMQCLGAICGAGVVKGFMVG-PYQRLGGGAN-----------VVNHGYTKGDGLGAEII
AtTIP1;1        WIAQLLGSVVACLILKFATGGL---AVPAFG-------------LSAGVGVLNAFVFEIV
AtTIP1;2        WIAQLLGSVAACFLLSFATGGE---PIPAFG-------------LSAGVGSLNALVFEIV
AtTIP1;3        WIAQLLGAVVACLLLKVSTGGM---ETAAFS-------------LSYGVTPWNAVVFEIV
AtTIP2;1        WIAQLLGSTAACFLLKYVTGGL---AVPTHS-------------VAAGLGSIEGVVMEII
AtTIP4;1        WIDQLLASSAACFLLSYLTGGM---GTPVHT-------------LASGVSYTQGIIWEII
NtTIPa          WIDQLLASVAACALLNYLTAGL---ETPVHT-------------LANGVSYGQGIIMEVI
AtTIP5;1        WTSQMIASVMACLVLKVTVMEQ---HVPIYK-------------IAGEMTGFGASVLEGV
CpNIP1          AAAQLSGATCAAFTLRLLLHPI----KHLGT-------------TTPSGSDLQALVMEIV
OsNIP2;1        WAAQFTGAICASFVLKAVIHPV----DVIGT-------------TTPVGPHWHSLVVEVI
AtNIP6;1        IGAQVMASVSAAFALKAVFEPT----MSGGV-------------TVPTVGLSQAFALEFI
NtXIP1;1α       MVAQCVGAILGALALKAVVSSTIAQTFSLGGCTITVIAPGPNGPITVGLEMAQALWLEIF
NtXIP1;1β       MVAQCVGAILGALALKAVVSSTIAQTFSLGGCTITVIAPGPNGPITVGLEMAQALWLEIF
StXIP1;1α       IVAQCLGAVLGALALRAVVSSSIEDTFSLGGCTVTIIAPGPNGPVTVGLETAQALWLEIF
StXIP1;1β       IVAQCLGAVLGALALRAVVSSSIEDTFSLGGCTVTIIAPGPNGPVTVGLETAQALWLEIF
                   *  .:  .   :                                      .   * .

JcPIP1;1        GTFVLVYTVFSATDAKRNARDS---HVPILAPLPIGFAVFLVHLATIPI--TGTGINPAR
JcPIP1;2        GTFVLVYTVFSATDAKRSARDS---HVPILAPLPIGFAVFLVHLATIPI--TGTGINPAR
JcPIP1;4        GTFVLVYTVFSATDAKRSARDS---HVPILAPLPIGFAVFVVNMATIPI--TGAGINPAR
JcPIP2;1        GTFVLVYTVFSATDPKRNARDS---HVPVLAPLPIGFAVFMVHLATIPI--TGTGINPAR
JcPIP2;2        GTFVLVYTVFSATDPKRNARDS---HVPVLAPLPIGFAVFMVHLATIPI--TGTGINPAR
JcPIP2;3        GTFVLVYTVFSATDPKRSARDS---HVPVLAPLPIGFAVFMVHLATIPI--TGTGINPAR
JcPIP2;4        GTFVLVYTVFSATDPKRSARDS---HVPILAPLPIGFAVFMVHLATIPI--TGTGINPAR
JcPIP2;5        GTFFLEYTVLSATDPKRKARDS---HVPVLAPLPIGFTVFVVHMATLPI--TGTGINPAR
JcTIP1;1        MTFGLVYTVYATAIDPKKG------SLGTIAPIAIGFIVGANILAGGAF--DGASMNPAV
JcTIP1;2        MTFGLVYTVYATAVDPKKG------NLGIIAPIAIGFIVGANILAGGAF--DGASMNPAV
JcTIP1;3        MTFGLVYTVYATAVDPRKG------NLGTIAPIAIGFIVGANILAGGAF--DGASMNPAV
JcTIP1;4        MTFGLVYTVYATAIDPRKG------QLGTIAPIAIGFIVGANVLAGGAF--EGASMNPAV
JcTIP2;1        VTFALVYTVYATAADPKKG------SLGTIAPIAIGFIVGANILAAGPF--SGGSMNPAR
JcTIP2;2        ITFGLVYTVYATAADPKKG------SLGIIAPIAIGFIVGANILAAGPF--SGGSMNPAR
JcTIP4;1        LTFSLLFTVYATIVDPKKG------AIDGLGPTLTGFVVGANILAGGAF--SGASMNPAR
JcTIP5;1        LTFGLVYTIYA-AGDPRRS------LPGAIGPLAIGLVAGANVLAAGPF--SGGSMNPAS
JcNIP1;1        ITFYLMFVISGVATDNR--------AIGELAGLAVGATVLLNVIFAGPI--SGASMNPAR
JcNIP2;1        VTFSMMFVTSAVATDTK--------AIGELAGIAVGSAVCITSILAGPI--SGGSMNPAR
JcNIP3;1        ITFILMFNICAVATDHR--------ASKDFSGVAIGGTLLVNVMVAGPI--TGASMNPAR
JcNIP4;1        ITFLLMFVVSGVNTDDR--------AVGDLGGIAVGMTILLNVFVAGPV--SGASMNPAR
JcNIP5;1        ITFNLLFVVTAVATDTR--------AVGELAGIAVGATVALNILVAGPS--SGASMNPVR
JcNIP6;1        ISFNLMFVVTAVATDTR--------AVGELAGIAVGATVMLNILIAGPA--TGASMNPVR
ZmPIP1;5        GTFVLVYTVFSATDAKRSARDS---HVPILAPLPIGFAVFLVHLATIPI--TGTGINPAR
NtAQP1          GTFVLVYTVFSATDAKRNARDS---YVPILAPLPIGFAVFLVHLATIPI--TGTGINPAR
AtTIP1;1        MTFGLVYTVYATAIDPKNG------SLGTIAPIAIGFIVGANILAGGAF--SGASMNPAV
AtTIP1;2        MTFGLVYTVYATAVDPKNG------SLGTIAPIAIGFIVGANILAGGAF--SGASMNPAV
AtTIP1;3        MTFGLVYTVYATAVDPKKG------DIGIIAPLAIGLIVGANILVGGAF--DGASMNPAV
AtTIP2;1        ITFALVYTVYATAADPKKG------SLGTIAPLAIGLIVGANILAAGPF--SGGSMNPAR
AtTIP4;1        LTFSLLFTVYATIVDPKKG------SLDGFGPLLTGFVVGANILAGGAF--SGASMNPAR
NtTIPa          LTFSLLFTVYTTIVDPKKG------ILEGMGPLLTGLVVGANIMAGGPF--SGASMNPAR
AtTIP5;1        LAFVLVYTVFT-ASDPRRG------LPLAVGPIFIGFVAGANVLAAGPF--SGGSMNPAC
CpNIP1          VTFSMMFVTCAVATDTK--------AVGELAGLAVGSAVCITSILAGPV--SGGSMNPVR
OsNIP2;1        VTFNMMFVTLAVATDTR--------AVGELAGLAVGSAVCITSIFAGAI--SGGSMNPAR
AtNIP6;1        ISFNLMFVVTAVATDTR--------AVGELAGIAVGATVMLNILIAGPA--TSASMNPVR
NtXIP1;1α       CTFVFLFASIWMAYDHRQAKALGLVTVLSIVGIVLGLLVFISTTVTMKKGYAGAGMNPAR
NtXIP1;1β       CTFVFLFASIWMAYDHRQAKALGLVTVLSIVGIVLGLLVFISTTVTMKKGYAGAGMNPAR
StXIP1;1α       CTFVFLFASIWMAYDHRQAKALGHVTVLSIVGLVLGLLVFISTTVTAKKGYGGAGINPAR
StXIP1;1β       CTFVFLFASIWMAYDHRQAKALGHVTVLSIVGLVLGLLVFISTTVTAKKGYGGAGINPAR
                 :* : :         :            .     *                . .:**.
```
